# ﻿Delimitation of the widely distributed Palearctic *Stenodema* species (Hemiptera, Heteroptera, Miridae): insights from molecular and morphological data

**DOI:** 10.3897/zookeys.1209.124766

**Published:** 2024-08-13

**Authors:** Anna A. Namyatova, Polina A. Dzhelali, Fedor V. Konstantinov

**Affiliations:** 1 Zoological Institute, Russian Academy of Sciences, Universitetskaya nab. 1, St. Petersburg 199034, Russia Zoological Institute, Russian Academy of Sciences St. Petersburg Russia; 2 All-Russian Institute of Plant Protection, Podbelskogo sh. 3, Pushkin, St. Petersburg, 196608, Russia All-Russian Institute of Plant Protection St. Petersburg Russia; 3 National Museum of Natural History, Bulgarian Academy of Sciences, 1 Tsar Osvoboditel Blvd, 1000 Sofia, Bulgaria National Museum of Natural History, Bulgarian Academy of Sciences Sofia Bulgaria

**Keywords:** Holarctic, phylogeny, plant bugs, species delimitation, taxonomy

## Abstract

Species delimitation presents a significant challenge in biology, particularly in systematics. Here, an integrative approach is employed to assess the species boundaries of widely distributed Palearctic *Stenodema* species. Due to their diversity, wide distribution, and the absence of comprehensive morphological and molecular data for most species, revising *Stenodema* is both daunting and time-consuming. Our study focuses on detailed examinations of male and female genitalia, coupled with phylogenetic analyses based on two mitochondrial markers (cytochrome c oxidase subunit I and 16S rRNA) and species delimitation analyses. Eight species with wide distributions are reviewed, *Stenodematrispinosa* Reuter, 1904 is synonymized with *S.pilosa* (Jakovlev, 1889), and a lectotype for *Stenodematuranica* Reuter, 1904 is designated. Morphological and molecular data effectively distinguish all species, revealing distinct clades and relationships. Notably, *S.calcarata* and *S.pilosa* form a well-supported clade, while *S.virens* and *S.turanica* share a lineage with Nearctic species. *Stenodemarubrinervis* and *S.sibirica* are morphologically similar and form a distinct clade in all phylogenies. Species delimitation analyses confirm the separation of all studied species, and genetic distances suggest the potential existence of cryptic species within *S.calcarata* and *S.pilosa*. This study highlights the advantages of integrative taxonomy in delimiting species with intricate and relatively recent phylogeographic histories.

## ﻿Introduction

Taxonomy and biodiversity of different organisms, including insects, is well studied in the Palearctic. However, the boundaries of many groups and their interrelationships are solely addressed using morphological characters. Although the number of taxonomic works based on molecular data is increasing, studies on species inhabiting both Europe and Asia are scarce. Asian taxa, and those having trans-Holarctic distribution, also remain understudied (e.g., Hortal et al. 2015; Pante et al. 2015; Satler et al. 2021). Such works require relatively fresh material collected from different localities in areas spanning thousands of kilometers. The task of obtaining such specimens is difficult, expensive, and time-consuming, and may not always be feasible. Nevertheless, molecular studies of widespread species are important, because this helps to understand the population structure of such groups, reveal the presence of cryptic species and possible synonymy of other species. The poor knowledge of the widely distributed species might negatively affect further studies in other fundamental and applied fields, such as biodiversity, phylogeography, ecology, evolution, and conservation (e.g., Angulo and Icochea 2010; Taberlet et al. 2012; Namyatova et al. 2023).

Miridae or plant bugs are among the largest insect families and their representatives are abundant and play important roles in many ecosystems. This group is considered well studied in the Palearctic and Nearctic especially in comparison with subtropical and tropical regions (Cassis and Schuh 2012). There are several keys to species published in the 20^th^ century focusing on Europe or Asia (e.g., Kerzhner and Jaczewski 1964; Wagner and Weber 1964; Wagner 1974; Kerzhner 1988; Vinokurov and Kanyukova 1995), and numerous genera have been lately revised (e.g., Namyatova and Konstantinov 2009; Namyatova 2010; Matocq and Pluot-Sigwalt 2012; Knyshov and Konstantinov 2013a, 2013b; Konstantinov 2008, 2019; Konstantinov et al. 2016; Davletshin and Konstantinov 2024). However, these works are solely based on morphology, and to date, only two studies have been performed addressing species delimitation in plant bugs using combined morphological and molecular data (Sanchez and Cassis 2018; Namyatova et al. 2023). There is only a single work attempting to separate trans-Palearctic species with molecular markers, which showed that the morphological and molecular data did not correspond to each other (Namyatova et al. 2023). Miridae also include several trans-Holarctic species (Kerzhner and Josifov 1999), and the species with such distribution was addressed in the previous study (Namyatova et al. 2023).

*Stenodema* Laporte, 1832 is a large genus, distributed in the Palearctic, South Asia, South and North America, and South Africa. It is included into the tribe Stenodemini within the largest plant bug subfamily Mirinae and is distinguished from other members of its tribe by several morphological characters (Schwartz 1987, 2008). The representatives of this genus are elongate with green, yellow, or brown coloration, generally associated with graminoid monocots, and some of its species are considered pests (Wheeler 2001; Yasunaga 2019). *Stenodema* currently includes 57 species and 37 of them have been described from the Holarctic region, 31 of them inhabit the Palearctic (Schuh 2013; Yasunaga 2019). Some of those taxa are known only from short original descriptions. There are also a number of widespread species: *Stenodemacalcarata* (Fallén, 1807), *S.holsata* (Fabricius, 1787), *S.laevigata* (Linnaeus, 1758), *S.pilosa* (Jakovlev, 1889), *S.sibirica* Bergroth, 1914, *S.trispinosa* Reuter, 1904, *S.turanica* Reuter, 1904, and *S.virens* (Linnaeus, 1767), which might potentially represent a complex of cryptic species. Among them, *S.calcarata* and *S.holsata* are trans-Palearctic and *S.trispinosa* has trans-Holarctic distribution. *Stenodemalaevigata* and *S.virens* are mostly known from the Western Palearctic, *S.turanica* inhabits Balkans, Caucasus, Middle East, Central Asia, and China, *S.pilosa* was recorded from the south of European Russia, Ukraine, Caucasus, Turkey, Central Asia, and China, while *S.sibirica* inhabits Siberia and East Asia. The identification keys for those species were mostly based on the external morphological characters, and their genitalia were poorly studied. The barcoding region of cytochrome c oxidase subunit I (COI) has been provided for some species, but as a part of the regional barcoding projects (Jung et al. 2011; Raupach et al. 2014; Kim and Jung 2018; Roslin et al. 2022). The intraspecific genetic variation within *Stenodema* species has not been studied and, therefore, it remains uncertain whether the barcoding region can be used for species delimitation.

The diversity and wide distribution of widespread *Stenodema* species, coupled with the limited morphological details and absence of molecular data for most representatives of this genus, make the revisionary work on *Stenodema* difficult and time-consuming. The first step towards the revision of this genus is a detailed study of the widely distributed species and providing the morphological and molecular data for them, which can be a background for further comparisons. In this study we evaluated the species boundaries of the widely distributed Palearctic species of *Stenodema*. We studied their male and female genitalia, provided the phylogeny based on the two mitochondrial markers (COI and 16S rRNA), and performed species delimitation analyses.

## ﻿Materials and methods

### ﻿Specimens

The specimens from the historical collection of the Zoological Institute of the Russian Academy of Sciences, St Petersburg, Russia (**ZISP**) and recently collected material were examined. Type specimens of *Stenodema* spp. retained in the Finnish Museum of Natural History (**MZH**) were also studied. The specimens were initially identified using the keys published in Kerzhner and Jaczewski (1964), Wagner (1974), Vinokurov and Kanyukova (1995), and Yasunaga (2019). The following number of specimens were examined for this study: *Stenodemacalcarata* (71), *S.holsata* (46), *S.laevigata* (52), *S.rubrinervis* (12), *S.pilosa* (13), *S.sibirica* (50), *S.trispinosa* (64), *S.turanica* (41) and *S.virens* (39). The collection event data for all of them were entered to the Arthropod Easy Capture Database (https://research.amnh.org/pbi/locality/index.php) and available through the Heteroptera Species Pages (https://research.amnh.org/pbi/heteropteraspeciespage/speciesdetails.php). All specimens were examined externally, and at least 10 males and 10 females from different series for each species were dissected for examination of the genitalia. The list of non-type specimens examined for this study is provided in Suppl. material 1.

For the molecular studies, the specimens from the following species were used: *S.calcarata* (13 specimens). *S.holsata* (4 specimens), *S.laevigata* (11 specimens), *S.trispinosa* (3 specimens), *S.turanica* (3 specimens), *S.virens* (3 specimens), *Leptopternadolobrata* (Linneaus, 1758) (1 specimen) and *Trigonotylus* sp. (1 specimen). The genitalia structures were examined for all *Stenodema* vouchers.

### ﻿Dissections, drawings, and terminology

To examine the male and female genitalia structures, abdomens were removed and boiled in 10% KOH for up to five minutes and dissected in water. Afterward, the abdomens were stored in glycerol. In some cases, aedeagi were inflated after this procedure. Aedeagi were also inflated using 40% lactic acid, following the detailed procedure described in Namyatova et al. (2021). The drawings were completed using Leica DM2500 microscope with the drawing device attached. The terminology of genitalia follows Konstantinov (2000, 2003) for males and Schwartz (2008) for females.

The digital images were taken in stacks using the Canon EOS 5D Mark IV camera equipped with a Canon MP-E 65 mm f/2.8 1–5× Macro lens and a Twin-Lite MT-26EX-RT flash. Partially focused images were combined using the Helicon Focus software. The SEM images were taken from uncoated specimens using the Hitachi TM1000 tabletop microscope.

### ﻿Measurements

Measurements were completed using Micromed MS-5 microscope using a graticule and ×10 eyepiece. Measurements statistics is provided in Table 1. Scale bars for habitus images equal 1 mm, the scale bars for genitalia structures equal 0.1 mm. Measurements provided in the diagnoses and descriptions are in mm.

**Table 1.** Measurements for *Stenodema* species.

Species		Length					Width		
		Body	Cun-Clyp	Pronotum	AntSeg1	AntSeg2	Head	Pronotum	InterOcDi
*S.calcarata*	Mean	6.13	4.48	0.92	0.82	2.15	0.77	1.22	0.38
♂ (*n* = 7)	SD	0.23	0.15	0.02	0.03	0.20	0.03	0.04	0.02
	Range	0.58	0.42	0.04	0.08	0.52	0.10	0.10	0.04
	Min	5.92	4.33	0.90	0.79	1.90	0.71	1.15	0.35
	Max	6.50	4.75	0.94	0.88	2.42	0.81	1.25	0.40
♀ (*n* = 7)	Mean	6.26	4.68	0.98	0.85	1.89	0.80	1.32	0.43
	SD	0.28	0.09	0.05	0.03	0.14	0.02	0.06	0.02
	Range	0.92	0.25	0.10	0.06	0.38	0.04	0.15	0.06
	Min	5.75	4.58	0.94	0.83	1.75	0.79	1.25	0.40
	Max	6.67	4.83	1.04	0.90	2.13	0.83	1.40	0.46
*S.holsata*									
♂ (*n* = 7)	Mean	5.18	3.95	0.85	0.75	1.68	0.79	1.20	0.40
	SD	0.51	0.28	0.07	0.06	0.11	0.04	0.10	0.03
	Range	1.25	0.83	0.23	0.17	0.33	0.13	0.27	0.06
	Min	4.67	3.58	0.75	0.71	1.54	0.75	1.08	0.38
	Max	5.92	4.42	0.98	0.88	1.88	0.88	1.35	0.44
♀ (*n* = 7)	Mean	5.88	4.52	1.01	0.79	1.66	0.87	1.43	0.47
	SD	0.37	0.27	0.05	0.05	0.14	0.04	0.09	0.02
	Range	0.92	0.83	0.10	0.15	0.31	0.13	0.25	0.04
	Min	5.50	4.17	0.96	0.75	1.56	0.81	1.31	0.46
	Max	6.42	5.00	1.06	0.90	1.88	0.94	1.56	0.50
*S.laevigata*									
♂ (*n* = 7)	Mean	6.45	5.00	1.03	1.04	2.19	0.77	1.21	0.41
	SD	0.32	0.20	0.06	0.02	0.07	0.03	0.05	0.01
	Range	0.83	0.58	0.17	0.06	0.17	0.06	0.13	0.02
	Min	5.92	4.58	0.94	1.02	2.13	0.75	1.13	0.40
	Max	6.75	5.17	1.10	1.08	2.29	0.81	1.25	0.42
♀ (*n* = 7)	Mean	7.10	5.40	1.17	1.07	2.20	0.83	1.37	0.45
	SD	0.26	0.20	0.04	0.05	0.09	0.03	0.05	0.02
	Range	0.67	0.58	0.10	0.13	0.21	0.10	0.13	0.06
	Min	6.83	5.00	1.13	1.00	2.08	0.77	1.31	0.42
	Max	7.50	5.58	1.23	1.13	2.29	0.88	1.44	0.48
*S.sibirica*									
♂ (*n* = 7)	Mean	6.08	4.38	0.96	0.80	1.86	0.79	1.28	0.41
	SD	0.28	0.17	0.04	0.03	0.11	0.02	0.04	0.01
	Range	0.67	0.50	0.13	0.08	0.31	0.04	0.13	0.02
	Min	5.83	4.17	0.92	0.75	1.77	0.77	1.21	0.40
	Max	6.50	4.67	1.04	0.83	2.08	0.81	1.33	0.42
♀ (*n* = 7)	Mean	6.50	4.80	1.08	0.83	1.94	0.87	1.51	0.49
	SD	0.20	0.26	0.03	0.02	0.12	0.03	0.08	0.03
	Range	0.58	0.83	0.08	0.06	0.33	0.08	0.23	0.06
	Min	6.25	4.50	1.04	0.81	1.81	0.83	1.35	0.46
	Max	6.83	5.33	1.13	0.88	2.15	0.92	1.58	0.52
*S.trispinosa*									
♂ (*n* = 7)	Mean	5.81	4.24	0.88	0.74	1.95	0.78	1.24	0.40
	SD	0.44	0.31	0.07	0.03	0.20	0.06	0.03	0.03
	Range	1.33	0.92	0.21	0.10	0.54	0.15	0.08	0.08
	Min	5.08	3.75	0.77	0.69	1.73	0.71	1.21	0.35
	Max	6.42	4.67	0.98	0.79	2.27	0.85	1.29	0.44
♀ (*n* = 7)	Mean	6.23	4.70	1.01	0.74	1.67	0.81	1.38	0.43
	SD	0.14	0.36	0.05	0.04	0.08	0.03	0.05	0.01
	Range	0.42	1.00	0.13	0.10	0.23	0.06	0.13	0.04
	Min	6.00	4.33	0.94	0.69	1.52	0.77	1.29	0.42
	Max	6.42	5.33	1.06	0.79	1.75	0.83	1.42	0.46
*S.turanica*									
♂ (*n* = 7)	Mean	6.33	4.95	1.00	0.82	2.73	0.83	1.38	0.36
	SD	0.25	0.23	0.10	0.02	0.16	0.03	0.08	0.01
	Range	0.75	0.75	0.23	0.04	0.48	0.08	0.21	0.02
	Min	6.08	4.58	0.90	0.79	2.60	0.79	1.31	0.35
	Max	6.83	5.33	1.13	0.83	3.08	0.88	1.52	0.38
♀ (*n* = 7)	Mean	7.07	5.61	1.15	0.82	2.19	0.86	1.53	0.43
	SD	0.27	0.49	0.07	0.01	0.16	0.04	0.07	0.03
	Range	0.75	1.33	0.21	0.02	0.46	0.13	0.17	0.08
	Min	6.58	4.92	1.04	0.81	1.92	0.79	1.44	0.40
	Max	7.33	6.25	1.25	0.83	2.38	0.92	1.60	0.48
*S.virens*									
♂ (*n* = 7)	Mean	6.36	4.74	1.11	0.74	2.07	0.82	1.39	0.40
	SD	0.20	0.21	0.04	0.03	0.09	0.02	0.04	0.02
	Range	0.58	0.50	0.10	0.06	0.23	0.04	0.10	0.04
	Min	6.00	4.50	1.04	0.71	1.96	0.79	1.33	0.38
	Max	6.58	5.00	1.15	0.77	2.19	0.83	1.44	0.42
♀ (*n* = 7)	Mean	6.80	5.06	1.13	0.73	1.92	0.82	1.45	0.44
	SD	0.39	0.22	0.11	0.04	0.15	0.05	0.12	0.03
	Range	1.00	0.67	0.31	0.13	0.40	0.13	0.29	0.10
	Min	6.08	4.58	0.94	0.67	1.69	0.75	1.27	0.38
	Max	7.08	5.25	1.25	0.79	2.08	0.88	1.56	0.48
*S.rubrinervis*									
♂ (*n* = 7)	Mean	6.93	5.42	0.99	1.04	2.69	0.85	1.40	0.40
	SD	0.45	0.60	0.07	0.05	0.16	0.02	0.11	0.01
	Range	1.25	1.58	0.19	0.15	0.52	0.06	0.33	0.02
	Min	6.25	4.58	0.92	0.98	2.46	0.83	1.27	0.40
	Max	7.50	6.17	1.10	1.13	2.98	0.90	1.60	0.42
♀ (*n* = 7)	Mean	7.49	6.05	1.15	1.14	2.57	0.92	1.48	0.50
	SD	0.10	0.38	0.07	0.05	0.13	0.04	0.07	0.03
	Range	0.33	1.00	0.21	0.13	0.35	0.10	0.21	0.06
	Min	7.33	5.50	1.04	1.10	2.35	0.88	1.38	0.46
	Max	7.67	6.50	1.25	1.23	2.71	0.98	1.58	0.52

### ﻿DNA protocols and sequencing

The DNA was extracted from abdomens of ethanol-stored and dry specimens using the Evrogen Extract DNA Blood and Cells kit. The standard protocol was used with two modifications. First, the abdomens were kept overnight in the lysis solution with proteinase K in the water bath. Second, 50 or 25 μl of elution buffer was added at the final stage to increase the DNA concentration. After lysis, the abdomens were kept in glycerol for further examination. To obtain the barcoding region of cytochrome c oxidase subunit I (COI) the primers from Vishnevskaya et al (2016) were used with the annealing temperature equaling 45 °C or 42 °C. To obtain 16S rRNA region, the primers from Menard et al. (2014) were used with the annealing temperature 48 °C. For both markers, temperature of the initial denaturation and denaturation was 94 °C (3 mins and 30 secs, respectively), and extension and final extension temperature was 68 °C (1 min and 10 mins, respectively). The PCR products were cleaned using Evrogen Clean-up S-Cap kits or with Exonuclease I Thermofisher and sequenced in Evrogen (https://evrogen.ru/). The products were between 647 to 847 for COI and between 361 to 403 for 16s rRNA. The base pairs were trimmed at both ends if they were absent in more than half of the sequences in the alignment. The sequences were uploaded to GenBank, the accession numbers are listed in the Suppl. material 2.

The sequence diversity was calculated using P-distance and Kimura-2-parameter (K2P) in MEGA-X (Tamura et al. 2021) within each species, between species and between the clades within species.

Alignments were completed using Geneious algorithm in Geneious v. 11 software for each marker separately. Alignments included 36 original COI and 16S rRNA each. The COI alignment also included 84 sequences downloaded from Genbank: *S.calcarata* (15), *S.holsata* (17), *S.laevigata* (17), *S.pilosipes* (2), *S.rubrinervis* (5), *S.sericans* (3), *S.sibirica* (4), *S.trispinosa* (15), *S.vicina* (5), *S.virens* (1). Alignment for 16s rRNA additionally included four sequences of *S.rubrinervis* (2) and *S.sibirica* (2) from GenBank. Both alignments included original sequences of two outgroup taxa, *Leptopternadolobrata* and *Trigonotylus* sp. All GenBank accession numbers are listed in the Suppl. material 1. Two alignments were concatenated using Geneious. The alignment lengths for COI and 16s rRNA were 787 bp and 399 bp, respectively. Phylogenetic analyses were run on each marker separately and for the combined datasets. Both combined datasets were 1186 bp length. First of them included all sequences available and included 124 terminals (full dataset). The second dataset included 34 specimens for which both markers were obtained (reduced dataset). In all the cases, *Trigonotylus* sp. was chosen as a root.

Maximum Likelihood approach implemented in RAxML v. 8.2.12 (Stamatakis 2014) with 10000 bootstrap replicates (BS) was performed. The phylogenetic trees were also calculated using Bayesian inference with MrBayes v. 3.2.7 (Ronquist et al. 2012). The main settings for MrBayes included 20 million generations, four chains, and the burn-in was set at 25%. Posterior probabilities were used for the node support (PP). Log files were checked to ensure that the standard deviation of split frequencies reached 0.01. All analyses were run using the server Dell PowerEdge R7525 (Dell Inc., USA).

Automatic barcode gap discovery approach (ABGD) was used via the online tool (https://bioinfo.mnhn.fr/abi/public/abgd/abgdweb.html) on the alignment of each marker separately. This algorithm searches for a gap, which can be observed whenever the divergence among organisms belonging to the same species is smaller than the divergence among organisms from different species (Puillandre et al. 2012). The P range was set at 0.001–0.01, and Kimura (K80) model was used to estimate the matrix of pairwise distances.

Poisson tree process model (PTP and bPTP) and Generalized Mixed Yule Coalescent approach (bGMYC) were applied to the phylogenies built on a single marker and on combined datasets. Both approaches model the transition in branch length between species in contrast to within species (e.g., Blair and Bryson 2017) as another indication of speciation events. GMYC is a model-based likelihood approach that combines phylogenetics and coalescence theory, was proposed to estimate species boundaries from DNA sequence data. This algorithm identifies the transition points between inter- and intra-species branching rates on a time-calibrated ultrametric tree by maximizing the likelihood score of the model (Pons et al. 2006; Reid and Carstens 2012; Fujisawa and Barraclough 2013). PTP approach does not need an ultrametric tree and model speciation rate by directly using the number of substitutions (Zhang et al. 2013).

For all analyses, bGMYC, PTP, and bPTP, only unique sequences were left in the datasets, because zero-length branches can affect the results (Reid and Carstens 2012). The duplicates were removed using the online tool sRNAtoolbox (Aparicio-Puerta et al. 2022) (https://arn.ugr.es/srnatoolbox/helper/removedup/). It is recommended to run the species delimitation analysis based on several trees, which helps to overcome the problems with the phylogenetic uncertainty, occurring when the species delimitation is applied for the single tree (Reid and Carstens 2012; da Silva et al. 2018). The trees were calculated using BEAST2 v. 2.6.3 software (Bouckaert et al. 2014) using GTR+G+I nucleotide substitution model with 50 mln chain length. The results were checked in Tracer v. 1.7.1.(Rambaut et al. 2018) to make sure that all parameters had effective sampling size exceeded 200, which is considered adequate for convergence (https://beast.community/analysing_beast_output). The LogCombiner application from the BEAST package was used to obtain the .tre file with ~ 100 trees for each case.

Species delimitation using GMYC was run in R with the bGMYC package with the parameters recommended in the instructions (http://nreid.github.io/assets/bGMYC_instructions_14.03.12.txt), the multiple thresholds was used, MCMC equaled 50000, and thinning equaled 40000. This analysis provides the list of all possible species, and we have chosen the set of species with the highest mean supports.

Bayesian and Maximum Likelihood implementations of the Poisson tree process model (PTP and bPTP) (Zhang et al. 2013) using the scripts in Python (https://github.com/zhangjiajie/PTP accessed in 31/10/2021) were used. The number of iterations equaled 100000. All analyses were run using the server Dell PowerEdge R7525 (Dell Inc., USA).

Bayesian Phylogenetics and Phylogeography (BPP) method tests species using the multispecies coalescent model (Yang 2015), and it was applied to the combined datasets, which includes both, COI and 16s rRNA. It tests whether the separated species has higher supports than the clade comprising combination of species. The specimens should be preliminary assigned to a putative species for this analysis. For each dataset, the specimens were assigned to species based on the phylogenetic results and the bGMYC, PTP, and bPTP analyses ran on the corresponding dataset. The root was removed from the datasets. The analysis was run through the interface version for Windows (https://abacus.gene.ucl.ac.uk/software.html). The A11 (species delimitation and species tree) analysis with nsamples = 50000, sampfreq = 2, burnin = 25000 was applied. All other settings were default.

## ﻿Results

### ﻿Morpho-taxonomic account

Our study showed that most of the widely distributed Palearctic species can be separated from each other using external characters, as well as male and female genitalia. The diagnoses for those species are provided in this section.

Below we provide the key to species, where we included all widely distributed Palearctic species. We also added *S.algoviensis* Schmidt, 1934 (Central Europe), *S.alpestris* Reuter, 1904 (China), *S.chinensis* Reuter, 1904 (China), *S.crassipes* Kiritshenko, 1931 (Central Asia), *S.khenteica* Muminov, 1989 (Mongolia), *S.plebeja* Reuter, 1904 (China), *S.rubrinervis* Horváth, 1905 (China, Korea, and Japan), and *S.sericans* (Fieber, 1861) (Europe) to this key, because we had an opportunity to examine them. *Stenodemanippon* Yasunaga, 2019 was included, as Yasunaga (2019) provided a detailed illustrated description for this species. Thus, the key is designed to discriminate all *Stenodema* spp. of the Western Palearctic, Siberia, and the Far East. However, it does not include 16 of the 19 species originally described and currently known only from China. For a taxonomic account of Chinese species of *Stenodema*, refer to Zheng et al. (2004). Species comparisons are provided following the diagnoses.

### ﻿Key to species

**Table:** 

1	Frons not protruding above clypeus (Fig. 1H, I)	**2**
–	Frons protruding above clypeus (Fig. 1C)	**9**
2	Spines on hind femur present (Fig. 2A, D); swelling above propleural apodeme straight (Fig. 1I)	**3**
–	Spines on hind femur absent (Fig. 2B, C, E, F, H); swelling above propleural apodeme curved (Fig. 1H)	**4**
3	Hind femur with three spines ventroapically (Fig. 2D); right paramere L-shaped, not bifurcate apically (Fig. 6N); vesica lobes without large spinulate outgrowth (Fig. 3G–I); sclerotized rings on dorsal labiate plate ~ 2–2.5× as wide as long (Fig. 4H)	***S.pilosa***
–	Hind femur with two spines and small, barely recognizable tubercle ventroapically (Fig. 2A); right paramere bifurcate apically (Fig. 5I); left vesica lobe with large spinulate outgrowth (Fig. 3B); sclerotized rings on dorsal labiate plate ~ 3× as wide as long (Fig. 4A)	***S.calcarata***
4	Hind femur distinctly tapering apically (Fig. 2E)	***S.laevigata***
–	Hind femur straight or slightly tapering apically (Fig. 2H)	**5**
5	Hemelytron yellow, without contrasting marking along inner margin; pronotum with calli brown to dark brown, but without longitudinal paired dark brown stripes; hind femora without rows of dark markings	***S.sericans***
–	Hemelytron often with contrasting marking along inner margin; pronotum with paired longitudinal markings; hind femur often with rows of dark markings	**6**
6	Flattened silver setae on hemelytron present	***S.chinensis***
–	Only simple setae on hemelytron present	**7**
7	Antennal segment II/head width ratio in female > 2.7; body length/pronotum width ratio 4.9–5.0; left paramere only slightly inclined basally (Fig. 5N, Wagner 1974: fig. 90E, F)	***S.plebeja***
–	Antennal segment I/head width ratio in female 1.7–2.2; body length/pronotum width ratio 3.9–4.3; left paramere distinctly curved basally (Wagner 1974: figs 5N, 90E, F)	**8**
8	Antennal segment II/vertex width in male 4.0–4.4; left paramere with additional elongate swelling near apical process (Fig. 4N)	***S.holsata***
–	Antennal segment II/vertex width in male 5.0; left paramere with small swelling near apical process (Tamanini 1982: fig. 2A)	***S.algoviensis***
9	Hind femur straight apically with rare setae on posterior side (as in Fig. 2H)	**10**
–	Hind femur tapering apically with dense setae on posterior side (Fig. 2B, C)	**13**
10	Antennal segment I longer than mesal length of pronotum	***S.nippon***
–	Antennal segment I shorter or as long as mesal length of pronotum	**11**
11	Antennal segment I narrower than eye diameter, and as wide as hind femur	***S.khenteica***
–	Antennal segment I as wide as eye diameter, and narrower than hind femur	**12**
12	Antennal segment II/pronotum width ratio in male 1.4–1.6, in female 1.2–1.4, antennal segment II/head width ratio in male 2.2–2.6, in female 2.2–2.5; vertex width/eye ratio in male 2.1–2.4, antennal segment I/head width ratio in female 0.9–1; body length 5.8–6.5 in male, 6.3–6.8 in female	***S.sibirica***
–	Antennal segment II/pronotum width ratio in male 1.7–2.2, in female 1.6–1.9, antennal segment I/head width ratio in male 3.0–3.3, in female 2.7–2.9; vertex width/eye ratio in male 1.7–2.0; antennal segment I/head width ratio in female 1.2–1.3; body length 6.2–7.5 in male, 7.3–7.7 in female	***S.rubrinervis* , *S.alpestris***
13	Hind femur distinctly enlarged, 4–5× as long as wide, antennal segment II in female widened basally with long and dense setae; antennal segment III shorter than vertex	***S.crassipes***
–	Hind femur not enlarged, 6–8× as long as wide; antennal segment II not widened basally with short setae; antennal segment III as long as or longer than vertex	**14**
14	Antennal segment II in male 2.4–2.6× as long as head width; vesica with four lobes (Fig. 7G–I); membranous swelling on dorsal labiate plate large, partly covering sclerotized rings (Fig. 10C)	***S.virens***
–	Antennal segment II in males 3.1–3.5× as long as head width; vesica with five lobes (Figs 7D–F; 8A); membranous swelling on dorsal labiate plate not covering sclerotized rings (Fig. 10A)	***S.turanica***

#### ﻿ Stenodema calcarata

Taxon classificationAnimaliaHemipteraMiridae

(Fallén, 1807)

BD201B71-C602-5362-B384-B874760320D5

**Figure 1.**
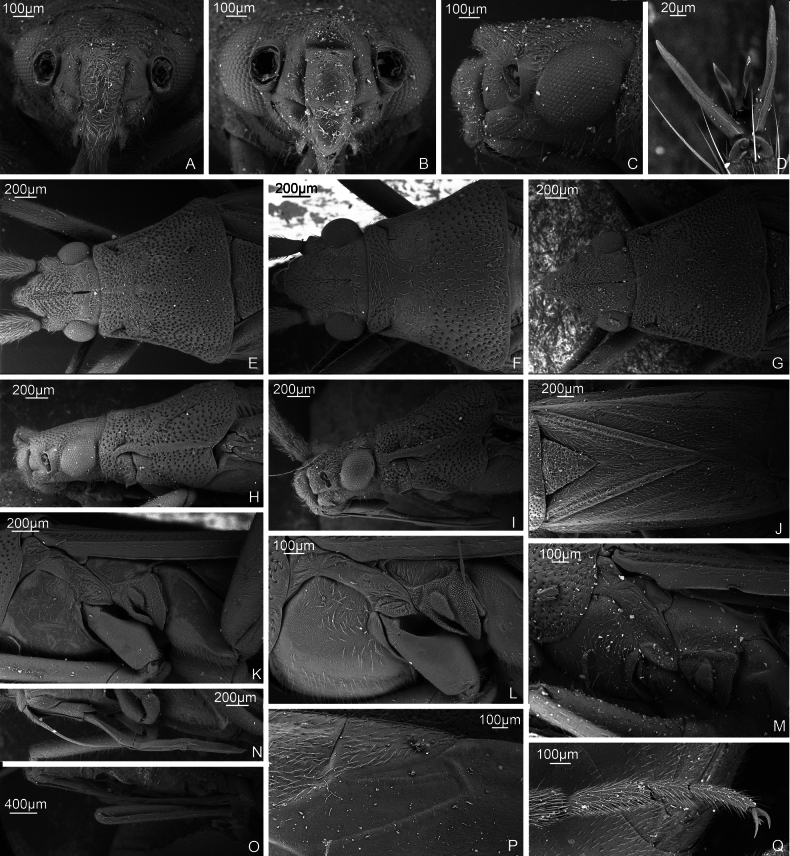
SEM images. *S.pilosa***A** head, anterior view. ZISP_ENT 00009372 **G** head and pronotum, dorsal view, ZISP_ENT 00009372 **Q** hind tarsus, ZISP_ENT 00009386. *S.turanica***B** head, anterior view, ZISP_ENT 00004934 **C** head, lateral view, ZISP_ENT 00004934. *S.holsata***D** pretarsus, dorsal view, ZISP_ENT 00013676 **F** head and pronotum, dorsal view, ZISP_ENT 00007905. *S.calcarata***E** head and pronotum, dorsal view, ZISP_ENT 00007331 **I** head and pronotum, lateral view, ZISP_ENT 00013671 **L** thoracic pleura, ZISP_ENT 00007386 **N** labium, ZISP_ENT 00007382. *S.laevigata***H** head and pronotum, lateral view, ZISP_ENT 00005650 **K** thoracic pleura, ZISP_ENT 00007921 **O** labium, ZISP_ENT 00013673. *S.virens***J** scutellum, clavus. and corium, ZISP_ENT 00003645 **P** cuneus and membrane, ZISP_ENT 00003645. *S.sibirica***M** thoracic pleura, ZISP_ENT 00004930.

**Figure 2.**
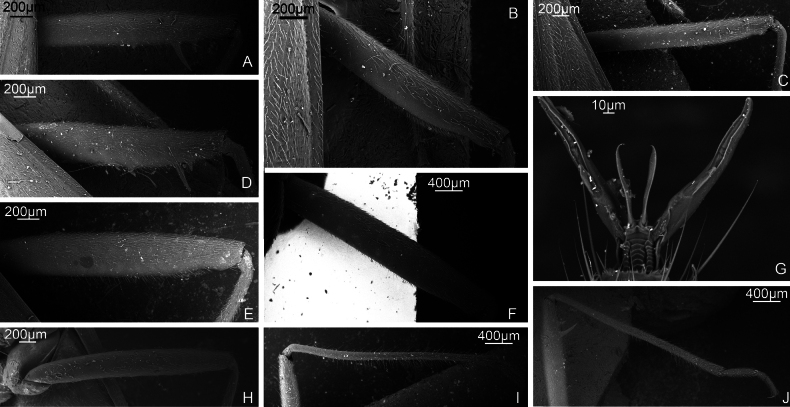
SEM images. *S.calcarata***A** hind femur, ZISP_ENT 00007331 **G** pretarsus ventrally, ZISP_ENT 00013668 **J** hind tibia, ZISP_ENT 00007331. *S.virens***B** hind femur, ZISP_ENT 00003645. *S.turanica***C** hind femur, ZISP_ENT 00004938 **I** hind tibia, ZISP_ENT 00004938. *S.pilosa***D** hind femur, ZISP_ENT 00009371. *S.laevigata***E** hind femur, ZISP_ENT 00006444. *S.sibirica***F** hind femur, ZISP_ENT 00003705. *S.holsata***H** hind femur, ZISP_ENT 00013674.

**Figure 3.**
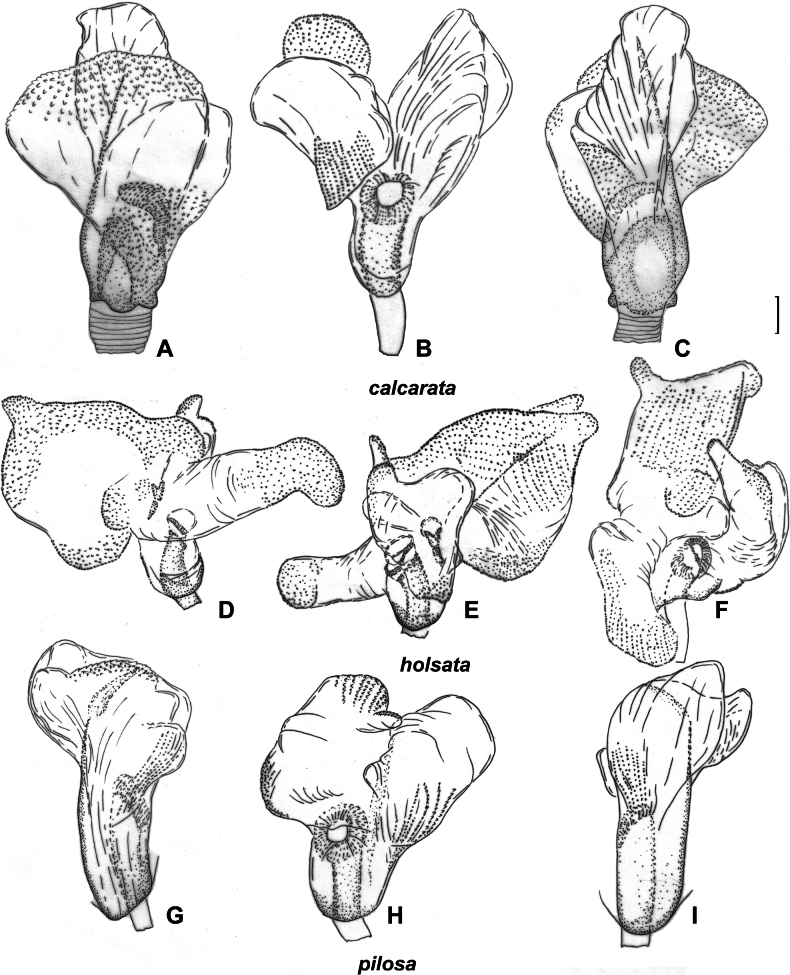
Inflated vesica. *S.calcarata*. ZISP_ENT 00002712 **A** dorsal view **B** left lateral view **C** ventral lateral view. *S.holsata*. ZISP_ENT 00003625 **D** dorsal view **E** ventral **F** left lateral view. *S.pilosa*. ZISP_ENT 00003626 **G** dorsal view **H** left lateral view **I** ventral view.

**Figure 4.**
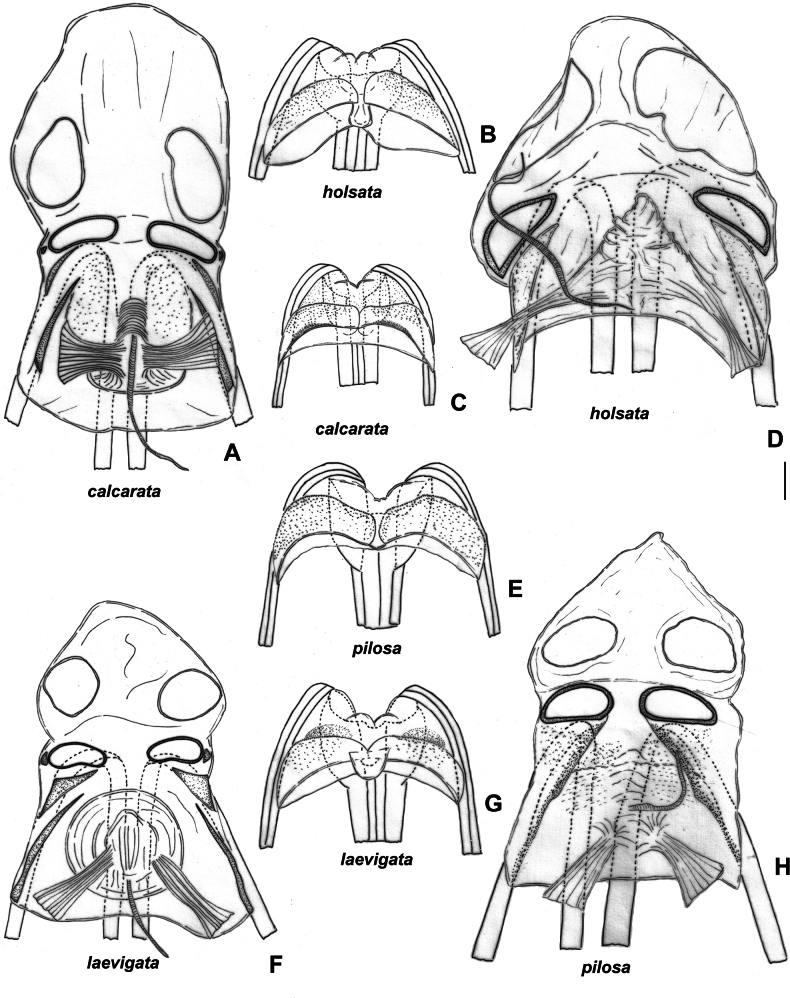
Female genitalia. *S.calcarata*. ZISP_ENT 00002737 **A** dorsal labiate plate **C** posterior wall of bursa copulatrix. *S.holsata*. ZISP_ENT 00003679 **B** posterior wall of bursa copulatrix **D** dorsal labiate plate. *S.pilosa*. ZISP_ENT 00002732 **E** posterior wall of bursa copulatrix **H** dorsal labiate plate. *S.laevigata*. ZISP_ENT 00002738 **F** dorsal labiate plate **G** posterior wall of bursa copulatrix.

**Figure 5.**
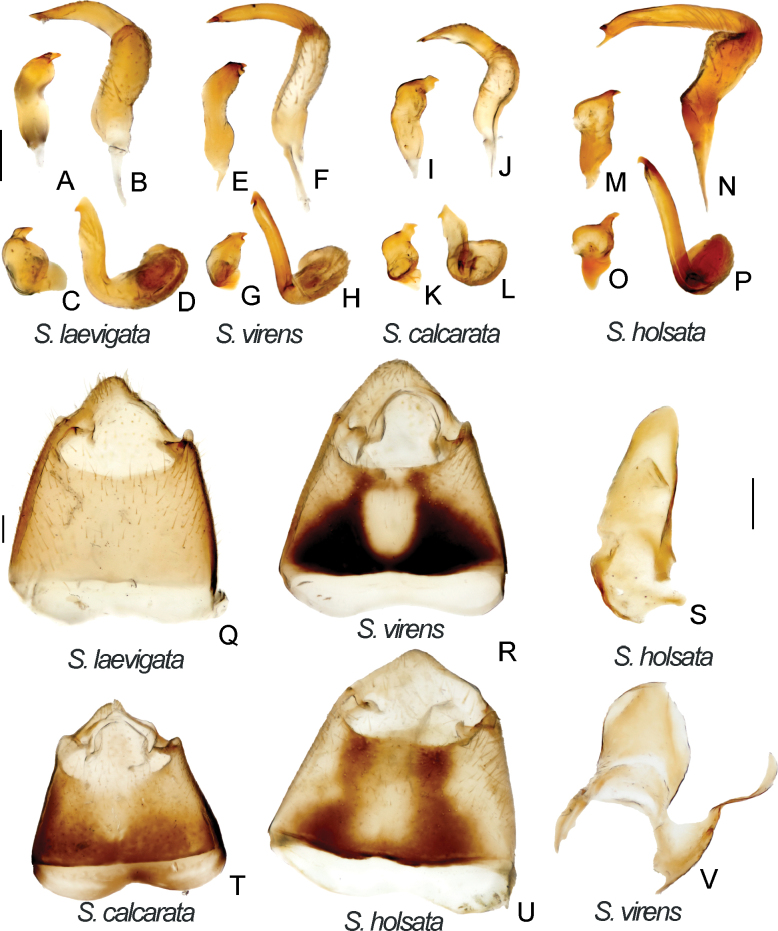
Male genitalia. *S.laevigata*. ZISP_ENT 00002699 **A** right paramere, dorsal view **B** left paramere, dorsal view **C** right paramere, posterior view **D** left paramere. posterior view **Q** genital capsule, dorsal view. *S.virens*. ZISP_ENT 00003616 **E** right paramere, dorsal view **F** left paramere, dorsal view **G** right paramere, posterior view **H** left paramere, posterior view **R** genital capsule **V** theca. *S.calcarata*. ZISP_ENT 00002712 **I** right paramere, dorsal view **J** left paramere, dorsal view **K** right paramere, posterior view **L** left paramere, posterior view **T** genital capsule. *S.holsata*. ZISP_ENT 00003625 **I** right paramere, dorsal view **J** left paramere, dorsal view **K** right paramere, posterior view **L** left paramere, posterior view **S** theca; ZISP_ENT 00002803 **T** genital capsule.

Figs 1E, I, L, N , 2A, G, J , 3A–C , 4A, C, T , 5I–L, T , 9E, F

Miris calcaratus Fallén, 1807: 110 (original description).
Stenodema calcaratum : Reuter 1904: 3 (comb. nov., key to species); Carvalho 1959: 300 (catalogue); Kerzhner and Jaczewski 1964: 958 (key to species); Wagner and Weber 1964: 92 (key to species); Wagner 1974: 110 (key to species).
Stenodema calcarata : Kerzhner 1988: 99 (key to species); Muminov 1989: 126 (key to species); Vinokurov and Kanyukova 1995: 98 (key to species); Kerzhner and Josifov 1999: 191 (catalogue); Yasunaga 2019: 301 (key to species).^1^

##### Diagnosis.

Body length in male 5.9–6.5, in female 5.8–6.7; frons not protruding above clypeus base (Fig. 1I); labium reaching mesosternum but not surpassing it; hind femur with two distinct spines and small tubercle ventroapically, only slightly tapering toward apex (Fig. 2A); hind tibia straight basally (Fig. 2J); swelling above propleura suture straight (Fig. 1I); groove on posterior part of mesopleuron present and distinct (Fig. 1L); paired pits on pronotum between calli present, rounded (Fig. 1E); setae on posterior margin of hind femur as dense as on other parts of femur, distinctly shorter than hind femur width (Fig. 2A); genital capsule only slightly longer than wide, acute apically, with outgrowth near left paramere socket (Fig. 5T); apical half of right paramere as wide as or slightly wider than basal half, bifurcate apically (Fig. 5I, K); left paramere with apical process acute and somewhat elongate in posterior view (Fig. 5L); sensory lobe of left paramere not swollen (Fig. 5J); vesica with three membranous lobes (Fig. 3A–C); dorsal labiate plate ~ 1.5× as long as wide; sclerotized ring ~ 3× as wide as long; distance between sclerotized rings ~ 0.3–0.5× of sclerotized ring width (Fig. 4A); membranous swelling at the middle of dorsal labiate plate absent; posterior wall without dorsal structure between interramal lobes (Fig. 4C).

##### Distribution.

*Stenodemacalcarata* has a trans-Palearctic distribution, ranging from southern and western Europe to the Russian Far East, and extending to Central Asia (Kerzhner and Josifov 1999).

#### ﻿ Stenodema holsata

Taxon classificationAnimaliaHemipteraMiridae

(Fabricius, 1787)

38D1A99E-D75D-55DB-B83B-536A961B62DD

Figs 1D, F , 2H , 3D–F , 4B, D , 5I–L, S, T , 11H, I

Cimex holsatus Fabricius, 1787: 306 (original description).
Stenodema holsatum : Reuter 1904: 6 (comb. nov., key to species); Carvalho 1959: 303 (catalogue); Kerzhner and Jaczewski 1964: 958 (key to species); Wagner and Weber 1964: 97 (key to species); Wagner 1974: 114 (key to species).
Stenodema holsata : Kerzhner 1988: 99 (key to species); Muminov 1989: 128 (key to species); Vinokurov and Kanyukova 1995: 99 (key to species); Kerzhner and Josifov 1999: 194 (catalogue).^2^

##### Diagnosis.

Body length in male 4.7–5.7, in female 5.5–6.4; hemelytron often with brown to dark brown stripe along inner margin; frons not protruding above clypeus base (as in Fig. 1H, I); body length/pronotum width in female 3.9–4.3; antennal segment I in male and female 0.9–1.0× as long as head width; antennal segment I narrower than forefemur; antennal segment II narrower than hind tibia, 4.0–4.4× as long as vertex width; setae on antennal segment I shorter than half of antennal segment I width; labium reaching hind coxa, but not surpassing it; hind femur only slightly tapering toward apex, without spines (Fig. 2H); hind tibia straight basally (as in Fig. 2J); swelling above propleural suture curved (as in Fig. 1H); groove on posterior part of mesopleuron absent (as in Fig. 1M); paired pits on pronotum between calli present, slit-like (Fig. 1F); setae on posterior margin of hind femur as dense as on other parts of femur, distinctly shorter than hind femur width (Fig. 2H); hind femur with distinct markings; genital capsule as wide as long, rounded apically and with swelling near apex, without outgrowths near paramere sockets (Fig. 5U); apical half of right paramere wider than basal part (Fig. 5M); left paramere with elongate thin apical process and with additional outgrowth apically, with sensory lobe swollen (Fig. 5N), apical process rounded apically in posterior view (Fig. 5P); vesica with four membranous lobes (Fig. 3D–F); dorsal labiate plate wider than long; sclerotized ring 2.5–3× as wide as long; distance between sclerotized rings ~ 1.5× longer than sclerotized ring width; membranous swelling at middle of dorsal labiate plate present, triangular (Fig. 4D); posterior wall with dorsal structure between interramal lobes (Fig. 4B).

##### Distribution.

*Stenodemaholsata* has a trans-Palearctic distribution, spanning from southern and western Europe to the Russian Far East, and also known from Central Asia (Kerzhner and Josifov 1999).

##### Notes.

*Stenodemaalgoviensis* and *S.holsata* are two similar species. Wagner (1974) in the key to *Stenodema* species separated those two taxa by the antennal segment I length/head width ratio. However, we found that this ratio is only different in males (1.1 in *S.algoviensis*, 0.9–1.0 in *S.holsata*), which was also previously found by Tamanini (1982). Additionally, males are different in the antennal segment II/vertex width ratio (5.0 in *S.algoviensis*, 4.0–4.4 in *S.holsata*). In terms of genital structure, these two species differ in the shape of the left paramere i.e., *S.holsata* has an additional outgrowth near the apical process, whereas in *S.algoviensis* only a small swelling is present (Wagner 1974: figs 5N, 90E, F; Tamanini 1982: fig. 2A, B, F, G). Vesica and female genitalia of *S.algoviensis*, as well as molecular data, were not studied.

#### ﻿ Stenodema laevigata

Taxon classificationAnimaliaHemipteraMiridae

(Linnaeus, 1758)

6B00D005-81E3-576C-A50D-2AD109A8B2CB

Figs 1H, K, O , 2E , 4F, G , 5A–D, Q , 7A–C , 9G, H

Cimex leavigatus Linnaeus, 1758: 449 (original description).
Stenodema laevigatum : Reuter 1904: 6 (comb. nov., key to species); Carvalho 1959: 304 (catalogue); Kerzhner and Jaczewski 1964: 958 (key to species); Wagner and Weber 1964: 95 (key to species); Wagner 1974: 113 (key to species).
Stenodema laevigata : Muminov 1989: 128 (key to species); Kerzhner and Josifov 1999: 195 (catalogue).^3^

##### Diagnosis.

Body length in male 5.9–6.7, in female 6.8–7.5. Frons not protruding above clypeus base (Fig. 1H); labium reaching metasternum, but not surpassing it (Fig. 1O); hind femur distinctly tapering towards apex, without spines (Fig. 2E); hind tibia curved basally (as in Fig. 2E); swelling above propleural suture curved (Fig. 1H); groove on posterior part of mesopleuron present, shallow (Fig. 1K); paired pits on pronotum between calli absent (as in Fig. 1G); setae on posterior margin of hind femur denser than on other parts of femur, distinctly shorter than hind femur width (Fig. 2E); genital capsule slightly longer than wide, acute apically, with outgrowth near each paramere socket (Fig. 5Q); apical half of right paramere as wide as basal half (Fig. 5A); apical process of right paramere more or less acute apically in posterior view but not elongate (Fig. 5D); sensory lobe of left paramere swollen (Fig. 5B); vesica with two membranous lobes (Fig. 7A–C); dorsal labiate plate as long as wide, sclerotized ring 2–2.5× as long as wide; distance between sclerotized rings ~ 0.5–0.75× as long as sclerotized ring width; membranous swelling on dorsal labiate plate present, rounded, not reaching sclerotized ring (Fig. 4F); posterior wall with dorsal structure between interramal lobes (Fig. 5G).

##### Distribution.

*Stenodemalaevigata* is mostly known from Western Palearctic, and there are no records from Siberia. However, the species was recorded from Kyrgyzstan and China (Kerzhner and Josifov 1999).

#### ﻿ Stenodema pilosa

Taxon classificationAnimaliaHemipteraMiridae

(Jakovlev, 1889)

F1683262-14FF-59D3-8C03-C92125F9C299

**Figure 6.**
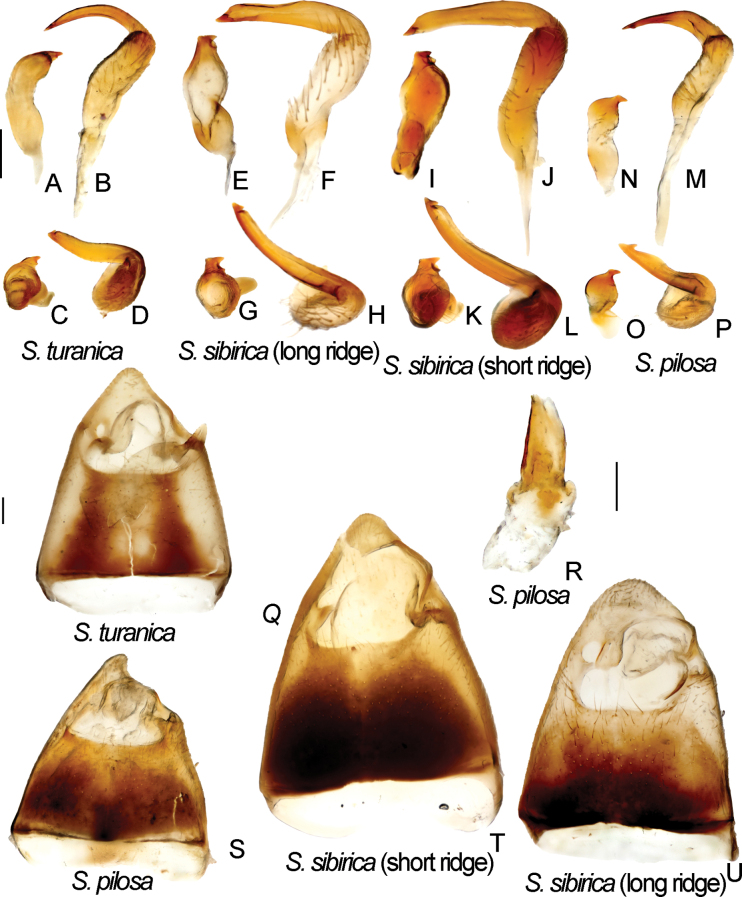
Male genitalia. *S.turanica*. ZISP_ENT 00003654 **A** right paramere, dorsal view **C** right paramere, posterior view **Q** genital capsule, dorsal view; ZISP_ENT 00003618 **B** left paramere, dorsal view **D** left paramere, posterior view. *S.sibirica*. ZISP_ENT 00003617 (vesica with long ridge) **E** right paramere, dorsal view **F** left paramere, dorsal view **G** right paramere, posterior view **H** left paramere, posterior view **U** genital capsule, dorsal view; ZISP_ENT 00003620 (vesica with short ridge) **I** right paramere, dorsal view **J** left paramere, dorsal view **K** right paramere, posterior view **L** left paramere, posterior view **T** genital capsule, dorsal view. *S.pilosa*. ZISP_ENT 00003626 **N** right paramere, dorsal view **M** left paramere, dorsal view **O** right paramere, posterior view **P** left paramere, posterior view **R** theca **S** genital capsule, dorsal view.

Figs 1A, G, Q , 2D , 3G–I , 4E, H , 6N–P, R, S , 9A–D

Brachytropis pilosa Jakovlev, 1889: 243 (original description).
Stenodema pilosum : Reuter 1904: 3 (comb. nov., key to species).
Stenodema pilosa : Muminov 1989: 127 (key to species).
Stenodema trispinosum Reuter, 1904: 8 (original description); Carvalho 1959: 301 (catalogue); Wagner and Weber 1964: 93 (key to species); Kerzhner and Jaczewski 1964: 958 (key to species); Wagner 1974: 110 (key to species). New synonym.
Stenodema trispinosa : Kerzhner 1988: 99 (key to species); Muminov 1989: 126 (key to species); Vinokurov and Kanyukova 1995: 98 (key to species); Kerzhner and Josifov 1999: 191 (catalogue); Yasunaga 2019: 301 (key to species).^4^

##### Type material examined.

***Lectotype*** of *Brachytropispilosa* Jakovlev, 1889: China • ♀; Xinjang: Quiemo [oasis Tschertschen]; 38.14°N, 85.53°E; 11 Jun 1885; NM Przhevalsky; (ZISP_ENT 00015588); (ZISP).

***Lectotype*** of *Stenodematrispinosum* Reuter, 1904: Russia: • ♀; Yakutia Rep., Batylim, Lena River; 62.02°N, 129.73°E; 18–19 Jul 1901; B. Poppius; (http://id.luomus.fi/GZ.56520); (MZH).

***Paralectotypes*** of *Stenodematrispinosum* Reuter, 1904: Russia • ♀; Arkhangelsk Prov.: Solovetsky Islands; 65.08°N, 35.88°E; no date provided; Levander; (http://id.luomus.fi/GZ.25545); (MZH) • 3♀; Buryatia Rep.: Dauria; 53°N, 115°E; 1842; R.F. Sahlberg; (http://id.luomus.fi/GZ.56517, http://id.luomus.fi/GZ.56518, http://id.luomus.fi/GZ.56519); (MZH) • ♀; Khakassia Rep.: Sayanogorsk [Osnatjennaja]; 53.09°N, 91.40°E; 1885; R.E. Hammarström; (http://id.luomus.fi/GZ.56523); (MZH) • ♀; Khanty-Mansi Autonomous Okrug: Leushi [Leusch]; 56.62°N, 65.72°E; no date provided; N. Sundman; (http://id.luomus.fi/GZ.56516); (MZH) • ♀; Yakutia Rep.: Olekminsk; 60.37°N, 120.43°E; 1901; B. Poppius; (http://id.luomus.fi/GZ.56521); (MZH) • ♀; Ust-Aldan 63.52°N, 129.41°E; 13 Jul 1901; B. Poppius; (http://id.luomus.fi/GZ.56524); (MZH) • ♀; Yakutsk, 62.03°N, 129.73°E; 1901; B. Poppius; (http://id.luomus.fi/GZ.56522); (MZH).

##### Diagnosis.

Body length in male 5.4–6.4, in female 6.0–6.3; frons not protruding above clypeus base (as in Fig. I); labium reaching middle coxa but not surpassing it; hind femur only slightly tapering toward apex, with three spines ventroapically; setae on posterior margin of hind femur as dense as on other parts of femur, distinctly shorter than hind femur width (Fig. 2D); hind tibia straight basally (as in Fig. 2G); swelling above propleura suture straight (as in Fig. 1I); groove on posterior part of mesopleuron absent (as in Fig. 1M); paired pits between calli small, not discernible from punctures or absent (Fig. 1G); genital capsule slightly longer than wide; apex of genital capsule acute and curved left; left paramere socket with outgrowth (Fig. 6S); apical half of right paramere as wide as basal half, not bifurcate apically (Fig. 6N, O); left paramere with apical process acute and elongate in posterior view (Fig. 6P) and with swollen sensory lobe (Fig. 6M); vesica with two membranous lobes (Fig. 3G–I); dorsal labiate plate ~ 1.5× as long as wide; sclerotized ring ~ 1.5× as wide as long; distance between sclerotized rings ~ 0.3–0.4× as long as sclerotized ring width; membranous swelling on dorsal labiate plate absent (Fig. 4H); posterior wall without dorsal structure between interramal lobes (Fig. 4E).

##### Distribution.

In its currently accepted concept, *S.pilosa* is a Holarctic species with a wide circumpolar distribution. It extends south to California, New Mexico, Texas, and Georgia in the Nearctic, and to France, Romania, Turkey, Transcaucasia, Central Asia, Central China, and Korea in the Palearctic (Wheeler and Henry 1992; Kerzhner and Josifov 1999). Based on the distribution pattern, *S.trispinosa*, here synonymized with *S.pilosa*, is considered a true Holarctic species, with possible post-Pleistocene expansion from the Beringia refugium (Lattin and Oman 1983; Wheeler and Henry 1992).

##### Notes.

*Stenodemapilosa* was initially described within the genus *Brachytropis* Fieber, 1858 (Jakovlev 1889), an unnecessary new name for Brachystira Fieber, 1858, currently recognized as a subgenus of Stenodema (Reuter 1904). In the original description Jakovlev (1889) mentioned that this species had two spines on the hind femur. Reuter (1904) described *Stenodematrispinosa* as a distinctive species with three spines on the hind femur. He included *S.pilosa* in his key to species based solely on the original description, noting that he had not personally examined specimens of this species. Muminov (1989) designated the lectotype of *B.pilosa* and mentioned that it had three spines on the hind femur, and that *S.pilosa* and *S.trispinosa* did not have any differences in the male genitalia structures. He hypothesized that Jakovlev (1889) indicated the presence of two spines on the hind femur in *B.pilosa* due to the relatively small size of the basal one. However, he followed Reuter’s key in other respects and differentiated these two species by the length of antennal segment I, although exact measurements or ratios were not provided, and by the length of setae on this segment and hind tibiae.

We examined the lectotypes of both species as well as other specimens authentically identified as *S.pilosa*, and did not find any characters separating this species from *S.trispinosa*. Most probably, *S.trispinosa* was treated as a separate new species by Reuter (1904), because of the mistake in the description of *S.pilosa*. According to our measurements, *S.pilosa* and *S.trispinosa* do not differ in the antennal segment II length and we could not find any differences in the setae on the hind tibia. We fully concur with Muminov (1989) regarding the lack of differences in the male genitalia structure, and we were unable to identify any distinctions in the female genitalia either. Therefore, we synonymize *S.trispinosa* Reuter, 1904 with *S.pilosa* (Jakovlev, 1889).

#### ﻿ Stenodema sibirica

Taxon classificationAnimaliaHemipteraMiridae

Bergroth, 1914

8C5152D7-023D-578A-80D9-CFF0729FA029

Figs 1M , 2F , 6E–H, I, U , 10B, F , 12E–G , 13

Miris virens lateralis Sahlberg, 1873: 23 (original description).
Stenodema lateralis : Reuter 1891: 187 (comb. nov.).
Stenodema sibiricum Bergroth, 1914: 183 (new name for junior secondary homonym of Stenodemalateralis (Geoffroy, 1785)); Carvalho 1959: 306 (catalogue).
Stenodema sibirica ; Kerzhner 1988: 99 (key to species); Muminov 1989: 127 (key to species); Vinokurov and Kanyukova 1995: 98 (key to species); Kerzhner and Josifov 1999: 196 (catalogue); Yasunaga 2019: 301 (key to species).^5^

##### Type material examined.

***Lectotype*** of *Mirisvirenslateralis* Sahlberg, 1873: Russia • ♀; Krasnoyarsk Terr., Yeniseysk [Jeniseisk]; 58.45°N, 92.18°E; no date provided; J. Sahlberg; (http://id.luomus.fi/GZ.56515); (MZH).

##### Diagnosis.

Body length in male 5.8–6.5, in female 6.2–6.8; frons protruding above clypeus base (as in Fig. 1H, I); setae on hemelytron simple; hemelytron brown to dark brown medially and yellow to pale brown along outer margin (Fig. 12E–G); male vertex width/eye ratio 2.1–2.4; labium reaching mesocoxa but not surpassing it (as in Fig. 1N); hind femur only slightly tapering towards apex, without spines; setae on posterior margin of hind femur as dense as on other parts of femur, shorter than half of hind femur (Fig. 2F); hind tibia not curved basally (as in Fig. 2J); swelling on propleura curved (Fig. 1H); antennal segment I length/head width ratio in male 1.0, in female 0.9–1.0; antennal segment I /pronotum lengths ratio 0.8–0.9 in male, 0.8 in female; antennal segment I as wide as or slightly narrower than eye diameter; groove on posterior part of mesopleuron absent (Fig. 1M); paired pits between calli absent (as in Fig. 1G), setae on antennal segment I shorter than antennal segment I width; genital capsule ~ 1.5× as long as wide, more or less acute apically, with outgrowth near left paramere socket (Fig. 6T, U); right paramere ca 3× as long as wide, its apical part slightly wider than basal part, its apical process bifurcate, ca 0.1× as long as rest of paramere (Fig. 6E, I); left paramere with apical process acute at posterior view (Fig. 6K, P), its sensory lobe swollen (Fig. 6J, M); vesica with one large and two small membranous lobes (Fig. 13); dorsal labiate plate slightly longer than wide; sclerotized ring ~ 3× as wide as long; distance between sclerotized rings ~ 0.3–0.5× as long as sclerotized ring width (Fig. 10B); posterior wall with sigmoid process between interramal lobes (Fig. 10F).

##### Distribution.

*Stenodemasibirica* is known from Siberia, northern China, Mongolia, the Russian Far East, and Korea (Kerzhner and Josifov 1999).

##### Notes.

Among the material preserved at ZISP, we found specimens with two types of vesica. They differ in the shape of the membranous lobes and the length of the ridge with sclerotized teeth (cf. Fig. 13A–C and Fig. 13D–F). The genital capsule and parameres of specimens with these two types of vesica were very similar (cf. Fig. 6E–H, U and Fig. 6I–L, T). We found only two males with the short, sclerotized ridge, and there were no females from the same series. There were no differences in the habitus between the specimens with two types of male genitalia. The lectotype preserved at the Finnish Museum of Natural History is a female, and we refrained from dissecting its genitalia, as it will not provide us with additional information on the issue. Therefore, we treat widespread form as *S.sibirica* and refrain from making any taxonomic decisions on the two specimens with another type of vesica, as the corresponding species may have been already described from China (see below for comparisons).

*Stenodemasibirica* is very similar to *S.rubrinervis* Horváth, 1905. They have minor differences in the measurements i.e., vertex width/eye diameter ratio in male (2.1–2.4 in *S.sibirica* and 1.7–2.0 in *S.rubrinervis*) and length of antennal segment I (1.8–2.1 in *S.sibirica* and 2.5–3.0 in *S.rubrinervis*) (Table 1). The genitalia of those two species are very similar, and vesica of *S.rubrinervis* also has a long ridge of sclerotized teeth (Yasunaga 2019: fig. 8C).

#### ﻿ Stenodema turanica

Taxon classificationAnimaliaHemipteraMiridae

Reuter, 1904

5B686FFC-4F89-5E3C-AC84-53FADD6652C5

**Figure 7.**
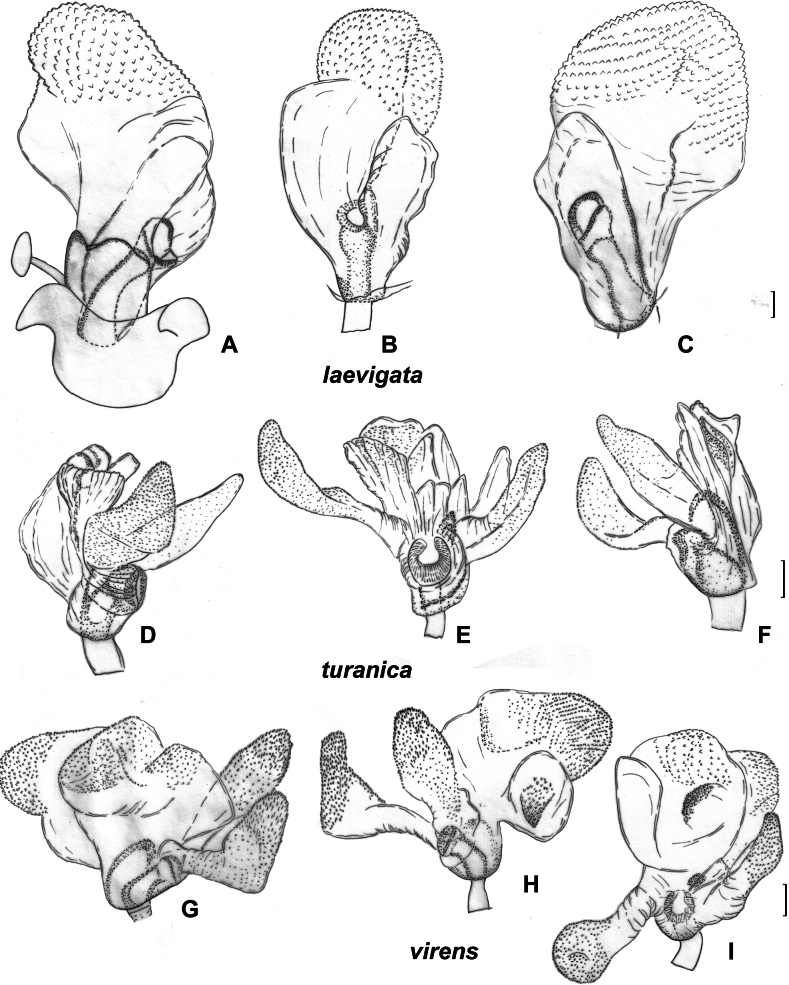
Inflated vesica, *S.laevigata*, ZISP_ENT 00002699 **A** dorsal view **B** left lateral view **C** ventral lateral view. *S.turanica*, ZISP_ENT 00003618 **D** dorsal view **E** left lateral view **F** ventral lateral view. *S.virens*, ZISP_ENT 00003616 **G** dorsal view **H** ventral view **I** left lateral view.

**Figure 8.**
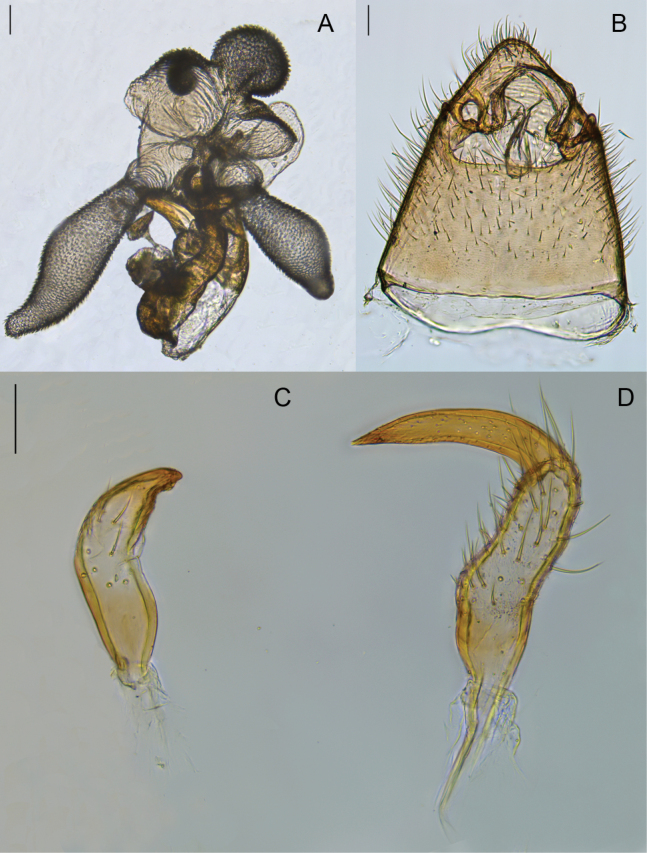
Male genitalia of *Brachytropisturanica*. lectotype **A** inflated aedeagus. left lateral view **B** genital capsule **C** right paramere. dorsal view **D** left paramere. dorsal view.

Figs 1B, C , 2C, I , 6A–D, Q , 7D–F , 8 , 10A, E , 11A–D

Stenodema turanicum Reuter, 1904: 23 (original description); Carvalho 1959: 307 (catalogue); Wagner 1974: 112 (key to species).
Stenodema turanica : Muminov 1989: 127 (key to species); Kerzhner and Josifov 1999: 196 (catalogue).^6^

##### Type material examined.

***Lectotype*** of *Stenodematuranicum* Reuter, 1904 (designated here): Turkmenistan • ♂; Kopet Dagh; 38.06°N, 57.37°E; no date provided; K.O. Ahnger; (http://id.luomus.fi/GZ.56573); (MZH).

***Paralectotypes*** of *Stenodematuranicum* Reuter, 1904: Kyrgyzstan • 2♀; Chiburgan [Tschiburgan] valley; 39.60°N, 70.65°E; no date provided; A.P. Fedchenko; (http://id.luomus.fi/GZ.56577, http://id.luomus.fi/GZ.56580); (MZH) • ♀; Gulcha [Gulscha]; 40.31°N, 73.44°E; no date provided; A.P. Fedchenko; (http://id.luomus.fi/GZ.56575); (MZH) Tajikistan: • ♂ Panjakent [Pendzhikent], valley of Zeravshan River; 39.48°N, 67.60°E; no date provided; A.P. Fedchenko; (AMNH_PBI 00345037, http://id.luomus.fi/GZ.56652); • 2♀; (AMNH_PBI 00345035, http://id.luomus.fi/GZ.56650; AMNH_PBI 00345036, http://id.luomus.fi/GZ.56651); (MZH). Turkmenistan: • ♂; Kopet Dagh; 38.06°N, 57.37°E; no date provided; K.O. Ahnger; (http://id.luomus.fi/GZ.56579); • 2♀ (http://id.luomus.fi/GZ.56578, http://id.luomus.fi/GZ.56572); (MZH) • ♀ Gokdepe [Geok-tepe]; 38.15°N, 57.95°E; K.O. Ahnger; (http://id.luomus.fi/GZ.56574); (MZH). Uzbekistan: • ♀; Shohimardon [Schagimardan]; 39.99°N, 71.81°E; no date provided; A.P. Fedchenko; (http://id.luomus.fi/GZ.56576); (MZH).

##### Diagnosis.

Body length in male 6.1–6.8, in female 6.6–7.3; frons protruding above clypeus base (Fig. 1H, I); labium reaching middle coxa (as in Fig. 1N); hind femur distinctly tapering towards apex, without spines, not enlarged, 6–8× as long as wide (Fig. 2C); hind tibia curved basally (Fig. 2I); swelling on propleura curved (Fig. 1H); antennal segment I length/head width ratio in male 1.0, in female 0.9–1.0; antennal segment I length/pronotum length ratio 0.7–0.9 in male, 0.7 in female; antennal segment I not widened basally, its setae at base as dense as on other parts of this segment; setae of antennal segment I simple; antennal segment II length/head width ratio in male 3.1–3.5; groove on posterior part of mesopleuron absent (as in Fig. 1M); paired pits between calli absent (as in Fig. 1G); setae on posterior margin of hind femur denser than on other parts of femur, shorter than half of hind femur width (Fig. 2C); genital capsule only slightly longer than wide, acute apically, with outgrowth near left paramere socket (Fig. 6Q); right paramere ca 3× as long as wide, its apical part as wide as basal part, apical process not bifurcate (Fig. 6A); left paramere with apical process acute in posterior view (Fig. 6D), its sensory lobe swollen (Fig. 6B); vesica with four membranous lobes (Figs 7E, F, 8A); dorsal labiate plate as long as wide, sclerotized ring 2–3× as long as wide; distance between sclerotized rings 4× as long as sclerotized ring width; membranous swelling on dorsal labiate plate not covering sclerotized rings (Fig. 10A); posterior wall with dorsal structure and sigmoid process between interramal lobes, dorsal structure oval (Fig. 10E).

##### Distribution.

*Stenodematuranica* is known from the Balkans, Caucasus, Turkey, Iraq, Iran, Central Asia, Mongolia, and northwestern China (Kerzhner and Josifov 1999).

##### Notes.

*Stenodematuranica* was originally described (Reuter 1904) from the type series collected by K.O. Ahnger and A.P. Fedchenko in Central Asia and retained at the Finnish Museum of Natural History (MZH). Due to the observed similarity of *S.turanica* with *S.virens*, here we designated the lectotype for *Stenodematuranicum* Reuter, 1904, the male from Kopet Dagh mountains in Turkmenistan (Fig. 8, http://id.luomus.fi/GZ.56573).

*Stenodematuranica* and *S.virens* are very similar externally. According to Wagner (1974), in *S.turanica* antennal segment II is twice as long as segments III and IV combined, whereas in *S.virens* this segment is only 1.5× times as long as segments III and IV combined. Additionally, the setae on the inner margin of hind femur are inclined in *S.virens*, whereas they are straight in *S.turanica*. The setae on the hind femur are more or less the same in both species (Fig. 2B, C). We confirm that the antennal segment II is longer in males of *S.turanica* rather than in males of *S.virens*, in particular, antennal segment II/head width ratio is 3.1–3.5 in *S.turanica* and 2.4–2.6 in *S.virens*. However, we were unable to find reliable differences in female measurements. These two species differ from each other in both, male (compare Fig. 7D–F and Fig. 7G–I) and female (compare Fig. 10A, E and Fig. 10C, D) genitalia.

#### ﻿ Stenodema virens

Taxon classificationAnimaliaHemipteraMiridae

(Linnaeus, 1767)

F03A6315-1EBE-5BD0-AD9F-2283DEBBE124

Figs 1J, P , 2B , 5E–H, R, V , 7G–I , 10C, D , 11E–G

Cimex virens Linnaeus, 1767: 730 (original description).
Stenodema virens Reuter, 1904: 4 (comb. nov., key to species); Carvalho 1959: 307 (catalogue); Kerzhner and Jaczewski 1964: 958 (key to species); Wagner and Weber 1964: 94 (key to species); Wagner 1974: 112 (key to species); Muminov 1989: 127 (key to species); Vinokurov and Kanyukova 1995: 98 (key to species); Kerzhner and Josifov 1999: 196 (catalogue).^7^

##### Diagnosis.

Body length in male 6.0–6.6, in female 6.1–7.1; frons protruding above clypeus base (as in Fig. 1C); labium reaching middle coxa, but not surpassing it (as in Fig. 1N); hind femur distinctly tapering towards apex, without spines (Fig. 2B), 6–8× as long as wide; hind tibia curved basally (as in Fig. 2I); swelling on propleura curved (as in Fig. 1H); antennal segment I length/head width ratio in male 1.0, in female 0.8–1.0; antennal segment I/pronotum length ratio 0.6–0.7 in male, 0.6–0.8 in female; antennal segment I not widened basally, its setae at base as dense as on other parts of this segment; setae in antennal segment I simple; antennal segment II length/head width ratio in male 2.4–2.6; groove on posterior part of mesopleuron absent (as in Fig. 1M); paired pits between calli absent (as in Fig. 1G); setae on posterior margin of hind femur denser than on other parts of femur, shorter than half of hind femur width (Fig. 2B); genital capsule only slightly longer than wide, acute apically, with outgrowth near left paramere socket (Fig. 5R); right paramere ~ 4× as long as wide, its apical part as wide as basal part, apical process bifurcate (Fig. 5E, G); right paramere with apical process acute in posterior view (Fig. 5Р), its sensory lobe swollen (Fig. 5F); vesica with four membranous lobes (Fig. 7G–I); membranous swelling on dorsal labiate plate not covering sclerotized rings (Fig. 10C); posterior wall with dorsal structure and sigmoid process between interramal lobes, dorsal structure rounded (Fig. 10D).

**Figure 9.**
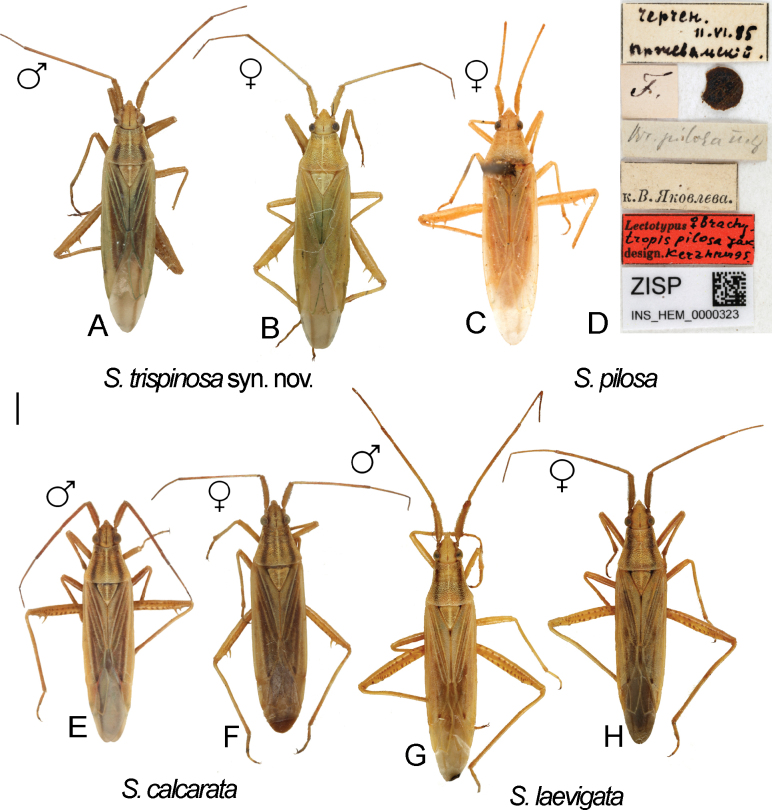
Digital images of habitus. *S.pilosa*. specimens previously identified as *S.trispinosa***A** ♂ ZISP_ENT 00004882 **B** ♀ ZISP_ENT 00004886 **C** Lectotype of *Brachytropispilosa***D** labels attached to the lectotype. *S.calcarata***E** ♂ ZISP_ENT 00004876 **F** ♀ ZISP_ENT 00004864. *S.laevigata***G** ♂ ZISP_ENT 00004921 **H** ♀ ZISP_ENT 00004923.

**Figure 10.**
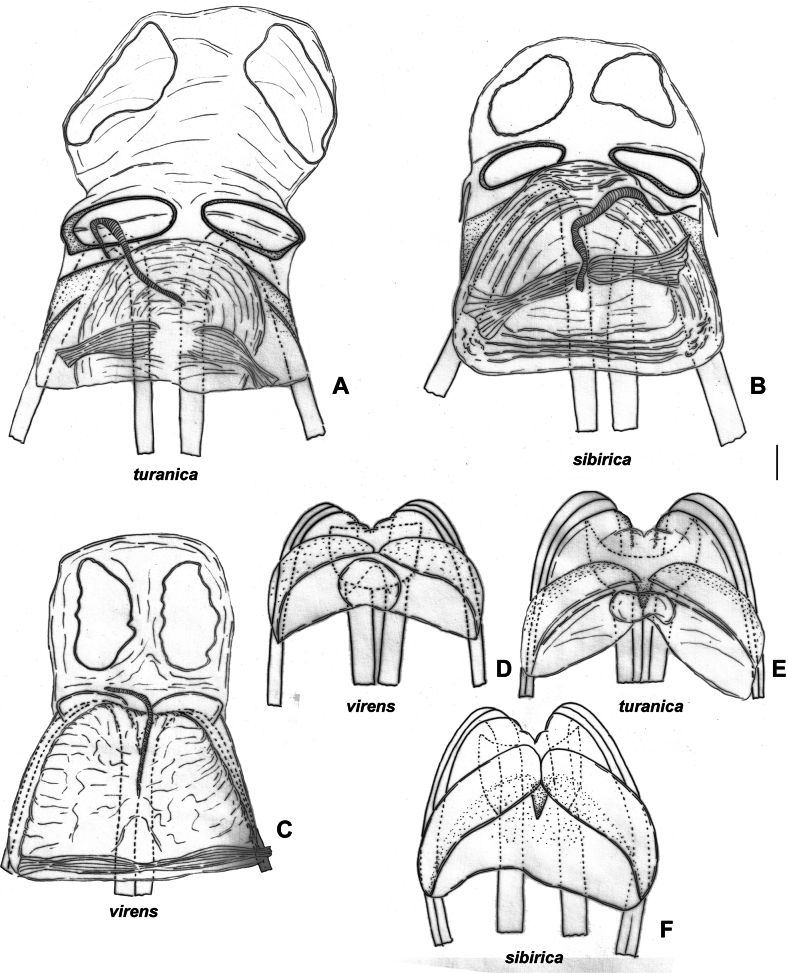
Female genitalia. *S.turanica*. ZISP_ENT 00002735 **A** dorsal labiate plate **E** posterior wall of bursa copulatrix. *S.sibirica*. ZISP_ENT 00003679 **B** posterior wall of bursa copulatrix **F** dorsal labiate plate. *S.virens* ZISP_ENT 00002732 **C** posterior wall of bursa copulatrix **D** dorsal labiate plate.

##### Distribution.

*Stenodemavirens* is widely distributed in Europe, the Near East, and the Caucasus, extending eastwards to Yakutia, Buryatia, Mongolia, and northern China (Kerzhner and Josifov 1999).

**Figure 11.**
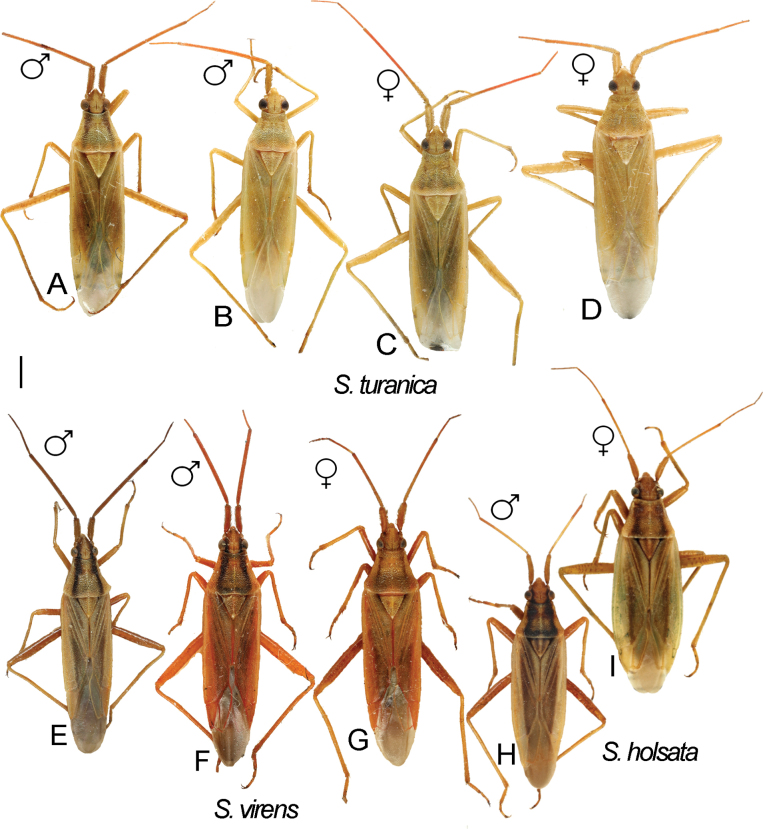
Digital images of habitus. *S.turanica***A** ♂ ZISP_ENT 00004938 **B** ♂ ZISP_ENT 00004937 **C** ♀. ZISP_ENT 00004935 **D** ♀. ZISP_ENT 00004953. *S.virens***E** ♂ ZISP_ENT 00004898 **F** ♂ ZISP_ENT 00004897 **G** ♀. ZISP_ENT 00004894. *S.holsata***H** ♂ ZISP_ENT 00004903 **I** ♀ ZISP_ENT 00004907.

**Figure 12.**
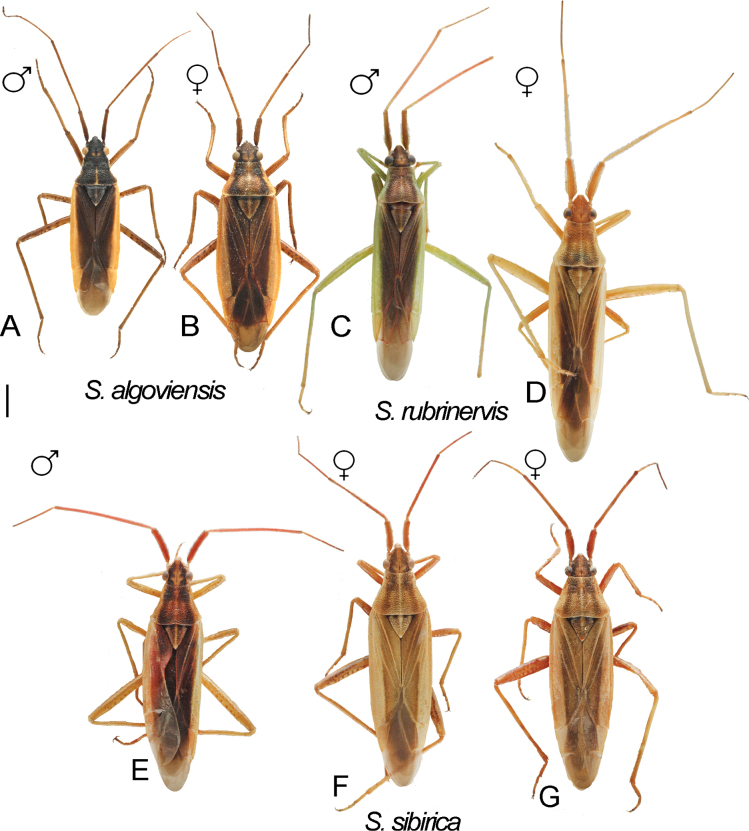
Digital images of habitus. *S.algoviensis***A** ♂ ZISP_ENT 00004951 **B** ♀ ZISP_ENT 00004950. *S.rubrinervis***C** ♂ ZISP_ENT 00004941 **D** ♀ ZISP_ENT 00004960. *S.sibirica***E** ♂ ZISP_ENT 00004919 **F** ♀ ZISP_ENT 00004928 **G** ♀ ZISP_ENT 00004929.

**Figure 13.**
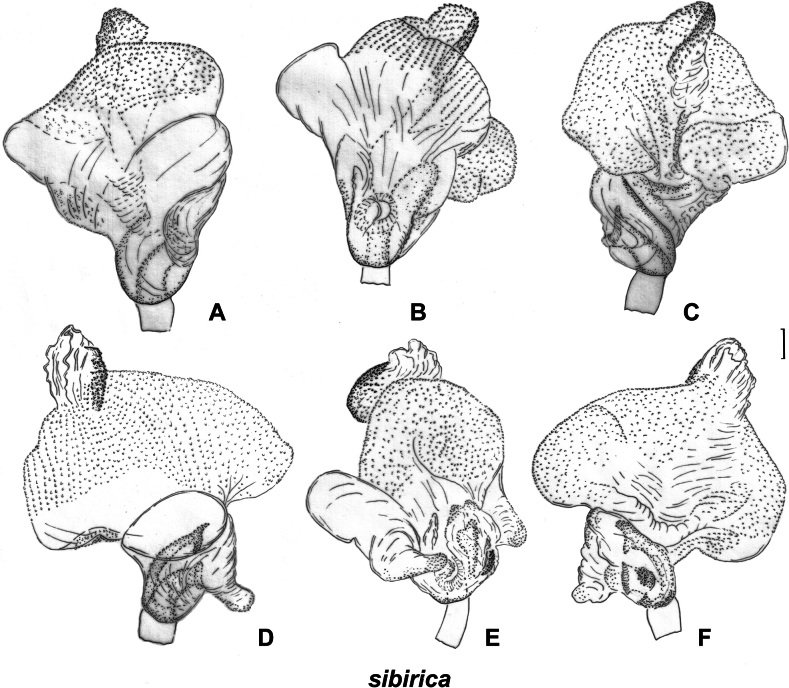
Inflated vesica. *S.sibirica* vesica with long ridge ZISP_ENT 00003617 **A** dorsal view **B** left lateral view **C** ventral lateral view; vesica with short ridge ZISP_ENT 00003620 **D** dorsal view **E** left lateral view **F** ventral lateral view.

**Figure 14.**
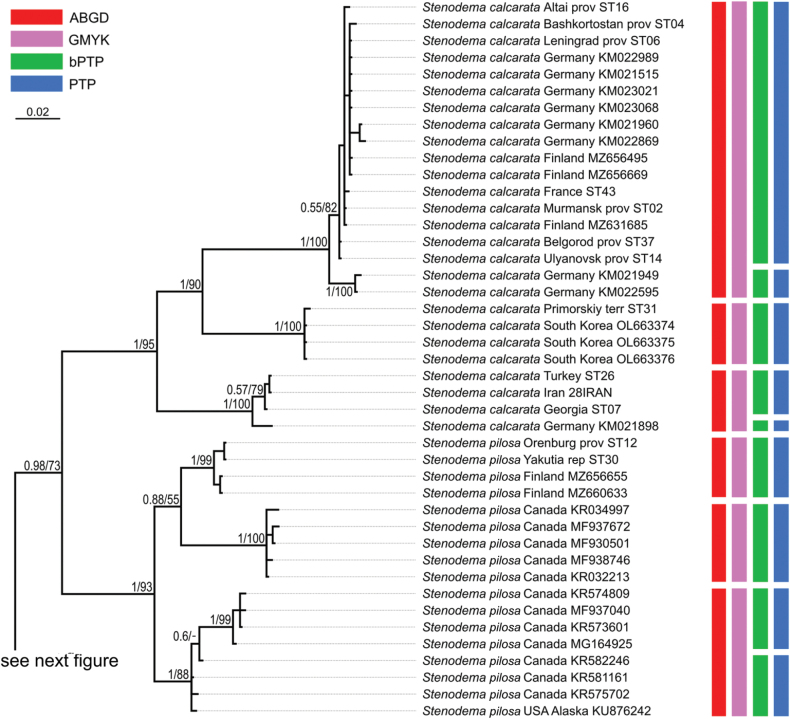
Phylogeny obtained using the Bayesian inference based on the COI dataset, part 1. The supports are provided above the branches. The numbers on the left correspond to PP, the numbers on the right correspond to BS obtained with RAxML. The color stripes correspond to the results of the species delimitation analyses in the following order: ABGD, GMYC, bPTP, PTP.

**Figure 15.**
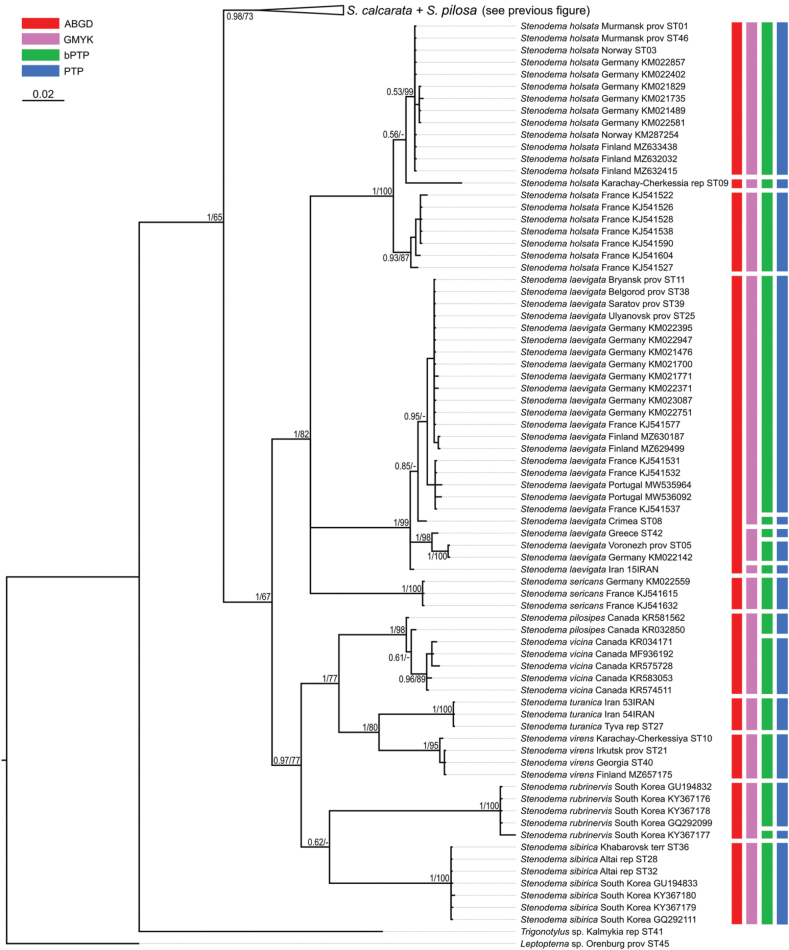
Phylogeny obtained using the Bayesian inference based on the COI dataset, part 2. The supports are provided above the branches. The numbers on the left correspond to PP, the numbers on the right correspond to BS obtained with RAxML. The color stripes correspond to the results of the species delimitation analyses in the following order: ABGD, GMYC, bPTP, PTP.

**Figure 16.**
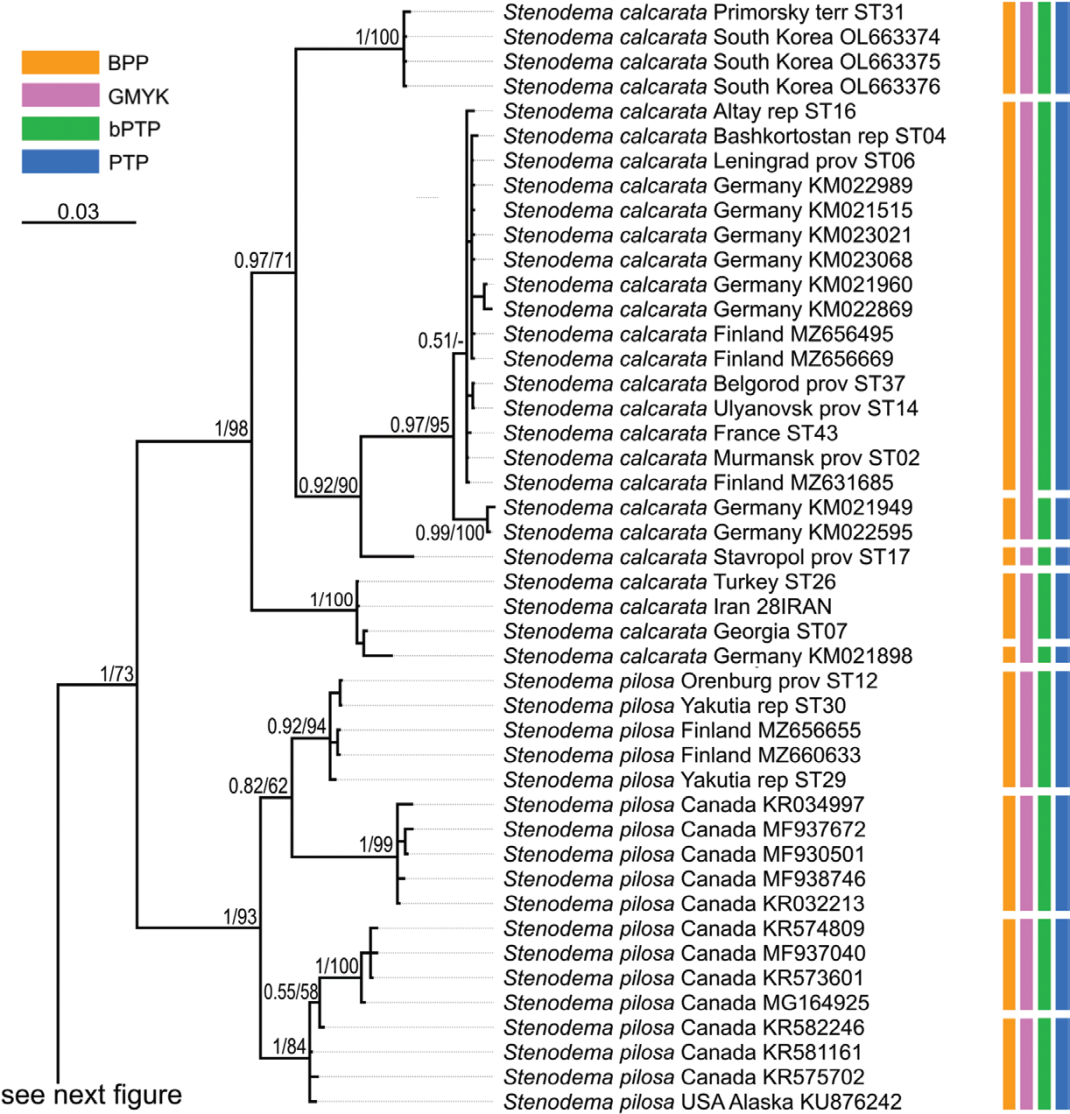
Phylogeny obtained using the Bayesian inference based on the full dataset dataset, part 1. The supports are provided above the branches. The numbers on the left correspond to PP, the numbers on the right correspond to BS obtained with RAxML. The color stripes correspond to the results of the species delimitation analyses in the following order: BPP, GMYC, bPTP, PTP.

**Figure 17.**
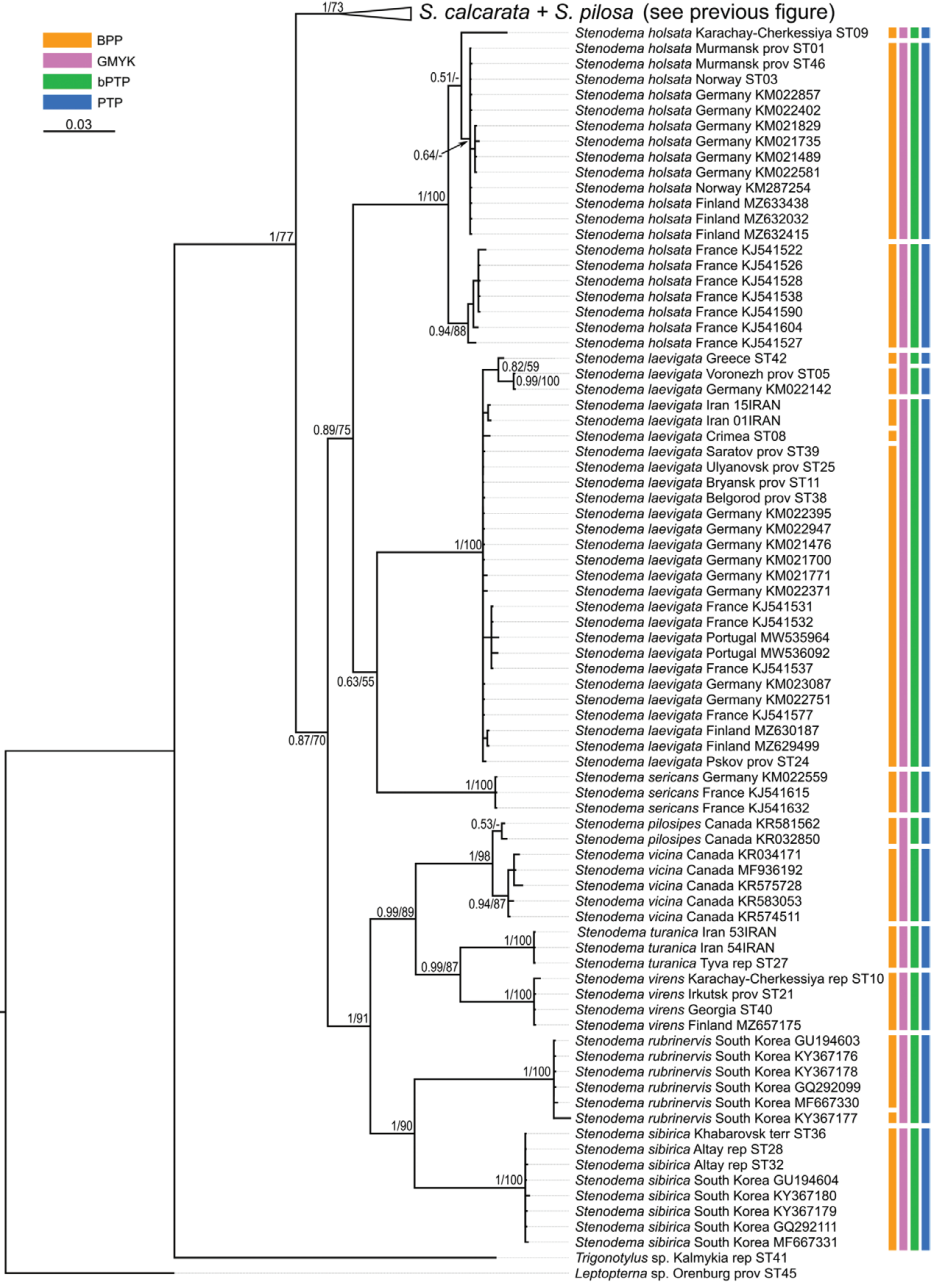
Phylogeny obtained using the Bayesian inference based on the full dataset, part 2. The supports are provided above the branches. The numbers on the left correspond to PP, the numbers on the right correspond to BS obtained with RAxML. The color stripes correspond to the results of the species delimitation analyses in the following order: BPP, GMYC, bPTP, PTP.

**Figure 18.**
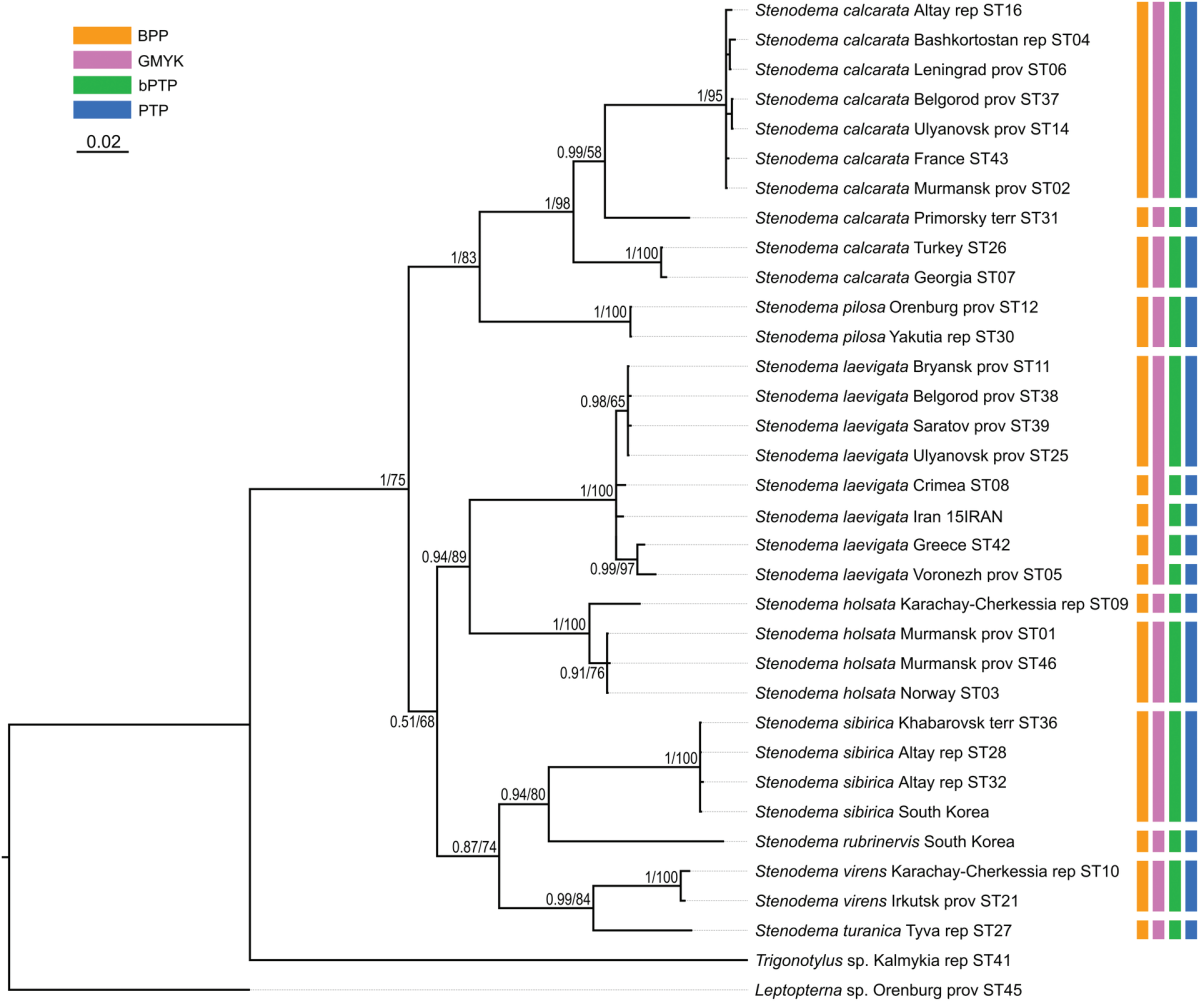
Phylogeny obtained using the Bayesian inference based on the reduced dataset. The supports are provided above the branches. The numbers on the left correspond to PP, the numbers on the right correspond to BS obtained with RAxML. The color stripes correspond to the results of the species delimitation analyses in the following order: BPP, GMYC, bPTP, PTP.

**Figure 19.**
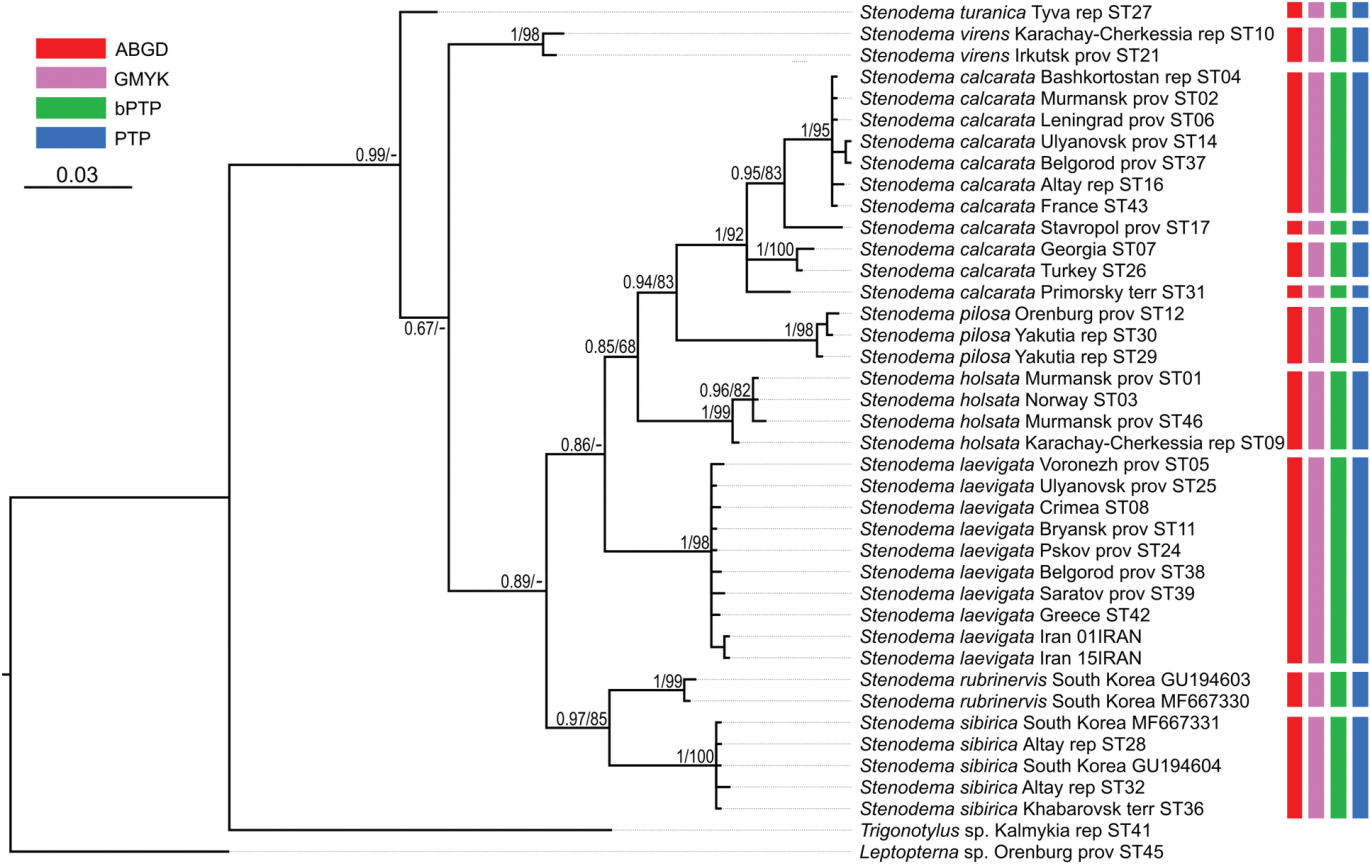
Phylogeny obtained using the Bayesian inference based on the 16S rRNA dataset. The supports are provided above the branches. The numbers on the left correspond to PP, the number on the right correspond to BS obtained with RAxML. The color stripes correspond to the results of the species delimitation analyses in the following order: ABGD, GMYC, bPTP, PTP.

### ﻿Morphological taxonomy

Based on the descriptions and material examined, we could delimit five morphological groups within *Stenodema*.

1. *S.calcarata*- *pilosa* group (subgenus *Brachystira*). This group has the frons not protruding above the clypeus (Fig. 1I) and hind femur possessing ventroapical spines and not tapering towards apex (Fig. 2A, D). The information on *S.falki* is very scarce, but this species might also belong to this group. According to Kelton (1961), the Nearctic species *S.falki* is very similar to *S.pilosa*, but differs in body ratios and male genitalia, although Schwartz (1987) suspected that these species might be synonymous. Among the species with the female genitalia examined, *S.calcarata* and *S.pilosa* are similar in the absence of the membranous swelling on the dorsal labiate plate and the absence of the dorsal structure between interramal lobes (Fig. 4A, C, E, H).
2. *S.holsata* group includes species with the frons not protruding above the clypeus (Fig. 1H) and hind femur without spines, and non-tapered apical region (Fig. 2E, H). *Stenodemaalgoviensis*, *S.chinensis*, *S.holsata*, *S.plebeja*, and *S.sericans* possess this set of characters. *Stenodemachinensis* differs from the other four species with the presence of the flattened dorsal setae. *Stenodemaplebeja* is longer and differs from other species in the body length/pronotum width ratio equaling 4.9–5.0 in females, while this ratio is < 4.4 in other species. In contrast to other species, *Stenodemasericans* is pale, without dark stripes on pronotum and hemelytron, and has parameres different from S. *algoviensis* and *S.holsata*, with apical half of the right paramere as wide as basal part, and the left paramere without outgrowth or swelling near the apical process (Wagner 1974: fig. 9d–f). Refer to the notes section after the diagnosis of *S.holsata* for the differences between *S.algoviensis* and *S.holsata*.
3. *S.laevigata* group includes species with frons not protruding above clypeus (Fig. 1H) and hind femora without spines and tapered apical region (Fig. 2E). According to Zheng (1981a), *S.antennata* Zheng, 1981 is close to *S.laevigata*, but much larger, with a female body length of 11. *Stenodemalongula* Zheng, 1981 might be close to *S.laevigata* as well (Zheng, 1981a), although this requires further verification.
4. *S.turanica*- *virens* group includes species with frons protruding above the clypeus base (Fig. 2C) and hind femur lacking spines and apical region tapered (Fig. 2B, C). *Stenodemacrassipes* is close to *S.virens* and *S.turanica*. However, it differs from them in the widened hind femur, which is 4–5× as long as wide, and the antennal segment II in female is widened basally with long and dense setae. Based on the drawings of the head and hind tibia in Zheng (1981a), *S.tibeta* Zheng, 1981 also belongs to this group. Nonnaizab and Jorigtoo (1994) compared *S.deserta* Nonnaizab & Jorigtoo, 1994 with *S.virens*, and noted that the former was different in the body structure and the paramere shape. However, those differences can be intraspecific variability, because according to our examinations, the parameres and vesica of *S.virens* are very similar to those depicted in Nonnaizab and Jorigtoo (1994: figs 4–6, 7G–I), and those two species could be conspecific. According to Zheng (1981b), *S.hsiaoi* is similar to *S.virens* and *S.turanica* in the habitus and male genitalia. The same is true for *Stenodemamongolica* Nonnaizab & Jorigtoo, 1994 (Nonnaizab and Jorigtoo 1994: figs 7–12); however, according to the original description it has flattened setae on the antennal segment I. The Nearctic species *S.vicina* (Provancher, 1872), *S.imperii* Bliven, 1858, *S.sequoia* Bliven, 1955, and *S.pilosipes* Kelton, 1961 are allied to the species of *virens*- *turanica* group (Bliven 1955, 1958; Kelton 1961).
5. *S.sibirica* group includes species with the frons protruding above the clypeus base (as in Fig. 1C), its hind femur does not have spines, and it is not tapering towards apex (Fig. 2F). *Stenodemanippon* is very similar to *S.sibirica* and *S.rubrinervis*, although distinctly differs from them in the salient features and genital structures (Yasunaga 2019). *Stenodemakhenteica* is also within this group and differs from *S.nippon*, *S.sibirica*, and *S.rubrinervis* in the antennal segment I shorter than pronotum and distinctly narrower than the eye diameter. Many other species described from China, most probably, belong to this group, and some of them might be conspecific with the species listed above. We had an opportunity to examine the paralectotype of *S.alpestris* Reuter, 1904 preserved at ZISP. In salient features and measurements this species is identical with *S.rubrinervis*. Horváth (1905) did not compare this species with *S.alpestris*, and possibly he was not aware of it. The lectotypes of both species should be examined to draw conclusion on their status. *Stenodemagridellii* Hoberlandt, 1960 has similar parameres to *S.sibirica*, but it has a smaller body (Hoberlandt 1960). Although in the drawings of Hoberlandt (1960)*S.gridellii* is shorter than *S.sibirica*, the provided measurements for the former fit those for *S.sibirica*Zheng (1981a) compared *S.alticola* Zheng, 1981 with *S.gridellii*, but wrote that the former is longer (males 6.7–6.8, female 7.5–7.6), having the erect setae on antennal segment I and its antennal segment II/I length ratio was 2.5. All those characters correspond to *S.rubrinervis*. Therefore, *S.alticola* and *S.rubrinervis* can be closely related or even synonymous. According to Zheng (1981a), *S.nigricalla* Zheng, 1981 is similar to *S.chinensis* (from the *turanica*- *virens* group). Judging from the drawings of the male genitalia, the shape of vesica of *S.nigricalla* is more similar to specimens from Siberia with short ridge on vesica (see Notes for the diagnosis of *S.sibirica*; Zheng 1981a: figs 13D–F, 19). However, the right paramere of this species has a longer apical process, than in those specimens and it is more similar to *S.rubrinervis* (Zheng 1981a: fig. 18; Yasunaga 2019: fig. 8a, e). Zheng (1981a) compared *S.angustata* Zheng, 1981 with *S.nigricalla*. However, the latter is more similar to *S.nippon* in the shape of the right paramere with elongate apical process and the presence of long and narrow vesica lobe at the left hand side (Yasunaga 2019: fig. 7C, F, G; Zheng 1981a: figs 20, 22). Tang (1994) compared *S.qulininginensis* Tang, 1994 with *S.nigricalla*, and, most probably, it also belongs to the *sibirica* group. Zheng (1992) noted that *S.daliensi*s Zheng, 1992 is similar to *S.alticola* and *S.gridellii* and differed from them in the body shape and coloration. According to Reuter (1904)*S.elegans* has the hind femur without spines and not tapering apically and its frons is protruded above clypeus, which also corresponds to the *sibirica* group.

We could not place *S dorsalis* (Say, 1832) and *S.parvula* Zheng, 1981 to any group listed above. Kelton (1961) proposed to treat *S.dorsalis* described from the Eastern USA as nomen nudum, because there were no records for it since the original description. *Stenodemaparvula* could be close to *S.holsata* or *S.laevigata* because its frons does not protrude above the clypeus; however, the information on the hind femur shape or genitalia structures for this species were not provided (Zheng 1981a).

### ﻿Phylogenetic relationships between species

The resulted trees from the Bayesian analyses are provided in Figs 14–19, and those resulted from the RaxML analyses are provided in Suppl. material 3.

All analyses show that widely distributed Palearctic species are monophyletic with high supports, as well as *S.rubrinervis* and *S.sericans* (P = 100, BS > 92). The COI sequences of two species from Nearctic, *S.pilosipes* and *S.vicina*, were included in the analyses. *Stenodemavicina* forms a clade (PP = 96 and 94, BS = 89 and 87 for COI and full datasets, respectively). However, *S.pilosipes* forms a clade only in the Bayesian analysis based on the full dataset (PP = 53), and in other cases one of the specimens is closer to *S.vicina* rather than to the second specimen of its species. *Stenodemacalcarata* and *S.pilosa* always form sister group relationships (PP = 94–100, BS = 66–83).

The topologies built on COI only and the full dataset comprise the greatest number of specimens and species, and they are very similar. They show that the clade formed by *S.calcarata* and *S.pilosa* (subgenus Brachystira) forms sister group relationships with the clade comprising all other *Stenodema* species (nominative subgenus), and the latter has the following supports: PP = 100 and 87, BS = 67 and 70 for COI and full datasets, respectively. Within this clade, *S.holsata*, *S.laevigata*, and *S.sericans* form a clade (PP = 100 and 89, BS = 82 and 75 for COI and full datasets, respectively). In the analyses based on COI only, the relationships between those three species are unresolved. However, in the phylogeny based on the full dataset, *S.sericans* forms a clade with *S.laevigata* although with low supports (PP = 63, BS = 55). *Stenodemapilosipes*, *S.sibirica*, *S.rubrinervis*, *S.turanica*, *S.vicina*, and *S.virens* form a clade (PP = 97 and 100, BS = 77 and 91 for COI and full datasets, respectively). Among those species, *S.turanica* and *S.virens* are sister groups (PP = 100 and 99, BS = 80 and 87 for COI and full datasets, respectively), and *S.pilosipes* and *S.vicina* also form a clade (PP = 100, BS = 98 in both analyses). Those two pairs show reciprocal monophyly (PP = 100 and 99, BS = 77 and 89 for COI and full datasets, respectively). *Stenodemasibirica* and *S.rubrinervis* form a clade in Bayesian analysis (PP = 100 and BS = 62 full dataset), and in the RaxML analysis with COI and 16S rRNA (BS = 90).

The phylogeny based on the reduced dataset with COI and 16S rRNA has the topology corresponding to those obtained based on COI and full datasets.

The results obtained with 16S rRNA have a different topology. In this case, *S.turanica* forms sister group relationships with the clade comprising other species, although with low support (PP = 67). *Stenodemavirens* forms sister group relationships with the rest of *Stenodema* species (PP = 89). *Stenodemasibirica* and *S.rubrinervis* form a clade (PP = 97, BS = 85), which is a sister group to the clade, formed by *S.calcarata*, *S.holsata*, *S.laevigata*, and *S.pilosa* (PP = 86). *Stenodemalaevigata* is a sister group to a clade formed by other three species (PP = 85, BS = 68). *Stenodemaholsata* is a sister group to a *S.calcarata*+*S.pilosa* clade (PP = 94, BS = 83).

### ﻿Intraspecific phylogenetic relationships

At least some analyses show genetic structure within *S.calcarata*, *S.pilosa*, *S.holsata*, and *S.laevigata*. Analyses based on 16S rRNA and reduced dataset do not show the structure within *S.pilosa* and *S.laevigata*.

The phylogenetic structure within *Stenodemapilosa* is present only in the results of analyses based on COI and full datasets because Nearctic species are included there. The specimens of this species are split into three main clades: two Nearctic and one Palearctic. One of the Nearctic clades (PP = 100 for both, BS = 88 and 84 or COI and full datasets, respectively) is a sister group to the rest of the specimens. The clade comprising some Nearctic and all Palearctic specimens has low to average supports (PP = 88 and 82, BS = 55 and 62 for COI and full datasets, respectively). This clade splits into two groups: one of them Nearctic (PP = 100 for both, BS = 100 and 99 for COI and full datasets, respectively), and the second one is Palearctic (PP = 100 and 92, BS = 99 and 94 for COI and full datasets, respectively).

In the analyses based on COI and full dataset, representatives of *S.calcarata* from the southern side of Caucasus (Iran, Georgia, Turkey) and a single specimen from Germany form a clade with the highest support, and it is a sister group to the rest of the specimens of this species (PP = 100 and 97, BS = 90 and 71 for COI and full datasets, respectively). Specimens from East Asia (South Korea and Primorsky Territory) form a clade with the highest support, which is a sister group to the clade formed by the rest of the specimens (PP = 100 and 92, BS = 100 and 90 for COI and full datasets, respectively). Only 16S rRNA was obtained for the specimen from Stavropol Territory, and in the phylogeny based on the full dataset it is a sister group to the rest of the specimens (PP = 97, BS = 95). Two specimens from Germany form a clade (PP = 100 and 99 for COI and full datasets, respectively, and BS = 100 for both), and they are the sister group to the clade comprising most of the specimens from the Western Palearctic and a specimen from Altay Republic (PP = 55 and 51 COI and full datasets, respectively, BS = 82 for COI).

In the phylogenies based on 16S rRNA and the reduced dataset, specimens of *S.calcarata* from Georgia and Turkey form a clade with the highest supports. In the phylogeny based on 16S rRNA and the reduced dataset, single specimen from the East Asia (Primorsky Territory) included in those analyses has many substitutions. In the analysis based on the 16S rRNA it forms unresolved relationships with the clade, comprising the specimens from Georgia and Turkey (PP = 100, BS = 100) and the clade comprising the rest of the specimens (PP = 95, BS = 83). In the phylogeny based on the reduced dataset, the clade comprising species from Georgia and Turkey forms a reciprocal monophyly with the clade comprising the rest of the specimens including the one from the Primorsky Territory (PP = 99, BS = 58). In the phylogeny based on 16S rRNA specimen from Stavropol Province forms a clade with the clade comprising most of the specimens from the Western Palearctic and Altay Republic (PP = 100, BS = 95 in both datasets).

In the phylogenies based on COI and full dataset all specimens of *S.holsata* from France form a clade (PP = 93 and 94, BS = 87 and 88 for COI and full datasets, respectively), and it is a sister group to the clade formed by the rest of the specimens in the results of the Bayesian analysis (PP = 56 and 51 for COI and full datasets, respectively). Specimen from Karachay-Cherkessia forms a clade with the clade formed by the specimens from Northern and Central Europe (PP = 53 and 64 for COI and full datasets, respectively, BS = 99 for COI dataset). Only four specimens of *S.holsata* are included to the analyses based on 16S rRNA and reduced dataset. The specimen from Karachay-Cherkessia is a sister group to a clade formed by three specimens from northern Europe (PP = 96 and 91, BS = 82 and 76 for COI and full datasets, respectively).

In the phylogenies based on COI and full dataset, there is a clade within *S.laevigata* comprising specimens from Greece, Iran, and Voronezh Province (PP = 100 and 82, BS = 98 and 59 for COI and full datasets, respectively). Within this clade, the specimens from Voronezh Province and Germany are more closely related (PP = 100 and 99 for COI and full datasets, respectively, BS = 100 for both datasets). The results of the analysis based on the full dataset does not show any other clades within this species. The Bayesian inference analysis based on COI dataset also show, that the rest of the specimens except for the three specimens mentioned above and one from Iran, also form a clade (PP = 85). Within this clade, a specimen from Crimea forms sister group relationships with the rest of the specimens (PP = 95).

### ﻿Species delimitation

All analyses show identical results for the phylogeny built based on 16S rRNA. In the case of COI, ABGD delimits the smallest number of species, followed by GMYC. PTP and bPTP show identical results for this marker. In the analyses based on the combined datasets, GMYC results in the smallest number of species. For the reduced dataset, PTP, bPTP, and BPP show identical results. For the full dataset, BPP results in the largest number of species, and PTP and bPTP showed the identical number of species. All species delimitation analyses do not mix the specimens belonging to different widespread species. *Stenodemasibirica*, *S.turanica*, and *S.virens* each form a single species in all the cases.

All analyses suggested that *S.calcarata* can be a complex of at least three species: (1) Far Eastern clade (2) West Asian clade and a single specimen from Germany, (3) Euro-Siberian clade. Additionally, specimen from Stavropol Province, a clade with two specimens from Germany and specimen from Germany in the West Asian clade form separate clades in some analyses.

*Stenodemapilosa* also can be a species complex. In the analyses with Nearctic specimens (COI dataset and full dataset) the Palearctic representatives of this species are placed in a single species, and Nearctic sequences are grouped in two or three species.

*Stenodemalaevigata* was subdivided into different number of species depending on the analysis. All analyses based on 16S rRNA, ABGD analysis based on COI and GMYC analysis based on the reduced dataset with both markers, and GMYC, PTP and bPTP analyses for the full dataset place all representatives of this species together. Specimen from Crimea, specimen from Iran and the clade formed by the specimens from Voronezh Province, Greece and Germany are assigned in separated species each by some analyses. Additionally, the specimens from Greece also appeared as a separate species in few cases.

Analyses based on COI and full dataset result in three species within *S.holsata*: (1) all specimens from France, (2) specimen from Karachay-Cherkessia, (3) specimens from northern and Central European areas. Only four specimens (one from Karachay-Cherkessia and three from northern European areas) are included in the analyses based on 16S rRNA and reduced dataset. The analysis based on the reduced dataset shows that the specimen from Karachay-Cherkessia forms a separate species, the analysis based on 16S rRNA places all specimens of *S.holsata* into a single species.

### ﻿Interspecific genetic distances

Interspecific distances are 6–17% for COI and 5–12% for 16S rRNA, and they are provided in Suppl. material 4. The lowest distances for COI are between *S.turanica* and *S.virens* (6–7%). Those two species also have relatively low genetic differences with Nearctic species *S.pilosipes* and *S.vicina* (~ 7–8%). The highest distances for COI are between *S.pilosa* and *S.rubrinervis* (15–17%). For 16S rRNA, the distances between the two species pairs *S.sibirica* — *S.ribrinerve* and *S.turanica* — *S.virens* are the lowest (~ 5%), and the highest distances are between *S.pilosa* and *S.virens* (11–12%).

### ﻿Intraspecific genetic distances

For the COI analysis, seven, three and four specimens are included, respectively for *S.sibirica*, *S.turanica* and *S.virens* (Suppl. material 4). Although the specimens of *S.sibirica* and *S.virens* were collected in different regions (*S.sibirica*: from Altay to South Korea, *S.virens* from Finland, Caucasus, and Irkutsk Province), the diversity of their sequences is very low for both markers (< 0.12%). The COI sequences for *S.turanica* collected in Iran and Tyva Republic are identical. For 16S rRNA, a single specimen of *S.turanica* is included. There are five specimens of *S.sibirica* and two sequences of *S.virens*, and genetic distances within these species are < 0.1%.

*Stenodemaholsata* and *S.laevigata* have within species mean distance corresponding to 0.8–1.1% for COI and ~ 0.4% for 16S rRNA. The species delimitation analyses resulted in three groups within *S.holsata* for COI, and the distances between them are 1–4%. The largest number of groups delimited within *S.laevigata* is five for COI, and the distances between them are 1–3%.

The interspecific distances within *S.calcarata* and *S.pilosa* are the largest, ~4% for COI for both species, ~ 2% for 16S rRNA of *S.calcarata* and 0.1% for 16S rRNA of *S.pilosa*. The largest number of species resulted from the species delimitation analyses for *S.calcarata* and *S.pilosa* using COI are five and four, respectively. The distances between the groups within *S.calcarata* are 7–9%, and between groups of *S.pilosa* are 2–6%. The species delimitation analysis based on the 16S rRNA dataset showed four groups within *S.calcarata*, and the distances between them are 3–4%.

## ﻿Discussion

There are 57 species placed within *Stenodema*. In this work we focused on the seven trans-Palearctic species and provided their detailed morphological study. We compared them with other Palearctic and Nearctic species based on the material preserved at ZISP, MZH, and on previous publications. To facilitate the future work on this genus, we placed most of the Palearctic and Nearctic species into five groups based on the set of morphological characters (see Results sections). Among other Palearctic *Stenodema* species, *S.algoviensis*, *S.chinensis*, *S.crassipes*, *S.khenteica*, *S*, *nippon*, *S.plebeja*, and *S.sericans* distinctly differ from widely distributed Palearctic species. Information on other species is scarce. The results of the phylogenetic analyses based on the different datasets are mostly concordant, except for 16S rRNA. However, we consider the latter less reliable, because there is lower nucleotide diversity in this marker in comparison to COI.

We found that most of the species with wide distribution in the Palearctic can be identified using salient features, as well as male and female genitalia. Their monophyly was supported by the phylogenetic analyses. We synonymize *S.trispinosa* with *S.pilosa* (see Results for the details). The subgeneric composition of the genus is supported by the molecular data. Both species with spines on the hind femur, i.e., *S.pilosa* and *S.calcarata*, are contained in the subgenus Brachystira. They can be separated by many characters in external view, as well as male and female genitalia, and they form a well-supported clade. This group forms a reciprocal monophyly with the clade formed by all other species (subgenus Stenodema) in the analyses based on COI and combined datasets, although the analyses based on 16S rRNA do not support those results. In the phylogenies, *S.holsata* is close to *S.laevigata* and *S.sericans*. However, morphologically it is very similar to *S.algoviensis*, and the molecular data for the latter are needed to confirm those relationships. There are also some species from China, which might be close to either *S.holsata* or *S.laevigata*.

The species with the protruding frons (*S.rubrinervis*, *S.sericans*, *S.sibirica*, *S.turanica*, *S.vicina*, *S.virens*) form a clade in all phylogenies, except for the one, based on 16S rRNA.

*Stenodematuranica* and *S.virens* have minor differences in the external view, however, they differ in the male and female genitalia, and they form sister groups in the phylogenies. Most Nearctic *Stenodema* species are similar to those two species morphologically. This is also confirmed by the molecular based phylogenies based on COI and combined datasets, where *S.vicina* and *S.pilosipes* form a clade with *S.turanica* and *S.virens*. Some species described from China also might be part of this group.

*Stenodemasibirica* is very similar to *S.rubrinervis*, their differences in external view are also minor, and we could not find any reliable difference in the genitalia structures. Molecular studies show that those two species distinctly diverged from each other. Most of the species known from Asia (Hoberlandt 1960; Zheng 1981a; Tang 1994; Yasunaga 2019) can be closer to *S.sibirica* rather than to other widely distributed Palearctic species, and some of them might be synonymous with it.

The species delimitation analyses never place the specimens belonging to different species together, except for the Nearctic *S.pilosipes* and *S.vicina*. The interspecific distances are relatively high (> 6% for COI and > 4% for 16S rRNA). Although barcoding regions does not always fit for the species delimitation studies, including Miridae groups (e.g., Toews and Brelsford 2012; Jäckel et al. 2013; Namyatova et al. 2023), it can be reliable for those purposes in *Stenodema*. Hybridization is unlikely between the studied species. Another marker, 16S rRNA, shows less diversity than COI, and the phylogenetic results based on those two markers do not entirely correspond. However, 16S rRNA also confirms the monophyly of the widespread Palearctic species.

*Stenodemacalcarata*, *S.holsata*, *S.laevigata*, and *S.pilosa* show intraspecific structure and at least some species delimitation analyses split them into two or more groups. In all those species the morphological evidence to support those lineages were not found. In *S.holsata* and *S.laevigata* the differences between the subclades are much less than intraspecific differences (1–4% and 1–3% in COI, respectively). The differences between some groups of *S.calcarata* and *S.pilosa* might suggest the presence of the cryptic species. The differences in COI between Palearctic and all Nearctic groups of *S.pilosa* is 4–5%, and the differences between Nearctic groups reaches 6–7%, which is comparable to the differences between *S.turanica* and *S.virens* (~ 6–7%), and between *S.virens* and *S.vicina* (~ 7%). The differences between *S.calcarata* groupings are more pronounced and reach 7–8% for COI and 3–4% for 16S rRNA.

In previous works, interspecific differences within widely distributed species of other Mirinae were studied for the *Lygus* species only: *L.gemellatus* (Herrich-Schaeffer, 1835), *L.pratensis* (Linnaeus, 1758), *Lygusrugulipennis* Poppius, 1911, and *L.wagneri* Remane, 1955 (Namyatova et al. 2023). All those taxa are known from Europe and Asia. Among them, only the trans-Holarctic *L.rugulipennis* has significant intraspecific structure.

In *Stenodema* at least *S.calcarata* and *S.pilosa* have deep population structure with the genetic differences between the clades comparable to the intraspecific differences. The structure within *S.holsata* and *S.laevigata* is also present, but not so pronounced. However, our results are also affected by the geographic range of the specimens included in the analysis. *Stenodemacalcarata*, *S.holsata* and *S.pilosa* inhabit East Asia (Kerzhner and Josifov 1999; Yasunaga 2019); however, only specimens of *S.calcarata* from this region were included in the analysis and they form a distinct clade. Specimens from Siberia were included for both *S.calcarata* (Altay Province) and *S.pilosa* (Yakutia), but in both cases they cluster with the European specimens. There is a clade within *L.rugulipennis*, which comprises specimens from the Far East, Siberia, and Northern Europe (Namyatova et al. 2023). Other species with trans-Palearctic distribution (*L.gemellatus*, *L.punctatus*, *L.wagneri*) have very shallow intraspecific structure.

The specimens from Caucasus and East Asia might represent isolated lineages in *Stenodema*. In *S.calcarata* there is a clade, comprising specimens from Georgia, Iran, Turkey, but it also comprises single specimen from Germany. The specimens of *S.holsata* from Karachay-Cherkessia and specimens of *S.laevigata* from Iran have many unique substitutions. Those results might suggest a presence of refugia at least in southern side of Caucasus and East Asia, which was also hypothesized for other insects (e.g., Wahlberg and Saccheri 2007; Eberle et al. 2021).

In *S.holsata*, the specimens from South Europe (France) form a separate lineage. Lineages of the specimens from South Europe were not found in other studied species.

In *S.laevigata*, there is a clade, formed by the specimens from Greece, Voronezh Province, and Germany. Additionally, specimen from Crimea have unique substitutions. In *L.rugulipennis* two specimens from Voronezh Province also distinctly differ from other specimens of their species, however, they do not cluster with the specimens from Germany or southern Europe (Namyatova et al. 2023). Those results might suggest that South Europe, Voronezh Province, and Crimea also could serve as refugia.

We did not find noticeable differences between the sequences within *S.virens*, *S.turanica*, and *S.sibirica*, even though specimens from different regions were included in the analyses. More specimens of those species should be analyzed to draw any conclusions on their intraspecific differences.

Schwartz (2008) provided morphology-based phylogenetic analysis and revision of Stenodemini, where he delimited *Stenodema* group with predominantly Nearctic distribution. Among 10 genera within this group only *Stenodema* inhabits other regions. The fact that *Stenodema* is much more diverse in the Palearctic than in the Nearctic and the phylogenies obtained in this work, suggest that this genus originated in the Palearctic. Its representatives migrated to the Nearctic at least three times. First, the ancestors of the Nearctic species from the clade, comprising *S.pilosipes*, *S.turanica*, *S.vicina*, and *S.virens*, migrated to the Nearctic. Second, the ancestor of *S.pilosa* also migrated to the Nearctic, and, third, some its representatives formed a separate lineage in the Palearctic. Therefore, we hypothesize that in *Stenodema* the migration occurred in both directions. Another Holarctic genus, *Lygus*, most probably, originated in the Nearctic, and then migrated to the Palearctic at least two times (Namyatova et al. 2023). Therefore, the migration routes in Miridae genera occurred in both directions.

Studies on *Lygus* and *Stenodema* showed that the gene flow between the Nearctic and Palearctic lineages of the same or closely related species is unlikely. In other insects with a Holarctic distribution, Nearctic and Palearctic representatives can be genetically separated from each other (e.g., Martin et al. 2002; Maresova et al. 2019; Francuski et al. 2021), or the gene flow can persist between Nearctic and Palearctic populations of the same species (e.g., Kohli et al. 2018, 2021; Zubrii et al. 2022).

## Supplementary Material

XML Treatment for Stenodema calcarata

XML Treatment for Stenodema holsata

XML Treatment for Stenodema laevigata

XML Treatment for Stenodema pilosa

XML Treatment for Stenodema sibirica

XML Treatment for Stenodema turanica

XML Treatment for Stenodema virens

## Footnotes

For more references and a list of synonyms see Carvalho (1959), Schuh (1995), and Kerzhner and Josifov (1999).

For more references and a list of synonyms see Carvalho (1959), Schuh (1995), and Kerzhner and Josifov (1999).

For more references and a list of synonyms see Carvalho (1959), Schuh (1995), and Kerzhner and Josifov (1999).

For more references and a list of synonyms see Carvalho (1959), Schuh (1995), and Kerzhner and Josifov (1999).

For more references and a list of synonyms see Carvalho (1959), Schuh (1995), and Kerzhner and Josifov (1999).

For more references and a list of synonyms see Carvalho (1959), Schuh (1995), and Kerzhner and Josifov (1999).

For more references and a list of synonyms see Carvalho (1959), Schuh (1995), and Kerzhner and Josifov (1999).

## ﻿Additional information

### Author ORCIDs

Anna A. Namyatova https://orcid.org/0000-0001-9678-3430

Polina A. Dzhelali https://orcid.org/0000-0002-0741-3655

Fedor V. Konstantinov https://orcid.org/0000-0002-7013-5686

### Data availability

All of the data that support the findings of this study are available in the main text or Supplementary Information.

## Supplementary materials

Supplementary material 1 List of examined non-type specimens of *Stenodema* spp. Data type pdf https://binary.pensoft.net/file/1112640This dataset is made available under the Open Database License (http://opendatacommons.org/licenses/odbl/1.0/). The Open Database License (ODbL) is a license agreement intended to allow users to freely share, modify, and use this Dataset while maintaining this same freedom for others, provided that the original source and author(s) are credited.Anna A. Namyatova, Polina A. Dzhelali, Fedor V. Konstantinov

Supplementary material 2 Voucher information for the specimens used for the comparison and phylogenies of the Palearctic species Data type pdf https://binary.pensoft.net/file/1112641This dataset is made available under the Open Database License (http://opendatacommons.org/licenses/odbl/1.0/). The Open Database License (ODbL) is a license agreement intended to allow users to freely share, modify, and use this Dataset while maintaining this same freedom for others, provided that the original source and author(s) are credited.Anna A. Namyatova, Polina A. Dzhelali, Fedor V. Konstantinov

Supplementary material 3 RaxML trees for individual and combined datasets Data type pdf https://binary.pensoft.net/file/1112642This dataset is made available under the Open Database License (http://opendatacommons.org/licenses/odbl/1.0/). The Open Database License (ODbL) is a license agreement intended to allow users to freely share, modify, and use this Dataset while maintaining this same freedom for others, provided that the original source and author(s) are credited.Anna A. Namyatova, Polina A. Dzhelali, Fedor V. Konstantinov

Supplementary material 4 Genetic distances Data type pdf https://binary.pensoft.net/file/1112643This dataset is made available under the Open Database License (http://opendatacommons.org/licenses/odbl/1.0/). The Open Database License (ODbL) is a license agreement intended to allow users to freely share, modify, and use this Dataset while maintaining this same freedom for others, provided that the original source and author(s) are credited.Anna A. Namyatova, Polina A. Dzhelali, Fedor V. Konstantinov
