# Supplementary material for: ﻿Delimitation of the widely distributed Palearctic Stenodema species (Hemiptera, Heteroptera, Miridae): insights from molecular and morphological data
Source: Zookeys. 2024 Aug 13;1209:245–94. doi: 10.3897/zookeys.1209.124766 (PMC11336388; doi:10.3897/zookeys.1209.124766)
Supplement: Supplementary material 1 — List of examined non-type specimens of Stenodema spp. [file zookeys-1209-245_article-124766__-s001.pdf]

List of examined non-type specimens of *Stenodema* spp.

Stenodema calcarata

ARMENIA: Jrvezh [Dzhervezh] nr Yerevan, 40.18333°N 44.58333°E, 12 May 1938, Collector unknown, 1♂ (ZISP_ENT 00002710) (ZISP). Vayk [Soylan, Azizbekov], 39.69°N 45.46°E, 11 Jul 1934, Ter-Minasyan, 2♀ (ZISP_ENT 00002765, ZISP_ENT 00002766) (ZISP). **AZERBAIJAN:** Samaxa [Shemakha], 40.63333°N 48.63333°E, 02 Jul 1947 - 07 Jul 1947, Bogachev, 2♂ (ZISP_ENT 00002713, ZISP_ENT 00002714) (ZISP). **BELARUS:** Ambrosovichi, 55.2°N 29.666°E, 05 Sep 1925, Birulya, 1♀ (ZISP_ENT 00004877) (ZISP). **FRANCE:** [Bagneux, Muit du Sh], 05 Nov 2018, Collector unknown, 1♂ (ZISP_ENT 00013653) (ZISP). **GEORGIA:** Abastumani, 41.75611°N 42.82806°E, 15 Jun 1949, Kiritshenko, 1♂ (ZISP_ENT 00002705) (ZISP). Lata (Laty) on Kodor river, 43.03°N 41.48°E, 19 Aug 1905, Kalishevskiy, 1♂ (ZISP_ENT 00002703) (ZISP). Mtskheta [former Tiflis Government], 41.83333°N 44.7°E, 05 Jun 1933, Kirshenblat, 1♂ (ZISP_ENT 00002694) (ZISP). Skurcha Lake, mouth of the Kodori River, Sokhumi [Sukhum], 42.80091°N 41.16869°E, 10 May 1928, Y. Zimin, 1♀ (ZISP_ENT 00002767) (ZISP). Sokhumi [Sukhum], 43°N 40.96666°E, 13 Apr 1928, Y. Zimin, 1♂ (ZISP_ENT 00004875), 1♀ (ZISP_ENT 00002737) (ZISP). Tbilisi National Park nr Saguramo, 41.88836°N 44.7756°E, 670 m, 31 May 2022, A. Namyatova & S. Roth, 1♂ (ZISP_ENT 00013647) (ZISP). **IRAN (ISLAMIC REPUBLIC OF):** Gorgan [Astrabad], 36.83333°N 54.41666°E, 15 Mar 1904, Filippovich, 1♂ (ZISP_ENT 00004873) (ZISP). **KAZAKHSTAN: Almaty Prov.:** Apiary Zubenko, Bolshaya Almatinka, 44.81666°N 78.15°E, 17 Aug 1928, Shnitnikov, 1♂ (ZISP_ENT 00002706) (ZISP). Sarqant [Sarkand] Dzhungarskiy Alatau, 45.4°N 79.9°E, 28 Apr 1957, I. M. Kerzhner, 1♂ (ZISP_ENT 00002729) (ZISP). Topolevka, E of Sarkand, Dzhungar Alatau, 45.4°N 80.3333°E, 1145 m, 26 May 1957, I. M. Kerzhner, 1♂ (ZISP_ENT 00002717) (ZISP); 27 May 1957, I. M. Kerzhner, 1♂ (ZISP_ENT 00002730) (ZISP). **Kyzylorda Prov.:** Zhulek [Dzhulek], 44.28333°N 66.43333°E, 15 Aug 1910 - 21 Aug 1910, Kozhanchikov, 1♂ (ZISP_ENT 00013670) (ZISP). **KYRGYZSTAN:** Jalal-Abad [Dzhelalabad], Andizhan Distr., 40.929°N 73.009°E, 15 May 1909 - 20 May 1909, Ryzhikova, 1♂ (ZISP_ENT 00004871) (ZISP). Kyrgyz [Aleksandr] Mts. Range, Kenkol Valley, 42.5°N 72.36°E, 16 Jul 1930, Bianchi, 1♀ (ZISP_ENT 00004864) (ZISP). **LITHUANIA:** Jurbarkas [Jurburg], 55.06666°N 22.76666°E, 30 Apr 1905, Vinogradov and Nikitin, 1♂ (ZISP_ENT 00003674) (ZISP (OSAC). **POLAND:** Bialystok [Belostok], 53.128°N 23.151°E, 01 Aug 1913, Trzheskovskiy, 1♀ (ZISP_ENT 00002759) (ZISP). **RUSSIAN FEDERATION: Altai Terr.:** Beloretskiy [Beloretskaya], Zmeinogorsk, S Altay, 51.03333°N 82.73333°E, 05 Aug 1930, F.K. Lukjanovitsh, 1♀ (ZISP_ENT 00002735) (ZISP). Tigirekskiy National Reserve, 51.05°N 82.98333°E, 27 Jun 2009, A. Knyshov, *Calamagrostis epigejos* (L.) Roth (Poaceae), 1♀ (ZISP_ENT 00003719) (ZISP). **Amur Prov.:** Byssa River, 2km downstream of Kukuy stream, 52.34427°N 131.33595°E, 310 m, 09 Jun 2007, Veselova & Ryvkin, 1♀ (ZISP_ENT 00013644) (ZISP). Salokachi, Valley of Bureya River, 49.68333°N 130.78333°E, 08 Sep 1946, L. Kiritshenko, 1♀ (ZISP_ENT 00004869) (ZISP). **Bashkortostan Rep.:** 2 km W from Arslanovo, 54.64901°N 54.37217°E, 197 m, 11 Jun 2019, F. Konstantinov & A. Namyatova, 1♂ (ZISP_ENT 00013651) (ZISP). **Belgorod**

Prov.: Borisovka, "square field", 50.59943°N 35.97669°E, 25 Jun 2018, F. Konstantinov, 1♂ (ZISP_ENT 00013660) (ZISP). **Bryansk Prov.:** Bryansk, 53.25°N 34.4°E, 14 Jul 1927, Stark, 1♀ (ZISP_ENT 00002763) (ZISP). Dobrun, 53.18645°N 34.23373°E, 12 May 2018, D. Kucherov, (Poaceae), 1♂ (ZISP_ENT 00003726) (ZISP). **Irkutsk Prov.:** Bunbuy, 56.38306°N 99.02861°E, 17 Aug 1915, Valdaev, 1♂ (ZISP_ENT 00004872) (ZISP). Mal'ta, 59.38333°N 103.85°E, 18 Jul 1909, Plyuschinskaya, 1♀ (ZISP_ENT 00004867) (ZISP). **Karelia Rep.:** 1.1 km NNE of Ruskeala, nr. Mramornaya mt., 61.94306°N 30.60442°E, 18 Jun 2022, A. Namyatova, V. Tyts, P. Dzhelali, 1♀ (ZISP_ENT 00007386) (ZISP). **Khabarovsk Terr.:** Malyshevskaya duct, 48.57833°N 134.97889°E, 13 May 1911, Soldatov, 1♀ (ZISP_ENT 00004868) (ZISP). Mayka, 59.98333°N 145.36667°E, 13 Jun 1901, Shmidt, 1♀ (ZISP_ENT 00007331) (ZISP). **Leningrad Prov.:** Gatchina, Roshchinskaya st., 59.57361°N 30.14306°E, 16 Aug 2020, V. Tyts & S. Davletshin, 1♀ (ZISP_ENT 00013662) (ZISP). Gorelovo, 59.76666°N 30.1°E, 29 May 1898 - 07 Jun 1898, Novotortsev, 1♀ (ZISP_ENT 00002769) (ZISP); 06 Sep 1898, Novotortsev, 1♀ (ZISP_ENT 00002734) (ZISP). Lebyazhye, 59.93333°N 29.41666°E, 12 May 1900, Bianchi, 1♀ (ZISP_ENT 00004866) (ZISP). Lopukhinka, 59.733°N 29.401°E, 11 Aug 1894, Bianchi, 1♀ (ZISP_ENT 00004865) (ZISP). Peterburgskaya Government, 59.91666°N 30.3°E, 14 Jul 1895, V.L. Bianchi, 1♂ (ZISP_ENT 00002697) (ZISP). Rakovichi, 7 verst S of Luga, 58.65056°N 29.84°E, 30 May 1897 - 03 Jun 1897, Pleske, 1♀ (ZISP_ENT 00002770) (ZISP). Shuvalovo, 60.05°N 30.3°E, 25 Jun 1897, Jakobson, 1♀ (ZISP_ENT 00007382) (ZISP). **Murmansk Prov.:** NW border of Luvenga vil., 67.10687°N 32.70522°E, 26 Jul 2021, A. Namyatova, V. Tyts, A. Vodopyanova, 1♂ (ZISP_ENT 00013658) (ZISP). **North Ossetia Rep.:** Barzikau nr Vladikavkaz, 42.81666°N 44.3°E, 31 Jul 1925, A. N. Kiritshenko, 1♂ (ZISP_ENT 00004876) (ZISP). **Primorsky Terr.:** 15km SE Nakhodka, 42.72509°N 133.13497°E, 07 Jun 2016, Belokobylskij, 1♀ (ZISP_ENT 00013641) (ZISP). Evseevka [former Imanskiy Uezd], 44.41666°N 132.88333°E, 24 Aug 1982, Zinoviev, 1♀ (ZISP_ENT 00002728) (ZISP). **Pskov Prov.:** Akulovo, Novorzhevskiy uezd, 57.185°N 29.4876°E, 01 Jun 1915, N. Kuznetsov, 1♀ (ZISP_ENT 00002762) (ZISP). Dubets Lake nr Novorzhevsk, 56.96°N 30.02°E, 03 Aug 1915 - 08 Aug 1915, N. Kuznetsov, 1♀ (ZISP_ENT 00004863) (ZISP). Sebezhskiy National Park, 56.15917°N 28.34306°E, 04 Jul 2019, V. D. Tyts, *Calamagrostis epigejos* (L.) Roth (Poaceae), 1♀ (ZISP_ENT 00003718) (ZISP). Shchepets [former Yaz'vo Volost', Gdov Government], 58.75°N 28.2°E, 16 Jul 1901, Ivanov, 1♂ (ZISP_ENT 00013671) (ZISP). **Sakhalin Prov.:** Coast of Kozlinaya Bay, 12 May 1900, Shmidt, 1♀ (ZISP_ENT 00013668) (ZISP). Tret'yakovo, Kunashir Island, 43.98889°N 145.64167°E, 06 Sep 1971, Narchuk, 1♂ (ZISP_ENT 00002712) (ZISP). **Stavropol Terr.:** Zheleznovodsk, Caucasia, 44.13333°N 43.01666°E, 19 Jun 2010, D. Gapon, *Calamagrostis epigejos* (L.) Roth (Poaceae), 1♀ (ZISP_ENT 00003720) (ZISP). **Tyumen Prov.:** Tobolsk, Cemetery, 58.18333°N 68.23333°E, 24 Jul 1927, Samko, 1♀ (ZISP_ENT 00002711) (ZISP). Tobolsk, Staryi Tobol River, 58.14981°N 68.21759°E, 17 Jun 1926, Samko, 1♀ (ZISP_ENT 00002764) (ZISP). **Ulyanovsk Prov.:** Staromaynskiy Bay coast, ap. 1km S from Verkhnyaya Matrosovka, 54.6321°N 49.0385°E, 100 m, 05 Jul 2021, A. Namyatova, 1♀ (ZISP_ENT 00003717) (ZISP). **Yaroslavl Prov.:** Zhedenovo, 58.089°N 40.188°E, 1915, Shestakov, 1♂ (ZISP_ENT 00004870), 1♀ (ZISP_ENT 00002734) (ZISP). **TAJIKISTAN:** Kondara Canyon, Valley of Varzob River, 38.83333°N 68.83333°E, 1100 m, 23 Aug 1937, Gussakovskiy, 1♂ (ZISP_ENT 00002708) (ZISP). Sarkoron,

Khozratishokh, 38.5°N 70.43°E, 1900 m, 18 Jun 1958, Lopatin, 1♂ (ZISP_ENT 00002716) (ZISP). **TURKEY: Kars:** Kars, Kagizman Yolu nr Pasli, 40.27085°N 42.9546°E, 1763 m, 04 Jun 2009, D. Gapon, 1♀ (ZISP_ENT 00013633) (ZISP). **TURKMENISTAN:** Tashkepri on Murgap River, 36.28333°N 62.63333°E, 01 Apr 1955, V.I. Tobias, 1♂ (ZISP_ENT 00002715) (ZISP). **UKRAINE:** Aleshki [Tsyurupinsk, Taurida Gov.], lower of Dnepr river, 46.61667°N 32.7°E, 22 May 1927, Egorov, 1♀ (ZISP_ENT 00002768) (ZISP); 16 Jun 1927, Egorov, 1♀ (ZISP_ENT 00002766) (ZISP). Chernihiv Prov. Sosnitsa, 51.51666°N 32.5°E, 10 Jul 1916, A.A. Stackelberg, 1♂ (ZISP_ENT 00002707) (ZISP). Peresyp' of Tiligul'skiy liman, 46.63333°N 31.1°E, 11 Jul 1926, Znoiko, 1♂ (ZISP_ENT 00004874) (ZISP). **UZBEKISTAN: Toshkent Shahri:** Toshkent [Tashkent , Tashkend], 41.3°N 69.28333°E, 19 Jun 1924 - 22 Jun 1924, Martynova, 1♀ (ZISP_ENT 00002736) (ZISP). Klyuchi, near Turkestan city, 39.62°N 66.25°E, Jun 1909 - Jul 1909, Trizna, 1♀ (ZISP_ENT 00013669) (ZISP).

Stenodema holsata

GEORGIA: Valley of Antsal-Or River, Tiflis Government, 41.75°N 46.22°E, 11 Aug 1913, A. Mlokossiewich, 1♀ (ZISP_ENT 00002741) (ZISP). **GERMANY: Baden-Wuerttemberg:** Bad Herrenalb [Herrenalb], 48.78333°N 8.41666°E, 12 Jul 1898, Adelung, 1♂ (ZISP_ENT 00004905) (ZISP). **KAZAKHSTAN: East Kazakhstan Prov.:** Saur Mt. Range SE Zaysan, 49.11666°N 86.4°E, 10 Jun 1927 - 11 Jun 1927, Dobrzhanskiy and Kerkis, 1♂ (ZISP_ENT 00004907), 1♀ (ZISP_ENT 00002743) (ZISP). **NORWAY:** Saltdal, Hellevskogen, 66.7162°N 15.4679°E, 580 m, 08 Aug 2020, Hansen, Shaw, Solodovnikov, 1♀ (ZISP_ENT 00013667) (ZISP). **RUSSIAN FEDERATION: Bashkortostan Rep.:** Dvoynishi, nr Katav-Ivanovsk, 54.483°N 58.281°E, 27 Jun 1926, Vakulenko, 1♀ (ZISP_ENT 00002746) (ZISP); 30 Jun 1926, Vakulenko, 1♀ (ZISP_ENT 00004906) (ZISP). **Dagestan Rep.:** Khochaldag Mt., 42.73333°N 46.26666°E, 09 Aug 1913, A. Mlokossiewich, 1♂ (ZISP_ENT 00003639) (ZISP). **Karachay-Cherkessia Rep.:** Valley of Teberda River, 43.34268°N 41.67152°E, 30 Jul 2021, Golub, 1♂ (ZISP_ENT 00013664) (ZISP). **Karelia Rep.:** 2.5 km W of Ruskeala, 61.93319°N 30.5371°E, 20 Jun 2022, A. Namyatova, V. Tyts, P. Dzhelali, 1♀ (ZISP_ENT 00007905) (ZISP). Solovetskie islands, nr monastery, 65.03°N 35.723°E, 16 Jul 1895 - 06 Aug 1895, Birulya, 1♀ (ZISP_ENT 00004909) (ZISP); 15 Jun 1896, Birulya, 1♀ (ZISP_ENT 00002748) (ZISP). **Khabarovsk Terr.:** Ozerpakh, 53.03583°N 141.22861°E, 05 Jul 1915, Chernavin, 1♀ (ZISP_ENT 00002744) (ZISP). **Khanty-Mansi Distr.:** Malyy Yugan River, Surgut Distr., 60.66667°N 73.9°E, 19 Aug 2003, Ryvkin, 1♀ (ZISP_ENT 00002740) (ZISP). **Komi Rep.:** Bol'shezemel'skaya tundra, stations 6, Shapkina River, 66.73306°N 52.72861°E, no date provided, Richter, 5♂ (ZISP_ENT 00004915, ZISP_ENT 00004904, ZISP_ENT 00004912, ZISP_ENT 00002800, ZISP_ENT 00002802), 1♀ (ZISP_ENT 00004910) (ZISP). Kydziras river, basin of Bol'shaya Synya [Syn'], Pechora, 65.26667°N 58.23333°E, 09 Aug 1908, Zhuravskiy, 1♂ (ZISP_ENT 00004891) (ZISP). Ust'-Tsyl'ma [Pechorskiy Uezd, Arkhangelsk Government], 65.4411°N 52.15°E, 21 Aug 1905, Zhuravskiy, 1♂ (ZISP_ENT 00004892) (ZISP). **Krasnoyarsk Terr.:** Bazaikha River nr Krasnoyarsk, 55.96111°N 92.81333°E, 1895, Ulrich, 1♀ (ZISP_ENT 00002751) (ZISP). **Leningrad**

Prov.: Chernaya Lakhta, 59.967°N 29.248°E, 04 Jun 1897, Bianchi, 1♀ (ZISP_ENT 00002749) (ZISP). Gorelovo, 59.76666°N 30.1°E, 29 May 1898 - 07 Jun 1898, Novotortsev, 1♀ (ZISP_ENT 00004911) (ZISP). Gorskaya, 60.049°N 29.984°E, 27 Jul 1897, Birulya, 1♂ (ZISP_ENT 00013676) (ZISP). Kharlamova Gora, 58.97361°N 29.32972°E, 17 Jul 1898, Bikhner, 1♀ (ZISP_ENT 00002742) (ZISP). Lebyazhye, 59.93333°N 29.41666°E, 25 Aug 1899, Bianchi, 1♂ (ZISP_ENT 00002799) (ZISP). Lobanovo, 59.817°N 30.932°E, 13 Aug 2020, Barovsky, 1♂ (ZISP_ENT 00003625) (ZISP). Lopukhinka, 59.733°N 29.401°E, 26 Jul 1894, Bianchi, 1♂ (ZISP_ENT 00004903) (ZISP); 29 Jul 1894, Bianchi, 1♂ (ZISP_ENT 00003636) (ZISP); 01 Aug 1894, Bianchi, 1♀ (ZISP_ENT 00004908) (ZISP). Romanovka nr Kingisepp [Yamburg], 59.36666°N 28.6°E, 04 Aug 1905, Barovsky, 1♂ (ZISP_ENT 00002801) (ZISP). Roshchino [Rayvola], Finland Railway, 60.25°N 29.61666°E, 16 Jul 1896, Prikhodko, 1♀ (ZISP_ENT 00013675) (ZISP). Sinyavino, Lobanovo Dock [Shlisselburgskiy Uezd], 59.91048°N 31.08563°E, 22 Jul 1920, Barovsky, 1♀ (ZISP_ENT 00002745) (ZISP). **Murmansk Prov.:** Dal'nie Zelentsy, E coast of Promernoe Lake, 69.12069°N 36.06136°E, 32 m, 29 Jul 2018, A. Namyatova, 1♂ (ZISP_ENT 00002804), 1♀ (ZISP_ENT 00013654) (ZISP). Kandalakshskiy Nat Reserve, base nr Luvenga vil., 67.10363°N 32.69868°E, 24 Jul 2021, A. Namyatova, V. Tyts, A. Vodopyanova, 1♂ (ZISP_ENT 00002803) (ZISP). South part of Luvenga vil, ca. 300m from Luvenga River, 67.10155°N 32.70979°E, 24 Jul 2021, A. Namyatova, V. Tyts, A. Vodopyanova, 1♀ (ZISP_ENT 00013663) (ZISP). **Novgorod Prov.:** Kleigels's Mill on Tigoda River, 59.286°N 31.571°E, 19 Jun 1903, Semenov, 1♀ (ZISP_ENT 00002747) (ZISP). Tigoda River, 59.286°N 31.571°E, 01 Aug 1903, Semenov, 2♀ (ZISP_ENT 00013677, ZISP_ENT 00013674) (ZISP); 06 May 1904, Semenov, 1♂ (ZISP_ENT 00013678) (ZISP); 23 Jul 1909, Semenov, 1♂ (ZISP_ENT 00003638) (ZISP). **Yamalo-Nenets Distr.:** Sob' River, Bolshoy Ural, 66.86769°N 65.75703°E, 19 Jul 1925, Fridolin, 1♀ (ZISP_ENT 00003679) (ZISP). **Yaroslavl Prov.:** Zhedenovo, 58.089°N 40.188°E, 1915, Shestakov, 1♂ (ZISP_ENT 00003637) (ZISP).

Stenodema laevigata

ARMENIA: Darachichag [Taghkadzor], Yerivanskiy Dist., 40.53333°N 44.75°E, Malyushenko, 1♀ (ZISP_ENT 00013672) (ZISP). Idzhevan [Ijevan], 40.86666°N 45.13333°E, 26 May 1955, Zagulyaev, 1♀ (ZISP_ENT 00002750) (ZISP). Karmrakar, 40.86666°N 43.88333°E, 16 Jun 1955, Loginova, 1♂ (ZISP_ENT 00004926) (ZISP). Vanadzor [Kirovakan], 40.8°N 44.46666°E, 07 Aug 1937, Ter-Minasyan, 1♂ (ZISP_ENT 00002699) (ZISP). **AZERBAIJAN:** Baliton [Paleton] (1200), Astara Distr., Talysh, 38.8°N 48.632°E, 19 Jul 1932, Znoiko, 1♀ (ZISP_ENT 00004924) (ZISP). Lirik, Mts. Talysh, Lenkoran', 38.73333°N 48.83333°E, 06 Jul 1909, Kreis, 1♂ (ZISP_ENT 00003670) (ZISP). **BULGARIA:** Vitosha mountain, Sofia, 42.55°N 23.23°E, 07 Jun 1953, Martino, 1♀ (ZISP_ENT 00003631) (ZISP). **GEORGIA:** Aleksandersgilf on the Khrami River, 41.59°N 44.08°E, 1569 m, 19 Aug 1931, Dyakonov, 1♂ (ZISP_ENT 00004916), 2♀ (ZISP_ENT 00004923, ZISP_ENT 00004925) (ZISP). Azhary near Chkhalta, Kodor River, Abkhazia, 43.037°N 41.44°E, 14 Jul 1905, Kalishevskiy, 1♂ (ZISP_ENT 00003673) (ZISP). Kodzori nr Tbilisi [Tiflis], 41.65°N 44.68333°E, 1400 m, 14 Jun 1916 - 15 Jun 1916,

Andrievskiy, 1♀ (ZISP_ENT 00002758) (ZISP). Lagodekhi, Tiflis Government, 41.827°N 46.269°E, 28 Jul 1909, A. Mlokossiewich, 2♂ (ZISP_ENT 00004927, ZISP_ENT 00003675) (ZISP); 10 Jul 1916, V. Mlokossevich, 1♀ (ZISP_ENT 00002756) (ZISP). Northwestern Caucasus, 40.09°N 35.72°E, no date provided, Stark, 1♂ (ZISP_ENT 00003672) (ZISP). Sokhumi [Sukhum], 43°N 40.96666°E, 30 Sep 1927, G. Zimin, 1♂ (ZISP_ENT 00004921) (ZISP). Tsebelda near Sokhumi, Abkhazia, 43.01666°N 41.26666°E, Aug 1931, Voronov, 1♀ (ZISP_ENT 00002755) (ZISP). [Dzyankvish nr Azhar on Kodor River, Sukhum], 19 Jul 1905, Kalishevskiy, 1♀ (ZISP_ENT 00013673) (ZISP). **GERMANY: Baden-Wuerttemberg:** Bad Herrenalb [Herrenalb], Schwartzwald, 48.80094°N 8.43559°E, 25 Aug 1898, Adelung, 1♂ (ZISP_ENT 00003669) (ZISP). **GREECE:** Tymfristos Mt, 38.94484°N 21.82367°E, 20 Jul 2008, Lukhtanov & Shapoval, 1♂ (ZISP_ENT 00013650) (ZISP). **MOROCCO:** High Atlas Mts, Toubcal Nat. Park nr Imil Settim, 31.13306°N 7.91728°W, 1850 m, 16 Sep 2013 - 22 Sep 2013, D. Gapon, 1♀ (ZISP_ENT 00003723), 1;u (ZISP_ENT 00003723) (ZISP). **POLAND:** Belyanskaya grove, nr Warszawa [Varshava], 52.25°N 21°E, Jul 1898 - 05 Aug 1898, Barshchevsky, 1♂ (ZISP_ENT 00003667) (ZISP). Bialystok [Belostok], 53.128°N 23.151°E, 23 May 1913, Trzheskovskiy, 1♀ (ZISP_ENT 00003661) (ZISP). Bodzentyn, 50.93333°N 20.95°E, 20 Jul 1895, Jakobson, 1♀ (ZISP_ENT 00002754) (ZISP). Plock [Plotsk], 52.53333°N 19.68333°E, 30 Apr 1906, Molchanov, 1♂ (ZISP_ENT 00003676) (ZISP); 06 May 1906, Molchanov, 1♀ (ZISP_ENT 00002738) (ZISP). **RUSSIAN FEDERATION: Adygeya Rep.:** Abago Mt., Maykop Dist., 43.81666°N 40.18333°E, 03 May 1935, Deev, 1♂ (ZISP_ENT 00002698) (ZISP). Maykop, 44.36°N 40.05°E, 30 Jun 1927, G. Semenov, 1♀ (ZISP_ENT 00004922) (ZISP). **Belgorod Prov.:** W part of Belogor'e State Nature Reserve, nr Borisovka, 50.62°N 35.95°E, 04 Jul 2019, A. Namyatova, 1♀ (ZISP_ENT 00013659) (ZISP). **Bryansk Prov.:** Bryansk, 53.25°N 34.4°E, 03 Aug 1927, Stark, 1♀ (ZISP_ENT 00002753) (ZISP); 08 Aug 2021, V. D. Tyts, 1♂ (ZISP_ENT 00013666) (ZISP). **Dagestan Rep.:** Gunib, 42.38333°N 46.95°E, 25 Jul 1924, Ryabov, 1♂ (ZISP_ENT 00003668) (ZISP). Okyuztau Mt. Gimrinskiy Mts. Range, 42.68333°N 47.01666°E, 16 Jul 1944, Ryabov, 1♀ (ZISP_ENT 00002757) (ZISP). **Kabardino-Balkaria Rep.:** Nalchik, 43.48333°N 43.6°E, 07 Jul 1914, Golovleva, 1♀ (ZISP_ENT 00002752) (ZISP); 14 Jul 1914, A. Kiritshenko, 1♂ (ZISP_ENT 00004920) (ZISP). **Kaliningrad Prov.:** Curonian Spit, 500m SW from Rybachiy, 55.15591°N 20.83632°E, 10 m, 15 Aug 2022, A. Namyatova, V. Tyts, P. Dzhelali, 1♀ (ZISP_ENT 00007921) (ZISP). **Karachay-Cherkessia Rep.:** Teberda, 43.45°N 41.73333°E, 02 Oct 1954, L. Arens, 1♂ (ZISP_ENT 00003666) (ZISP). **Pskov Prov.:** Sebezhskiy National Park, 56.15917°N 28.34306°E, 04 Jul 2019, V. D. Tyts, (Poaceae), 1♂ (ZISP_ENT 00003727) (ZISP). **Saratov Prov.:** Tatishchevskiy dist., Yagodnaya Polyana vill., 51.97028°N 45.60444°E, 21 Jul 2018, M. Nikelshparg, 1♀ (ZISP_ENT 00013649) (ZISP). **Ulyanovsk Prov.:** Staromaynskiy Bay coast, ap. 1km S from Verkhnyaya Matrosovka, 54.6321°N 49.0385°E, 100 m, 06 Jul 2021, A. Namyatova, 1♀ (ZISP_ENT 00013634) (ZISP). **Voronezh Prov.:** Kamennaya Steppe 11 km S Talovaya, 51.21666°N 40.71666°E, 19 Jun 1935, Stark, 1♀ (ZISP_ENT 00003678) (ZISP). Novousmanskiy dist., Shuberskoe, 51.74278°N 39.42278°E, 01 Aug 2018, Prob. Khitsova, 1♀ (ZISP_ENT 00013656) (ZISP). **UKRAINE: Crimea:** Alushta, 44.681°N 34.407°E, 02 Jul 1899, N. Kuznetsov, 1♂ (ZISP_ENT 00003671) (ZISP). Hurzuf [Gurzuf], 44.55°N 34.2833°E, 24 Jul 1927, Znoiko, 1♂ (ZISP_ENT 00003665) (ZISP). Karadag Nat Res, ecological trail, 1.3 km from Biostation, 44.1668°N

35.223°E, 212 m, 04 Jun 2018, A. Namyatova, 1♂ (ZISP_ENT 00013648) (ZISP). Privol'noe [Taushan-Bazar], 44.782°N 34.334°E, 16 Jul 1907, W. Pliginskiy, 1♂ (ZISP_ENT 00002704), 1♀ (ZISP_ENT 00002704) (ZISP). Chernivtsi [Chernovtsy], 48.288°N 25.959°E, 25 Sep 1960, I. M. Kerzhner, 1♀ (ZISP_ENT 00003677) (ZISP). Verkhovka [former Mahilyow uezd], 48.9°N 27.65°E, 21 May 1901 - 23 May 1901, Chekini, 1♀ (ZISP_ENT 00003660) (ZISP). **YUGOSLAVIA: Serbia:** Topcidersko Brdo [Topchider] nr Belgrad, 44.76666°N 20.45°E, 1927, Martino, 1♀ (ZISP_ENT 00011831) (ZISP).

Stenodema pilosa

KAZAKHSTAN: Akmola Prov.: Atbasar, 51.8°N 68.35°E, 13 Jul 1899, Ignatov, 1♂ (ZISP_ENT 00004948) (ZISP). **Aktobe Prov.:** Dzurun, 49.25389°N 57.58722°E, 09 Jul 1933, Formozov, 1♂ (ZISP_ENT 00014307) (ZISP). **Almaty Prov.:** Temir-su Gorge, Saur Mts. Ridge, 47.1°N 85.5°E, 03 Jul 1927, Dobrzhanskiy and Kerkis, 1♀ (ZISP_ENT 00014304) (ZISP). **Karaganda Prov.:** 30 km SE of Atasu [Zhana-Arka], 48.557°N 72.017°E, 09 May 1958, Loginova, 1♂ (ZISP_ENT 00004949) (ZISP). **Kostanay Prov.:** 250 km S Kustanai, Ak-Suat Lake, 51.41°N 64.47°E, 13 Jul 1946, Formozov, 1♀ (ZISP_ENT 00004945), 2♂ (ZISP_ENT 00004946, ZISP_ENT 00004947) (ZISP). **KYRGYZSTAN:** Ak-terek, 5 km N Gava, 41.3°N 72.81666°E, 23 Jul 1937, A. N. Kiritshenko, 1♀ (ZISP_ENT 00014308) (ZISP). **MONGOLIA: Eastern Aimak:** Darkhin-Tsagan-obo, 60 km ENE Bayan-Burd, 47.416°N 118.972°E, 21 Jul 1971, I. M. Kerzhner, 1♀ (ZISP_ENT 00014295) (ZISP). Khalkhin-Gol river, 33 km SW of Khalkh-Gol somon, 47.8°N 116.3°E, 19 Jul 1971, Kozlov, 1♂ (ZISP_ENT 00014297) (ZISP). **RUSSIAN FEDERATION: Orenburg Prov.:** Nr Orenburg, 51.76666°N 55.1°E, 01 Jun 1924, A.I. Ivanov, 1♀ (ZISP_ENT 00004942) (ZISP). **TAJIKISTAN:** Verenkol Lake nr Iskanderkol Lake, 39.08°N 68.36°E, 10 Jul 1947, A. N. Kiritshenko, 1♀ (ZISP_ENT 00004943) (ZISP). **TURKMENISTAN:** Tejen [Tedzhen], 37.378°N 60.509°E, 22 May 1889, Semenov, 1♀ (AMNH_PBI 00338445) (MZH). **UZBEKISTAN:** Termez [Buchara mer. = former Bukhara Chanate], 37.21666°N 67.26666°E, 22 May 1912, A. N. Kiritshenko, 1♀ (ZISP_ENT 00004944) (ZISP).

Specimens of *Stenodema pilosa* previously identified as *S. trispinosa:*

MONGOLIA: Central Aimak: Near Ulaanbaator [Urga], 47.927°N 106.909°E, May 1925 - Jun 1925, Kozlov, 1♀ (ZISP_ENT 00002771) (ZISP); Jun 1925 - Jul 1925, Kozlov, 1♂ (ZISP_ENT 00003631) (ZISP). **Selenge Aimak:** Upper course of Zakhorin Gol River, Men'zya, N Kentey, 49.15°N 108.5°E, 22 Sep 1927, Kondratieva, 4♂ (ZISP_ENT 00003641, ZISP_ENT 00003642) (ZISP). **RUSSIAN FEDERATION: Altai Terr.:** Lebyazh'ye, 51.68333°N 80.83333°E, 26 Jul 1948, Nikolskaya, 1♂ (ZISP_ENT 00003623) (ZISP). **Amur Prov.:** Selemdzhinskiy Dist., Norskiy Nat Res, bank of Burunda River, 3 km upstream from the river mouth, 52.55593°N 130.05855°E, 01 Aug 2006, Veselova & Ryvkin, 1♀ (ZISP_ENT 00013645) (ZISP). Simonovo, 75 km W Svobodnyi, 51.45°N 126.96666°E, 20 Jul 1959, I. M. Kerzhner, 1♂ (ZISP_ENT 00003621) (ZISP). **Irkutsk Prov.:** Bunbuy, 56.38306°N

99.02861°E, 06 Jun 1915, Valdaev, 1♀ (ZISP_ENT 00011830) (ZISP); 06 Jun 1916, Valdaev, 1♀ (ZISP_ENT 00004885), 1♂ (ZISP_ENT 00004889) (ZISP). Irkut river, downstream Ekhe-Ugun [Ekhe-Ukhgun', Ikhe-Ukhgun'], S Irkutsk. prov., 51.65111°N 101.73°E, 01 Aug 1973 - 02 Aug 1973, Gartun, 1♂ (ZISP_ENT 00003628) (ZISP). Irkutsk, 52.31666°N 104.23333°E, 20 May 1915, A. Beloblin, 3♂ (ZISP_ENT 00002704) (ZISP). Melnikovo nr Irkutsk, 52.18333°N 104.1°E, 24 May 1905, Tikhomirov, 1♀ (ZISP_ENT 00004890) (ZISP). **Kamchatka Terr.:** Petropavlovsk-Kamchatskiy, around Abelya St., 53.06621°N 158.60123°E, 09 Aug 2021, A. Namyatova, 1♀ (ZISP_ENT 00005746) (ZISP). **Khanty-Mansi Distr.:** Surgut Dist, nr Yuganskiy Natural Reserve, 60.2°N 74°E, 15 Aug 2002, Ryvkin, 1♂ (ZISP_ENT 00003626), 1♀ (ZISP_ENT 00003626), 1;u (ZISP_ENT 00003626) (ZISP). Ugut, 60.51°N 74.029°E, 05 Aug 2002, Ryvkin, 1♂ (ZISP_ENT 00002805) (ZISP). **Leningrad Prov.:** Lebyazhye, 59.93333°N 29.41666°E, 26 Jul 1948, Nikolskaya, 2♂ (ZISP_ENT 00004883, ZISP_ENT 00004879) (ZISP). Pushkin nr N entrance of All-Russian Institute of Plant Protection, along road, 30.42446°N 59.73837°E, 25 m, 14 Jun 2020, A. Namyatova, 1♀ (ZISP_ENT 00009386) (ZISP). Sergievka, 59.89362°N 29.8369°E, 17 May 2018, A. Namyatova, 1♀ (ZISP_ENT 00003716), 1♂ (ZISP_ENT 00003725) (ZISP). The Scientific Research Institute of Biology, Petergof, 59.89402°N 29.84222°E, 16 Aug 2018, A. Namyatova, 1♀ (ZISP_ENT 00009385) (ZISP). **Murmansk Prov.:** 8 km SSE of Kandalaksha, 67.083°N 32.441°E, 25 Jul 2001, Dmitriev, 1♂ (ZISP_ENT 00003615) (ZISP). **Orenburg Prov.:** Orenburg Nat. Res., Burtinskaya Steppe, 14 km S of Burlykskiy, 51.2289°N 56.6661°E, 30 May 2021, F. Konstantinov & A. Namyatova, 1♀ (ZISP_ENT 00003715) (ZISP). **Primorsky Terr.:** Valley of Odarka River, 25 verst from Evgen'evka Station (Spassk), 44.63333°N 132.91666°E, 18 Jul 1911, Cherskiy, 1♂ (ZISP_ENT 00003624) (ZISP). Vinogradovka, 46.2°N 134.4°E, 28 May 1929, Kiritshenko, 1♀ (ZISP_ENT 00002778) (ZISP); 06 Jul 1929, A. N. Kiritshenko, 2♀ (ZISP_ENT 00004886, ZISP_ENT 00004887) (ZISP); 27 Jul 1929, A. N. Kiritshenko, 1♂ (ZISP_ENT 00003635) (ZISP); 30 Jul 1929, A. N. Kiritshenko, 1♂ (ZISP_ENT 00003622) (ZISP); 04 Aug 1929, A. N. Kiritshenko, 1♂ (ZISP_ENT 00004882) (ZISP); 09 Aug 1929, A. N. Kiritshenko, 3♂ (ZISP_ENT 00002797, ZISP_ENT 00002798, ZISP_ENT 00003632) (ZISP). **Saratov Prov.:** Between Pugachev [Pugachevsk] and Korneevka, Samara Government, 51.9°N 48.75°E, 02 Jul 1925, Dyakonov, 2♂ (ZISP_ENT 00002696, ZISP_ENT 00002696) (ZISP). **Tuva Rep.:** Balgazyn, Slopes of eastern Tannu-Ola, 51°N 95.18333°E, 04 Jun 1948, Cherepanov, 1;u (ZISP_ENT 00011833) (ZISP). Bank of Uvs Nuur [Upsa-Nur?] Lake, 50.61666°N 93°E, 10 Jul 1947, Yakushevich, 1♂ (ZISP_ENT 00003629) (ZISP). Boyarovka, on Kaa-khem river, 51.5333°N 95.4°E, 28 Jun 1949, Perevozchikova, 1♂ (ZISP_ENT 00004880) (ZISP). Khandagayty, NW Uspa-Nur Lake, 50.71666°N 92.03333°E, 10 Jul 1947, Yakushevich, 1♂ (ZISP_ENT 00004881) (ZISP). Sosnovka, Turgen River, Foothills of Tannu-Ola Mts. Range, 51.11666°N 94.5°E, 15 Jun 1949 - 16 Jun 1949, Dyatlova, 2♀ (ZISP_ENT 00002777, ZISP_ENT 00009373) (ZISP). Todzhinskiy Dist., Toora-Khem, 52.47461°N 96.11005°E, 06 Jun 1992, Ryvkin, 1♂ (ZISP_ENT 00002796) (ZISP). Todzhinsky Dist., upper course of Bolshoy Yenisey River, 52.27558°N 96.03384°E, 14 Jun 1992, Ryvkin, 1♂ (ZISP_ENT 00003630), 2♀ (ZISP_ENT 00003630) (ZISP). Turan, basin of Biykhem [Bol'shoy Enisey] River, 52.146°N 93.908°E, 05 Jun 1949, Dyatlova, 1♀ (ZISP_ENT 00002773) (ZISP). **Yakutia Rep.:** Khomurgan Arbyn near estuary of Aldan river, 63.44028°N 129.56194°E, 29 Jun 1926, Bianchi, 1♀ (ZISP_ENT

00004884) (ZISP); 01 Jul 1926, Moskvin, 1♀ (ZISP_ENT 00002780) (ZISP). Left bank of Lena river, 30 verst [32 km] upstream Tebya-Basinskoye [Tyube-Baga, Tyube-Bakha], 63.04528°N 129.54444°E, 09 Jul 1926, L. Bianchi, 1♀ (ZISP_ENT 00002776) (ZISP). Namtsy [Namskoe], left bank of Lena River, 90 verst from Yakutsk, 62.71666°N 129.65°E, 28 Jul 1926, Bianchi, 1♀ (ZISP_ENT 00002779) (ZISP). Nr Yakutsk, 62.01666°N 129.71666°E, 19 Jun 1926, Expedition of Yakut Museum, 1♀ (ZISP_ENT 00004888) (ZISP); 03 Aug 2008, A. Ovchinnikov, 1♂ (ZISP_ENT 00013639) (ZISP). Sergelyakh cottage vill.,, 61.9906°N 129.6153°E, 06 Jun 1926, Gileva, 1♀ (ZISP_ENT 00009371) (ZISP). Tyllymin Vtoroy [2 Tyylyminskiy Nasleg], 61.55°N 129.93333°E, 02 Jul 1925, Bianchi, 1♀ (ZISP_ENT 00002732) (ZISP). Verkhoyanskiy Dist., Yana River, nr Stolby, 63.00225°N 129.53356°E, 29 Jul 2008, A. Ovchinnikov, 1♂ (ZISP_ENT 00013640) (ZISP). Yakutsk, 62.0339°N 129.7331°E, 15 Jun 1927, Nikiforov, 1♀ (ZISP_ENT 00002775) (ZISP). Zhigansk (on Lena River), 66.76666°N 123.35°E, 08 Jun 1928, Nikiforov, 1♀ (ZISP_ENT 00004878) (ZISP). **Zabaikalsky Terr.:** Chita, 52.03333°N 113.45°E, 10 Jun 1912, Gitelman, 1♀ (ZISP_ENT 00009372) (ZISP). Sretensk, 52.23333°N 117.68333°E, 19 Jun 1928, Kapustin, 1♀ (ZISP_ENT 00002774) (ZISP). **TAJIKISTAN:** Sary-Tag River nr Iskanderkul Lake, 39.05°N 68.33333°E, 25 Jul 1947, A. N. Kiritshenko, 1;u (ZISP_ENT 00002709) (ZISP). **USA: West Virginia:** ***Marion Co.:*** Fairmont, 39.48508°N 80.14258°W, 304 m, 15 Jul 1930, Musgrave, 1♀ (ZISP_ENT 00002772), 1♂ (ZISP_ENT 00003633) (ZISP).

Stenodema rubrinervis

JAPAN: Honshu: ***Tochigi Pref. Co.:*** Nikko, Yumoto, 36.81, 139.42, 36.81°N 139.42°E, 02 Sep 1999 - 03 Sep 1999, Belokobylskij, 1♂xd (ZISP_ENT 00013688) (ZISP). **Kyushu (Island):** ***Fukuoka Pref. Co.:*** Fukuoka, 33.58736°N 130.37881°E, 6 m, 18 Aug 1999, Belokobylskij, 1♂ (ZISP_ENT 00003728) (ZISP). ***unknown Co.:*** Kunitachi, 35.66666°N 139.43333°E, 16 Jun 1936, Zhenzhurist, 1♂ (ZISP_ENT 00013686), 1♀ (ZISP_ENT 00013685) (ZISP). [Takao-san, Honshiu], 1700, Zhenzhurist, 1♀ (ZISP_ENT 00004957) (ZISP). **KOREA: South Korea:** Gyongsang-namdo Prov, 30 km NNW of Jinju, 35.41666°N 127.93333°E, 800 m, 12 Jun 2002, Belokobylskij, 1♀ (ZISP_ENT 00004959), 2♂ (ZISP_ENT 00004956, ZISP_ENT 00004941) (ZISP); 16 Jun 2002, Belokobylskij, 1♂ (ZISP_ENT 00013687) (ZISP). Gyongsang-namdo Prov., 60 km SW of Pursan, Geoje Do, 34.8547°N 128.6392°E, 23 Jun 2002, Belokobylskij, 1♀ (ZISP_ENT 00004960) (ZISP). Seoul, 37.562°N 126.991°E, 31 Jul 1938, Zhenzhurist, 2♀ (ZISP_ENT 00013689, ZISP_ENT 00003772) (ZISP).

Stenodema sibirica

KOREA: North Korea: Sinmusan, Khamgen Pukdo, 40.04111°N 125.31583°E, 18 Aug 1950, Borkhsenius, 1♀ (ZISP_ENT 00003694), 1♂ (ZISP_ENT 00003703) (ZISP). **MONGOLIA: Central Aimak:** At the confluence of Zakhorin-gol and Men'zya Rivers, 49.143°N 108.517°E, 19 Sep 1927, Kondratieva, 1♀ (ZISP_ENT 00003697), 1♂ (ZISP_ENT 00003709) (ZISP). Sutszukte, NW of

Uaalanbaatar [Urga], SE Kentey, 06 Aug 1924 - 10 Aug 1924, Kozlov, 1♂ (ZISP_ENT 00011824) (ZISP); 11 May 1925 - 31 May 1925, Kozlov, 2♂ (ZISP_ENT 00003707, ZISP_ENT 00003706) (ZISP); 05 Jun 1925 - 14 Jun 1925, Kozlov, 1♀ (ZISP_ENT 00004930) (ZISP); 14 Jun 1925 - 22 Jun 1925, Kozlov, 1♀ (ZISP_ENT 00003696) (ZISP). **Hentiy Aimak:** Breven-khit, SE Hentiy, 48.2°N 109.4333°E, 10 Aug 1926, Kondratieva, 1♀ (ZISP_ENT 00004939) (ZISP). Onon River, middle current of Tarasun River, 49°N 109.2°E, 04 Sep 1926, Kondratieva, 2♂ (ZISP_ENT 00003702, ZISP_ENT 00003689) (ZISP). [Middle flow of Zakhorin-gol, Men'zya River, N of Khentey [Kentey]], 17 Sep 1927 - 18 Sep 1927, Kondratieva, 1♀ (ZISP_ENT 00004929) (ZISP). **RUSSIAN FEDERATION: Altai Rep.:** Biya River, 2 km downstream from Artybash, 16 Jul 2006, A. Ovchinnikov, 1♀ (ZISP_ENT 00013642) (ZISP). Chemal [former Biysk Uezd, Tomsk Government], 51.4°N 86.01666°E, 19 Jul 2007, Belokobylskij, 1♀ (ZISP_ENT 00013638) (ZISP). Chettu Mt, N of Teletskoe Lake, 51.778°N 87.368°E, 21 May 1901, Ignatov, 2♂ (ZISP_ENT 00004917, ZISP_ENT 00011823), 1♀ (ZISP_ENT 00003692) (ZISP). Dzhaylau Tract, N bank of Teletskoe Lake, 51.779°N 87.353°E, 25 May 1901, Ignatov, 1♂ (ZISP_ENT 00002693) (ZISP). [Chilishch River, nr Teletskoe Lake], 25 Jun 1909, A. F. Emeljanov, 1♂ (ZISP_ENT 00011828) (ZISP). **Altai Terr.:** Bele, E bank of Teletskoye Lake, 51.43333°N 87.76667°E, 01 Aug 1901, Ignatov, 1♂ (ZISP_ENT 00011825) (ZISP). **Irkutsk Prov.:** 93 km E Tayshet, 55.93333°N 99.46666°E, 12 May 1941, Barovsky, 1♀ (ZISP_ENT 00003693) (ZISP). Bolshie Koty, SW Bank of Baykal Lake, 51.9°N 105.05°E, 10 Aug 1950, A. N. Kiritshenko, 1♀ (ZISP_ENT 00004928) (ZISP). Irkutsk, 52.31666°N 104.23333°E, Yakovlev, 1♂ (ZISP_ENT 00004918) (ZISP). Maritui, Baikal Lake, 51.79028°N 104.21833°E, 508 m, 11 Jun 1909, Shubert, 1♂ (ZISP_ENT 00003699) (ZISP). **Khabarovsk Terr.:** Khungari, Voznesenskiy Distr., 50.4°N 138.16444°E, 10 Jul 1910 - 13 Jul 1910, Soldatov, 1♂ (ZISP_ENT 00003710) (ZISP). Srednetambosvkoe, lower course of Amur River, 50.78959°N 137.88854°E, 18 Jul 1910, Soldatov, 1♂ (ZISP_ENT 00003711) (ZISP). Verkhebureinskiy Dist., Bureinskiy Nat Res., nr Strelka Cordon, 51.64894°N 134.266°E, 13 Sep 2006, Ryvkin, 2♂ (ZISP_ENT 00013646) (ZISP). **Khakassia Rep.:** Sayanogorsk [Oznachennaya] on Yenisey River, 53.08333°N 91.38333°E, 09 Sep 1930, F.K. Lukjanovitsh, 1♂ (ZISP_ENT 00003617) (ZISP). **Khanty-Mansi Distr.:** Surgut Dist, nr Yuganskiy Natural Reserve, 60.2°N 74°E, 20 Sep 2002, Ryvkin, 1♂ (ZISP_ENT 00003701) (ZISP). **Krasnoyarsk Terr.:** Krasnoyarsk, 56.00972°N 92.79167°E, 28 May 1905, Anuchina, 1♂ (ZISP_ENT 00013632) (ZISP). **Primorsky Terr.:** Gaydamak Bay, 42.87333°N 132.69522°E, 21 May 1906, Shmidt, 1♀ (ZISP_ENT 00009383) (ZISP). Middle stream of Suputinka River, tributary of Suyfun River, 44.26528°N 131.69194°E, 10 Oct 1936, Kurentsov, 1♂ (ZISP_ENT 00003708) (ZISP). Oblachnaya Mt, 43.689°N 134.198°E, 02 Jul 1962, Kovalev, 1♂ (ZISP_ENT 00003705) (ZISP). Pos'yet, Ussuriysk Prov., 42.63333°N 130.78333°E, 30 Jul 1915, Kuznetsov, 1♂ (ZISP_ENT 00011821) (ZISP). Sedanka nr Vladivostok, 43.21806°N 131.95194°E, 18 Sep 1932, Rysakov, 1♀ (ZISP_ENT 00003700) (ZISP); 20 Sep 1932, Rysakov, 1♂ (ZISP_ENT 00003712) (ZISP). S of Oblachnaya Mt., 43.689°N 134.198°E, 08 Aug 1963, I. M. Kerzhner, 1♀ (ZISP_ENT 00003695) (ZISP). Vinogradovka, 46.2°N 134.4°E, 24 Jul 1929, A. N. Kiritshenko, 1♀ (ZISP_ENT 00003688) (ZISP); 25 Jul 1929, A. N. Kiritshenko, 1♂ (ZISP_ENT 00003713) (ZISP); 28 Jul 1929, A. N. Kiritshenko, 2♂ (ZISP_ENT 00004919, ZISP_ENT 00011827) (ZISP); 30 Jul 1929, A. N. Kiritshenko, 1♂ (ZISP_ENT 00004940) (ZISP). Vladivostok, 43.11666°N

131.9°E, 01 Aug 1909, Berger, 1♂ (ZISP_ENT 00011822) (ZISP). **Sakhalin Prov.:** Novoaleksandrovsk, South Sakhalin, 47°N 142.7°E, 10 Aug 1973, Kuporosov, 2♂ (ZISP_ENT 00003704) (ZISP); 20 Aug 1973, Ermolenko, 1♀ (ZISP_ENT 00009384) (ZISP); 07 Sep 1973, I. M. Kerzhner, 1♂ (ZISP_ENT 00003698), 1♀ (ZISP_ENT 00003698) (ZISP). [from station Petropavlovka to station Vladimirovka], 03 Jul 1901, Schmidt, 1♀ (ZISP_ENT 00003691) (ZISP).

Stenodema turanica

AZERBAIJAN: Nakhichevan Prov.: Darasham II Railway Station on Araks River, 38.97778°N 45.5°E, 11 Oct 1931 - 12 Oct 1931, Ryabov, 2♂ (ZISP_ENT 00004931, ZISP_ENT 00004933) (ZISP). Dzhuga nr Culfa [Dzhulfa], 38.96666°N 45.6°E, 13 Aug 1931 - 14 Aug 1931, Ryabov, 1♀ (ZISP_ENT 00004952) (ZISP); 10 Sep 1931, Ryabov, 1♂ (ZISP_ENT 00003655) (ZISP). Tatoni, Zuvant, Talysh, 38.65°N 48.4°E, 05 Aug 1933 - 06 Aug 1933, Ryabov, 1♀ (ZISP_ENT 00004953) (ZISP). **CHINA: Qinghai:** Xining [Sinin], N Nanshan, 36.6188°N 101.75022°E, 29 Jul 1908, Kozlov, 1♀ (ZISP_ENT 00003685) (ZISP). **IRAN (ISLAMIC REPUBLIC OF): Fars:** Abadeh, 31.16449°N 52.63344°E, 10 Jul 1955, Steinberg, 1♂ (ZISP_ENT 00004937) (ZISP). **Kerman:** Kerman, 30.26666°N 57.05°E, May 1928, Siyazov, 3♂ (ZISP_ENT 00003618, ZISP_ENT 00003664, ZISP_ENT 00003681) (ZISP). **KYRGYZSTAN:** Ak-terek, 5 km N Gava, 41.3°N 72.81666°E, 19 Jul 1937, A. N. Kiritshenko, 1♀ (ZISP_ENT 00003680) (ZISP). Kenkol Pass, W Fergana Mts. Range, 41.56°N 73.08°E, 16 Jul 1930, Bianchi, 1♂ (ZISP_ENT 00004932) (ZISP). Kyrgyz [Aleksandr] Mts. Range, Alamedyn River, 42.85°N 74.56666°E, 2624 m, 02 Jul 1910, A. Kiritshenko, 1♀ (ZISP_ENT 00002788) (ZISP). mt Takyr-Ter, Kyrgyz [Aleksandr] Mts Range, Bishkek distr, 42.29139°N 78.58167°E, 19 Jul 1913 - 20 Jul 1913, Chernavskiy, 1♀ (ZISP_ENT 00004936) (ZISP). **MONGOLIA: Bayan Olgiy Aimak:** Ikh-Dzhargalantyn-Gol River, 20 km NW Burenhayrhan [Bulgan], 46.18333°N 91.31666°E, 05 Jul 1980, I. M. Kerzhner, 1♀ (ZISP_ENT 00003687), 1♂ (ZISP_ENT 00003687) (ZISP). **RUSSIAN FEDERATION: Buryatia Rep.:** Kyakhta [former Troitskosavsk], 50.3508°N 106.44939°E, 757 m, 25 May 1928, F.K. Lukjanovitsh, 1♀ (ZISP_ENT 00002785) (ZISP). **Dagestan Rep.:** Okyuztau Mt. Gimrinskiy Mts. Range, 42.68333°N 47.01666°E, 22 Aug 1944, Ryabov, 1♀ (ZISP_ENT 00003683) (ZISP). **Irkutsk Prov.:** Belaya River, tributary of Angara River, S of Irkutsk government, 52.91722°N 103.65472°E, Gartung, 1♀ (ZISP_ENT 00002784) (ZISP). **Tuva Rep.:** 25-30 km W Kyzyl, 51.70587°N 93.94756°E, 04 Jun 1962, M. Baytenov, 1♂ (ZISP_ENT 00004901) (ZISP). Bank of Uvs Nuur [Upsa-Nur?] Lake, 50.61666°N 93°E, 23 Jul 2009 - 24 Jul 2009, Belokobylskij, 1♀ (ZISP_ENT 00013635) (ZISP). **TAJIKISTAN:** Between Kvak and Kondara, Valley of Varzab River, 38.83°N 68.83°E, 08 Jun 1943, A. N. Kiritshenko, 1♂ (ZISP_ENT 00003656) (ZISP). Dushanbe [former Stalinabad], 38.56666°N 68.76666°E, 29 May 1934, Gussakovskiy, 1♂ (ZISP_ENT 00004938) (ZISP); 14 Jul 1943 - 15 Jul 1943, Gussakovskiy, 1♀ (ZISP_ENT 00002794) (ZISP). Gissar Mts. Range, NW bank of Iskanderkul Lake, 39.08333°N 68.35°E, 12 Jul 1947, A. N. Kiritshenko, 1♂ (ZISP_ENT 00003663), 1♀ (ZISP_ENT 00003686) (ZISP). Khozor-mecho River nr Iskanderkul Lake, 39.05°N 68.36666°E, 28 Jul 1947, A. N. Kiritshenko, 1♀ (ZISP_ENT 00002735) (ZISP). Kondara Canyon,

Varzob [Varzoba] village, 38.7758°N 68.8308°E, 1000 m, 22 Aug 1945, Gussakovskiy, 1♂ (ZISP_ENT 00003661), 1♀ (ZISP_ENT 00004935) (ZISP). Sary-Tag River nr Iskanderkul Lake, 39.05°N 68.33333°E, 21 Jul 1947, A. N. Kiritshenko, 1♀ (ZISP_ENT 00004934) (ZISP); 25 Jul 1947, A. N. Kiritshenko, 1♀ (ZISP_ENT 00003682) (ZISP). Vorukh, 39.85°N 70.55°E, 01 Jul 1908, Zarudny, 1♂ (ZISP_ENT 00003659) (ZISP). Voznaut, Roshan, Pamiry, 37.95°N 71.2833°E, 28 Jul 1915, Lazdin, 1♂ (ZISP_ENT 00003657) (ZISP). [Chash, S slopes of Gissarskiy Mt Range], 10 Aug 1929, E. Kuznetsova, 1♂ (ZISP_ENT 00003662) (ZISP). **TURKMENISTAN:** Bosaga on Amu-Darya River nr Kelif, 37.53333°N 65.66666°E, 30 Oct 1931, Ushinskiy, 1♀ (ZISP_ENT 00002793) (ZISP). **UKRAINE:** [Korzhovka, Shostka Dist., Sumskaya Government], 05 May 1940, Kurnakov, 1♀ (ZISP_ENT 00011832) (ZISP). **UZBEKISTAN: Samarqand:** Jomboy on Zeravshan River, 39.69015°N 67.07685°E, 31 Jul 1929, B. Prikhodko, 1♂ (ZISP_ENT 00003660) (ZISP). Khavast [former st. Kaufmanskaya], 40.212°N 68.841°E, 19 Jul 1908 - 21 Jul 1908, Zarudny, 1♂ (ZISP_ENT 00003658) (ZISP). Shakhimardan, Alayskiy Mts. Range, 39.96666°N 71.78333°E, 05 Jun 1900, Germs, 1♂ (ZISP_ENT 00003654) (ZISP). Syr-Darya river, Chimgan [Tshimgan], 41.55°N 70.01666°E, 13 Jul 1913, E. Kiritshenko, 1♀ (ZISP_ENT 00003684) (ZISP). Termez [Buchara mer. = former Bukhara Chanate], 37.21666°N 67.26666°E, 30 Jun 1912, A. N. Kiritshenko, 1♂ (ZISP_ENT 00002700) (ZISP).

Stenodema virens

ARMENIA: Ararat [Davalu], Valley of Araks River, 39.83333°N 44.68333°E, 1910, Ksenzhopol'skiy, 1♂ (ZISP_ENT 00003644) (ZISP). **GEORGIA:** Tilinbuli, 41.53333°N 43.63333°E, 2500 m, 1700, V. Lukhtanov, 1♀ (ZISP_ENT 00013661) (ZISP). **KAZAKHSTAN: Almaty Prov.:** Topolevka, E of Sarkand, Dzhungar Alatau, 45.4°N 80.3333°E, 1145 m, 07 Jul 1957, I. M. Kerzhner, 1♀ (ZISP_ENT 00004893) (ZISP). Valley of Bol'shaya Almatinka [Almatinka] River, 47.57°N 76.9°E, 06 Sep 1933, Shnitnikov, 1♀ (ZISP_ENT 00002795) (ZISP). **MONGOLIA: Hentiy Aimak:** Onon River, middle current of Tarasun River, 49°N 109.2°E, 31 Aug 1926, Kondratieva, 5♂ (ZISP_ENT 00004900, ZISP_ENT 00003616, ZISP_ENT 00003616, ZISP_ENT 00002701, ZISP_ENT 00002702), 2♀ (ZISP_ENT 00004902, ZISP_ENT 00002787) (ZISP). Valley of Zane river, system of Toola river, SW Hentiy, 10 Aug 1927, Kondratieva, 1♂ (ZISP_ENT 00003645) (ZISP). **Selenge Aimak:** Upper course of Zakhorin Gol River, Men'zya, N Kentey, 49.15°N 108.5°E, 11 Sep 1927, Kondratieva, 3♂ (ZISP_ENT 00004898, ZISP_ENT 00003651, ZISP_ENT 00003647) (ZISP). **RUSSIAN FEDERATION: Buryatia Rep.:** Kyakhta [former Troitskosavsk], 50.3508°N 106.44939°E, 757 m, 03 May 1928, F.K. Lukjanovitsh, 1♀ (ZISP_ENT 00004895) (ZISP). Ust'-Kiran, Kornakovskiy Forest, 50.66666°N 107.3°E, 03 Aug 1908, Khomze, 1♂ (ZISP_ENT 00004896) (ZISP). **Irkutsk Prov.:** Bunbuy, 56.38306°N 99.02861°E, 08 Jun 1915, Valdaev, 1♂ (ZISP_ENT 00003646) (ZISP). Kuzmikha nr Irkutsk, 52.21666°N 104.26666°E, 16 Jun 1924, Vinogradov, 2♂ (ZISP_ENT 00004899, ZISP_ENT 00003643) (ZISP). Ol'khon Is, forest ap. 2.5 km SE from Khuzhir nr Tumyr-Tologoy Stream, 53.17037°N 107.35644°E, 535 m, 19 Aug 2020, A. Namyatova, 1♂ (ZISP_ENT 00003724) (ZISP). Yurty, 56.03333°N 97.61666°E, 26 May 1911, Mishin, Verkhovskiy, 1♀ (ZISP_ENT

00002791) (ZISP). **Karachay-Cherkessia Rep.:** Teberda Nature Reserve, gorge of the Dzhemagat River, southern slopes of Kendellyar-Lyar mountain, 43.47861°N 41.87911°E, 1520 m, 26 Jul 2021, Golub, 1♂ (ZISP_ENT 00013665) (ZISP). **Krasnoyarsk Terr.:** Bogotol on Chulym River, Western Siberia, 56.15°N 89.56666°E, 08 Jun 1936, F.K. Lukjanovitsh, 1♀ (ZISP_ENT 00002733) (ZISP). Kanygino, 53.686°N 92.053°E, 16 Jul 1925, Serebrennikov, 1♀ (ZISP_ENT 00004913) (ZISP). Sukhobuzimskoe (Sukhoy Buzim), 56.50111°N 93.27138°E, 17 Jun 1903, Salstrem, 1♂ (ZISP_ENT 00003650) (ZISP). Taseevo, Kansk Distr., 57.209°N 94.898°E, 23 Jun 1914, Varaksina, 1♀ (ZISP_ENT 00011836) (ZISP). **Leningrad Prov.:** Gorelovo, 59.76666°N 30.1°E, 29 May 1898 - 07 Jun 1898, Novotortsev, 1♂ (ZISP_ENT 00003652) (ZISP). Kharlamova Gora, 58.97361°N 29.32972°E, 10 Jul 1896, Bikhner, 1♀ (ZISP_ENT 00004894) (ZISP); 31 Jul 1898, Bikhner, 1♀ (ZISP_ENT 00002790) (ZISP). Krupeli, 58.8°N 29.949°E, 21 Jun 1898, Mazarakiy, 1♂ (ZISP_ENT 00003648) (ZISP). Lopukhinka, 59.733°N 29.401°E, 03 Aug 1894, Bianchi, 2♀ (ZISP_ENT 00002789, ZISP_ENT 00002786) (ZISP); 19 Aug 1894, Bianchi, 1♂ (ZISP_ENT 00004897) (ZISP); 23 Aug 1894, Bianchi, 1♂ (ZISP_ENT 00003653) (ZISP). **Tver Prov.:** Bologoe, 57.86666°N 34.05°E, 19 May 1903, Collector unknown, 1♂ (ZISP_ENT 00003649) (ZISP). **UKRAINE:** Desnyanskoe vill. [Psarovka, Psarevka, Sverdlovka], Krolevetskiy uezd, Chernigov prov,, 51.79°N 33.04°E, 29 Jul 1916, A.A. Stackelberg, 1♀ (ZISP_ENT 00002782) (ZISP). Slavuta, 50.28333°N 26.86666°E, 08 Jul 1911, F. A. Zaytsev, 1♀ (ZISP_ENT 00002792) (ZISP). [Korzhovka, Shostka Dist., Sumskaya Government], 04 May 1940, Kurnakov, 1♀ (ZISP_ENT 00004914) (ZISP). **UZBEKISTAN**: Samarkand, 38.56666°N 68.03333°E, no date provided, Fedchenko coll., Syntype of Miris virens var. testaceus Reuter, 1875, 1♂ (AMNH_PBI 00345034) (MZH).
