# Supplementary material for: ﻿Delimitation of the widely distributed Palearctic Stenodema species (Hemiptera, Heteroptera, Miridae): insights from molecular and morphological data
Source: Zookeys. 2024 Aug 13;1209:245–94. doi: 10.3897/zookeys.1209.124766 (PMC11336388; doi:10.3897/zookeys.1209.124766)
Supplement: Supplementary material 2 — Voucher information for the specimens used for the comparison and phylogenies of the Palearctic species [file zookeys-1209-245_article-124766__-s002.pdf]

Voucher information for the specimens used for the comparison and phylogenies of the Palearctic species

Species	Voucher	COI Genbank accession number	16S Genbank accession number	Country	USI number
Leptopterna sp.	ST45	PP273511	PP273719	Russia/ Orenburg Prov.	ZISP_ENT 00013657
Trigonotylus sp.	ST41	PP273512	PP273737	Russia/ Kalmykia Rep.	ZISP_ENT 00013652
Stenodema calcarata	ST31	PP273513	PP273744	Russia/ Primorskiy Terr.	ZISP_ENT 00013641
Stenodema calcarata	ST16	PP273514	PP273734	Russia/ Altay Prov.	ZISP_ENT 00003719
Stenodema calcarata	ST35			Russia/ Amur Prov.	ZISP_ENT 00013645
Stenodema calcarata	ST04	PP273515	PP273720	Russia/ Bashkortostan Rep	ZISP_ENT 00013651
Stenodema calcarata	ST37	PP273516	PP273747	Russia/ Belgorod Prov	ZISP_ENT 00013660
Stenodema calcarata	ST06	PP273518	PP273726	Russia/ Leningrad Prov.	ZISP_ENT 00013662
Stenodema calcarata	ST02	PP273519	PP273721	Russia/ Murmansk Prov.	ZISP_ENT 00013658
Stenodema calcarata	ST17		PP273735	Russia/Stavropol Prov.	ZISP_ENT 00003720
Stenodema calcarata	ST14	PP273520	PP273733	Russia/ Ulyanovsk Prov.	ZISP_ENT 00003717
Stenodema calcarata	ST26	PP273521	PP273740	Turkey	ZISP_ENT 00013633
Stenodema calcarata	28IRAN	PP273522		Iran	
Stenodema calcarata	ST07	PP273523	PP273727	Georgia	ZISP_ENT 00013647
Stenodema calcarata	ST43	PP273517	PP273751	France	ZISP_ENT 00013653
Stenodema calcarata		KM022989		Germany	
Stenodema calcarata		KM022595		Germany	
Stenodema calcarata		KM021949		Germany	
Stenodema calcarata		KM021898		Germany.	
Stenodema calcarata		KM021515		Germany.	
Stenodema calcarata		KM023021		Germany.	
Stenodema calcarata		KM023068		Germany	
Stenodema calcarata		KM021960		Germany	
Stenodema calcarata		KM022869		Germany	
Stenodema calcarata		OL663374		South Korea	
Stenodema calcarata		OL663375		South Korea	
Stenodema calcarata		OL663376		South Korea	
Stenodema calcarata			MF667329	South Korea	
Stenodema calcarata		MZ631685		Finland	

Stenodema calcarata		MZ656495		Finland	
Stenodema calcarata		MZ656669		Finland.	
Stenodema holsata	ST09	PP273524	PP273729	Russia/ Karachay-Cherkessia Rep.	ZISP_ENT 00013664
Stenodema holsata	ST01	PP273525	PP273722	Russia/ Murmansk Prov.	ZISP_ENT 00013663
Stenodema holsata	ST46	PP273526	PP273752	Russia/ Murmansk Prov.	ZISP_ENT 00013654
Stenodema holsata	ST03	PP273527	PP273723	Norway	ZISP_ENT 00013667
Stenodema holsata		KM022857		Germany	
Stenodema holsata		KM022402		Germany	
Stenodema holsata		KM021829		Germany	
Stenodema holsata		KM021735		Germany	
Stenodema holsata		KJ541522		France	
Stenodema holsata		KJ541526		France	
Stenodema holsata		KJ541527		France	
Stenodema holsata		KJ541528		France.	
Stenodema holsata		KM021489		Germany	
Stenodema holsata		KM022581		Germany	
Stenodema holsata		KM287254		Norway	
Stenodema holsata		KJ541538		France	
Stenodema holsata		KJ541590		France	
Stenodema holsata		KJ541604		France	
Stenodema holsata		MZ633438		Finland	
Stenodema holsata		MZ632032		Finland	
Stenodema holsata		MZ632415		Finland	
Stenodema laevigata	ST11	PP273528	PP273730	Russia/Bryansk Prov.	ZISP_ENT 00013666
Stenodema laevigata	ST38	PP273529	PP273748	Russia/Belgorod Prov.	ZISP_ENT 00013659
Stenodema laevigata	ST08	PP273530	PP273728	Crimea Rep.	ZISP_ENT 00013648
Stenodema laevigata	ST39	PP273532	PP273749	Russia/Saratov Prov.	ZISP_ENT 00013649
Stenodema laevigata	ST25	PP273533	PP273725	Russia/Ulyanovsk Prov.	ZISP_ENT 00013634
Stenodema laevigata	ST05	PP273534	PP273724	Russia/Voronezh Prov.	ZISP_ENT 00013656
Stenodema laevigata	ST42		PP273750	Greece	ZISP_ENT 00013650
Stenodema laevigata	01IRAN		PP273753	Iran	
Stenodema laevigata	15IRAN	PP273535	PP273754	Iran	

Stenodema laevigata		KM022395		Germany	
Stenodema laevigata		KM022947		Germany	
Stenodema laevigata		KM021476		Germany	
Stenodema laevigata		KM021700		Germany	
Stenodema laevigata		KM021771		Germany	
Stenodema laevigata		KM022371		Germany	
Stenodema laevigata		KJ541531		France	
Stenodema laevigata		KJ541532		France	
Stenodema laevigata		KM023087		Germany	
Stenodema laevigata		KM022751		Germany	
Stenodema laevigata		KM022142		Germany	
Stenodema laevigata		MW535964		Portugal	
Stenodema laevigata		MW536092		Portugal	
Stenodema laevigata		KJ541537		France	
Stenodema laevigata		KJ541577		France	
Stenodema laevigata		MZ630187		Finland	
Stenodema laevigata		MZ629499		Finland	
Stenodema laevigata	ST24		PP273739	Russia/Pskov Prov.	ZISP_ENT 00003727
Stenodema pilosipes		KR581562		Canada/Alberta	
Stenodema pilosipes		KR032850		Canada/British Columbia	
Stenodema rubrinervis		GU194832	GU194603	South Korea	
Stenodema rubrinervis		KY367176		South Korea	
Stenodema rubrinervis		KY367178		South Korea	
Stenodema rubrinervis		GQ292099		South Korea	
Stenodema rubrinervis		KY367177		South Korea	
Stenodema rubrinervis			MF667330	South Korea	
Stenodema sericans		KM022559		Germany	
Stenodema sericans		KJ541615		France	
Stenodema sericans		KJ541632		France	
Stenodema sibirica	ST36	PP273537	PP273746	Russia/Khabarovsk Terr.	ZISP_ENT 00013646
Stenodema sibirica	ST28	PP273536	PP273743	Russia/Altay Rep.	ZISP_ENT 00013638
Stenodema sibirica	ST32	PP273538	PP273745	Russia/Altay Rep.	ZISP_ENT 00013642

Stenodema sibirica		GU194833	GU194604	South Korea	
Stenodema sibirica		KY367180		South Korea	
Stenodema sibirica		GQ292111		South Korea	
Stenodema sibirica		KY367179		South Korea	
Stenodema sibirica			MF667331	South Korea	
Stenodema trispinosa	ST12	PP273539	PP273732	Russia/Orenburg Prov.	ZISP_ENT 00003715
Stenodema trispinosa	ST29		PP273742	Russia/Yakutia Rep	ZISP_ENT 00013639
Stenodema trispinosa	ST30	PP273540	PP273736	Russia/Yakutia Rep.	ZISP_ENT 00013640
Stenodema trispinosa		MZ656655		Finland	
Stenodema trispinosa		MZ660633		Finland	
Stenodema trispinosa		KR581161		Canada	
Stenodema trispinosa		KR032213		Canada	
Stenodema trispinosa		KR034997		Canada	
Stenodema trispinosa		MF937672		Canada	
Stenodema trispinosa		MF938746		Canada	
Stenodema trispinosa		MF930501		Canada	
Stenodema trispinosa		KR574809		Canada	
Stenodema trispinosa		MF937040		Canada	
Stenodema trispinosa		KR573601		Canada	
Stenodema trispinosa		KR575702		Canada	
Stenodema trispinosa		KU876242		USA/Alaska	
Stenodema trispinosa		KR582246		Canada	
Stenodema trispinosa		MG164925		Canada	
Stenodema_turanica	53IRAN	PP273541		Iran	
Stenodema_turanica	54IRAN	PP273542		Iran	
Stenodema_turanica	ST27	PP273543	PP273741	Russia/Tyva Rep.	ZISP_ENT 00013635
Stenodema vicina		KR034171		Canada/Nova Scotia	
Stenodema vicina		KR575728		Canada/Ontario	
Stenodema vicina		KR583053		Canada/Ontario	
Stenodema vicina		KR574511		Canada/Ontario	
Stenodema vicina		MF936192		Canada/British Columbia	

Stenodema virens	ST10	PP273544	PP273731	Russia/ Karachay-Cherkessia Rep.	ZISP_ENT 00013665
Stenodema virens	ST21	PP273545	PP273738	Russia/ Irkutsk Prov.	ZISP_ENT 00003724
Stenodema virens	ST40	PP273546		Georgia	ZISP_ENT 00013661
Stenodema virens		MZ657175		Finland	

References

Namyatova, A. A., Schwartz, M. D., & Cassis, G. (2021). Determining the position of Diomocoris, Micromimetus and Taylorilygus in the *Lygus*-complex based on molecular data and first records of Diomocoris and Micromimetus from Australia, including four new species (Insecta: Hemiptera: Miridae: Mirinae). *Invertebrate Systematics*, 35(1), 90-131.

Roehrdanz, R., Cameron, S. L., Toutges, M., & Wichmann, S. S. (2016). The complete mitochondrial genome of the tarnished plant bug, Lygus lineolaris (Heteroptera: Miridae). *Mitochondrial DNA Part A*, *27*(1), 48-49.

Wang, P., Li, H., Wang, Y., Zhang, J. H., Dai, X., Chang, J., ... & Cai, W. Z. (2014). The mitochondrial genome of the plant bug Apolygus lucorum (Hemiptera: Miridae): presently known as the smallest in Heteroptera. *Insect science*, *21*(2), 159-173.
