# Supplementary material for: ﻿Delimitation of the widely distributed Palearctic Stenodema species (Hemiptera, Heteroptera, Miridae): insights from molecular and morphological data
Source: Zookeys. 2024 Aug 13;1209:245–94. doi: 10.3897/zookeys.1209.124766 (PMC11336388; doi:10.3897/zookeys.1209.124766)

COI-based RaxML tree for full datset (124 terminals)

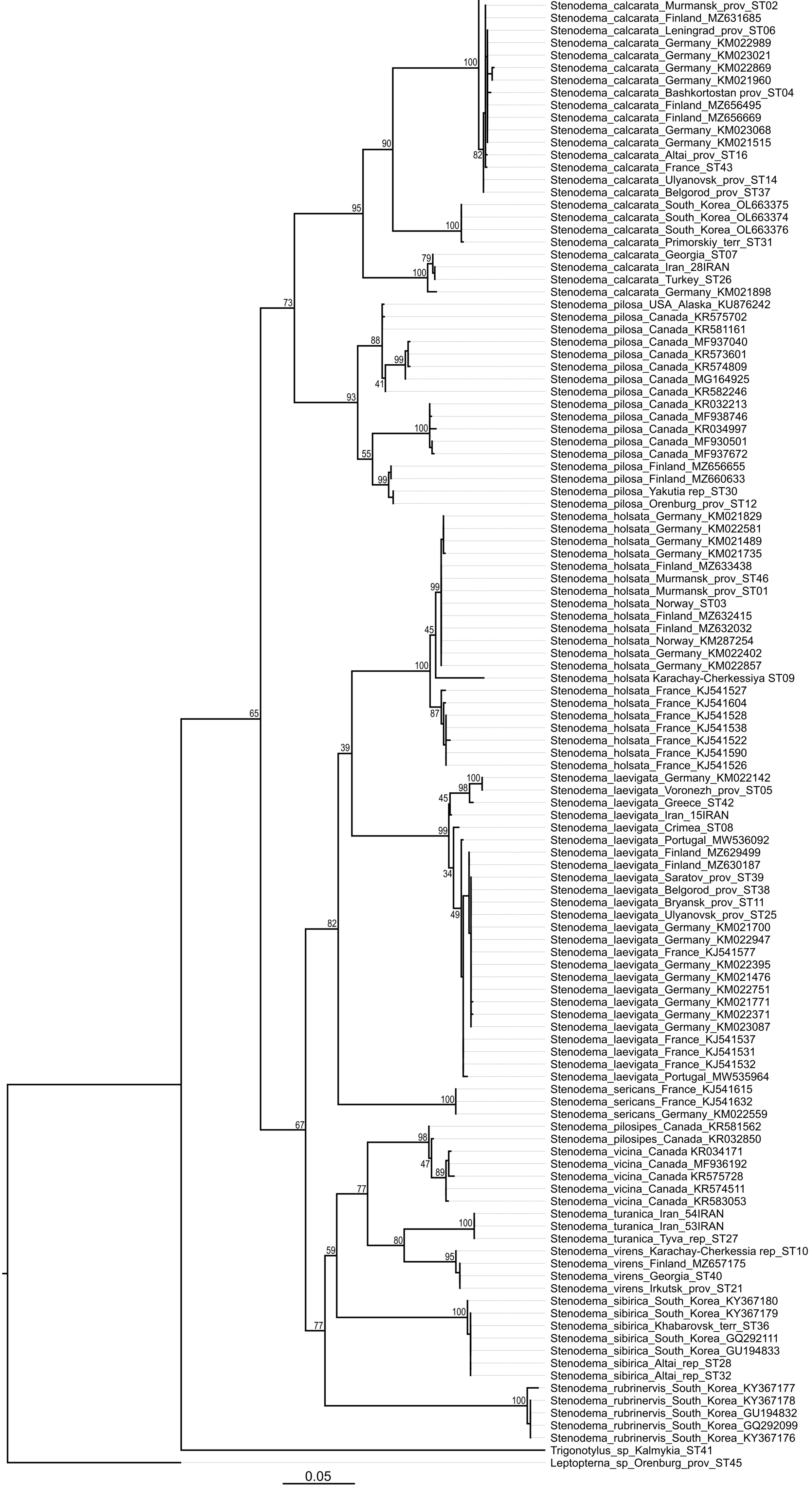

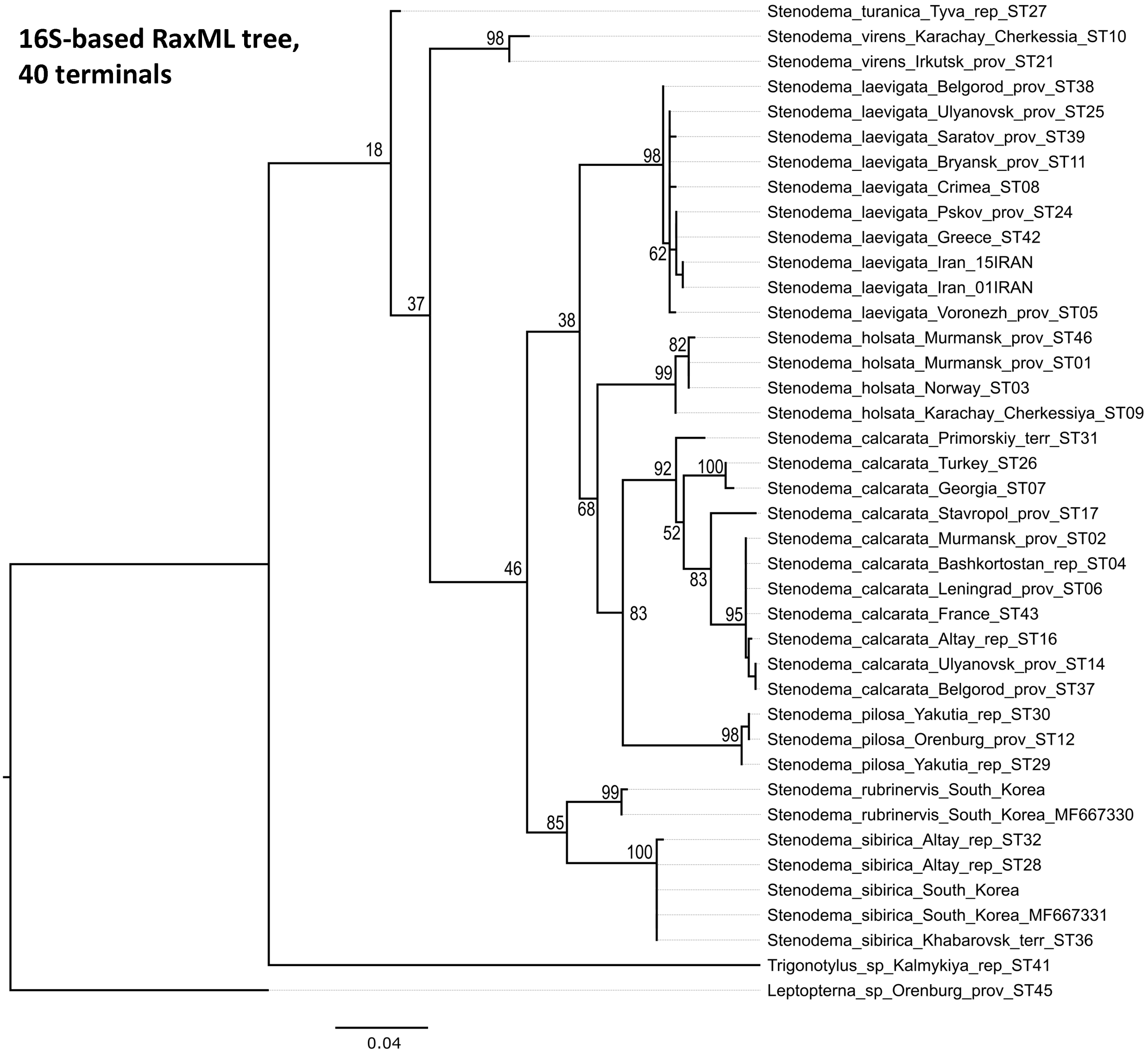
16S-based RaxML tree,
40 terminals
Stenodema_turanica_Tyva_rep_ST27
Stenodema_virens_Karachay_Cherkessia_ST10
Stenodema_virens_Irkutsk_prov_ST21
Stenodema_laevigata_Belgorod_prov_ST38
Stenodema_laevigata_Ulyanovsk_prov_ST25
Stenodema_laevigata_Saratov_prov_ST39
Stenodema_laevigata_Bryansk_prov_ST11
Stenodema_laevigata_Crimea_ST08
Stenodema_laevigata_Pskov_prov_ST24
Stenodema_laevigata_Greece_ST42
Stenodema_laevigata_Iran_15IRAN
Stenodema_laevigata_Iran_01IRAN
Stenodema_laevigata_Voronezh_prov_ST05
Stenodema_holsata_Murmansk_prov_ST46
Stenodema_holsata_Murmansk_prov_ST01
Stenodema_holsata_Norway_ST03
Stenodema_holsata_Karachay_Cherkessiya_ST09
Stenodema_calcarata_Primorskiy_terr_ST31
Stenodema_calcarata_Turkey_ST26
Stenodema_calcarata_Georgia_ST07
Stenodema_calcarata_Stavropol_prov_ST17
Stenodema_calcarata_Murmansk_prov_ST02
Stenodema_calcarata_Bashkortostan_rep_ST04
Stenodema_calcarata_Leningrad_prov_ST06
Stenodema_calcarata_France_ST43
Stenodema_calcarata_Altay_rep_ST16
Stenodema_calcarata_Ulyanovsk_prov_ST14
Stenodema_calcarata_Belgorod_prov_ST37
Stenodema_pilosa_Yakutia_rep_ST30
Stenodema_pilosa_Orenburg_prov_ST12
Stenodema_pilosa_Yakutia_rep_ST29
Stenodema_rubrinervis_South_Korea
Stenodema_rubrinervis_South_Korea_MF667330
Stenodema_sibirica_Altay_rep_ST32
Stenodema_sibirica_Altay_rep_ST28
Stenodema_sibirica_South_Korea
Stenodema_sibirica_South_Korea_MF667331
Stenodema_sibirica_Khabarovsk_terr_ST36
Trigonotylus_sp_Kalmykiya_rep_ST41
Leptopterna_sp_Orenburg_prov_ST45
18
98
37
98
62
38
82
99
46
68
92
100
52
83
95
83
98
99
85
100
0.04

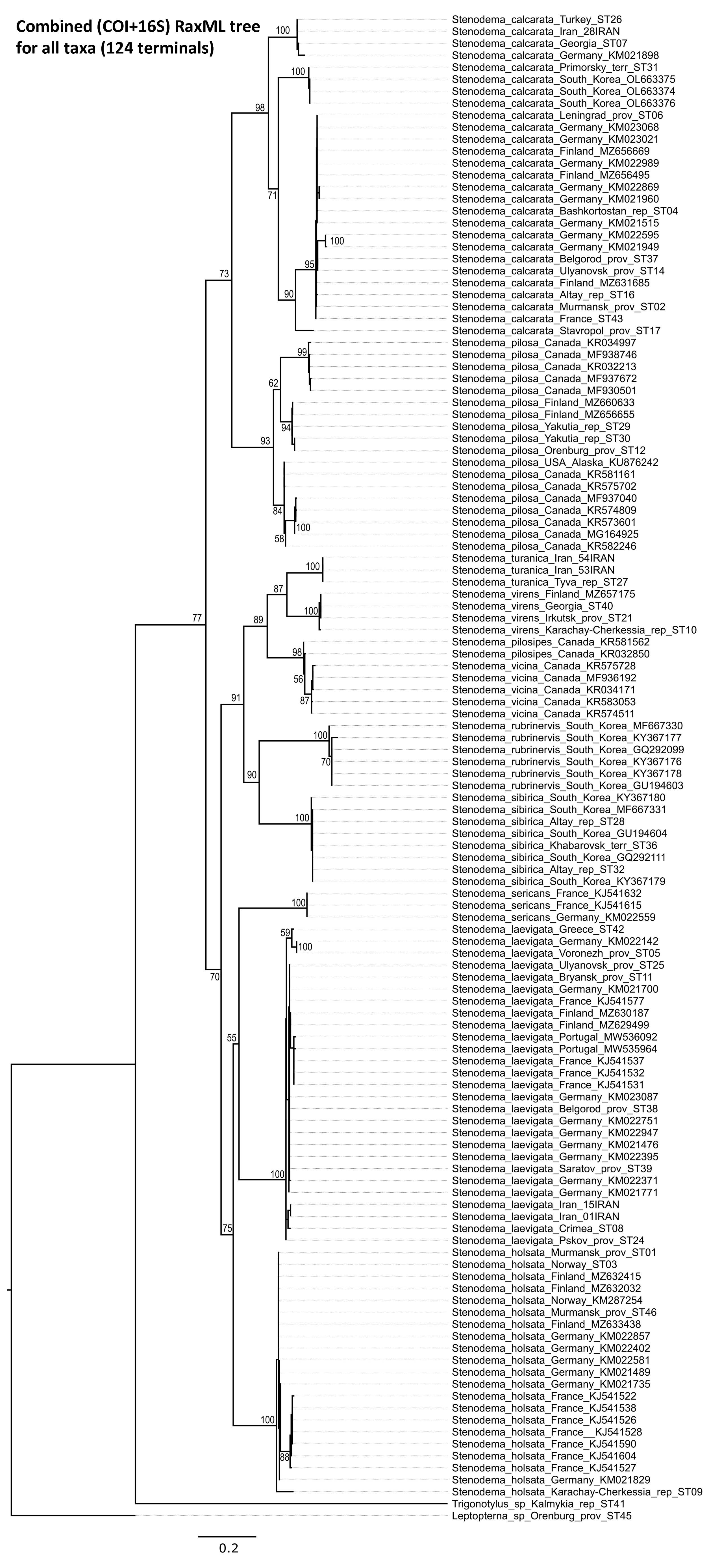
Combined (COI+16S) RaxML tree for all taxa (124 terminals)
Stenodema_calcarata_Turkey_ST26
Stenodema_calcarata_Iran_28IRAN
Stenodema_calcarata_Georgia_ST07
Stenodema_calcarata_Germany_KM021898
Stenodema_calcarata_Primorsky_terr_ST31
Stenodema_calcarata_South_Korea_OL663375
Stenodema_calcarata_South_Korea_OL663374
Stenodema_calcarata_South_Korea_OL663376
Stenodema_calcarata_Leningrad_prov_ST06
Stenodema_calcarata_Germany_KM023068
Stenodema_calcarata_Germany_KM023021
Stenodema_calcarata_Finland_MZ656669
Stenodema_calcarata_Germany_KM022989
Stenodema_calcarata_Finland_MZ656495
Stenodema_calcarata_Germany_KM022869
Stenodema_calcarata_Germany_KM021960
Stenodema_calcarata_Bashkortostan_rep_ST04
Stenodema_calcarata_Germany_KM021515
Stenodema_calcarata_Germany_KM022595
Stenodema_calcarata_Germany_KM021949
Stenodema_calcarata_Belgorod_prov_ST37
Stenodema_calcarata_Ulyanovsk_prov_ST14
Stenodema_calcarata_Finland_MZ631685
Stenodema_calcarata_Altay_rep_ST16
Stenodema_calcarata_Murmansk_prov_ST02
Stenodema_calcarata_France_ST43
Stenodema_calcarata_Stavropol_prov_ST17
Stenodema_pilosa_Canada_KR034997
Stenodema_pilosa_Canada_MF938746
Stenodema_pilosa_Canada_KR032213
Stenodema_pilosa_Canada_MF937672
Stenodema_pilosa_Canada_MF930501
Stenodema_pilosa_Finland_MZ660633
Stenodema_pilosa_Finland_MZ656655
Stenodema_pilosa_Yakutia_rep_ST29
Stenodema_pilosa_Yakutia_rep_ST30
Stenodema_pilosa_Orenburg_prov_ST12
Stenodema_pilosa_USA_Alaska_KU876242
Stenodema_pilosa_Canada_KR581161
Stenodema_pilosa_Canada_KR575702
Stenodema_pilosa_Canada_MF937040
Stenodema_pilosa_Canada_KR574809
Stenodema_pilosa_Canada_KR573601
Stenodema_pilosa_Canada_MG164925
Stenodema_pilosa_Canada_KR582246
Stenodema_turanica_Iran_54IRAN
Stenodema_turanica_Iran_53IRAN
Stenodema_turanica_Tyva_rep_ST27
Stenodema_virens_Finland_MZ657175
Stenodema_virens_Georgia_ST40
Stenodema_virens_Irkutsk_prov_ST21
Stenodema_virens_Karachay-Cherkessia_rep_ST10
Stenodema_pilosipes_Canada_KR581562
Stenodema_pilosipes_Canada_KR032850
Stenodema_vicina_Canada_KR575728
Stenodema_vicina_Canada_MF936192
Stenodema_vicina_Canada_KR034171
Stenodema_vicina_Canada_KR583053
Stenodema_vicina_Canada_KR574511
Stenodema_rubrinervis_South_Korea_MF667330
Stenodema_rubrinervis_South_Korea_KY367177
Stenodema_rubrinervis_South_Korea_GQ292099
Stenodema_rubrinervis_South_Korea_KY367176
Stenodema_rubrinervis_South_Korea_KY367178
Stenodema_rubrinervis_South_Korea_GU194603
Stenodema_sibirica_South_Korea_KY367180
Stenodema_sibirica_South_Korea_MF667331
Stenodema_sibirica_Altay_rep_ST28
Stenodema_sibirica_South_Korea_GU194604
Stenodema_sibirica_Khabarovsk_terr_ST36
Stenodema_sibirica_South_Korea_GQ292111
Stenodema_sibirica_Altay_rep_ST32
Stenodema_sibirica_South_Korea_KY367179
Stenodema_sericans_France_KJ541632
Stenodema_sericans_France_KJ541615
Stenodema_sericans_Germany_KM022559
Stenodema_laevigata_Greece_ST42
Stenodema_laevigata_Germany_KM022142
Stenodema_laevigata_Voronezh_prov_ST05
Stenodema_laevigata_Ulyanovsk_prov_ST25
Stenodema_laevigata_Bryansk_prov_ST11
Stenodema_laevigata_Germany_KM021700
Stenodema_laevigata_France_KJ541577
Stenodema_laevigata_Finland_MZ630187
Stenodema_laevigata_Finland_MZ629499
Stenodema_laevigata_Portugal_MW536092
Stenodema_laevigata_Portugal_MW535964
Stenodema_laevigata_France_KJ541537
Stenodema_laevigata_France_KJ541532
Stenodema_laevigata_France_KJ541531
Stenodema_laevigata_Germany_KM023087
Stenodema_laevigata_Belgorod_prov_ST38
Stenodema_laevigata_Germany_KM022751
Stenodema_laevigata_Germany_KM022947
Stenodema_laevigata_Germany_KM021476
Stenodema_laevigata_Germany_KM022395
Stenodema_laevigata_Saratov_prov_ST39
Stenodema_laevigata_Germany_KM022371
Stenodema_laevigata_Germany_KM021771
Stenodema_laevigata_Iran_15IRAN
Stenodema_laevigata_Iran_01IRAN
Stenodema_laevigata_Crimea_ST08
Stenodema_laevigata_Pskov_prov_ST24
Stenodema_holsata_Murmansk_prov_ST01
Stenodema_holsata_Norway_ST03
Stenodema_holsata_Finland_MZ632415
Stenodema_holsata_Finland_MZ632032
Stenodema_holsata_Norway_KM287254
Stenodema_holsata_Murmansk_prov_ST46
Stenodema_holsata_Finland_MZ633438
Stenodema_holsata_Germany_KM022857
Stenodema_holsata_Germany_KM022402
Stenodema_holsata_Germany_KM022581
Stenodema_holsata_Germany_KM021489
Stenodema_holsata_Germany_KM021735
Stenodema_holsata_France_KJ541522
Stenodema_holsata_France_KJ541538
Stenodema_holsata_France_KJ541526
Stenodema_holsata_France__KJ541528
Stenodema_holsata_France_KJ541590
Stenodema_holsata_France_KJ541604
Stenodema_holsata_France_KJ541527
Stenodema_holsata_Germany_KM021829
Stenodema_holsata_Karachay-Cherkessia_rep_ST09
Trigonotylus_sp_Kalmykia_rep_ST41
Leptopterna_sp_Orenburg_prov_ST45
0.2

Combined (COI+16S) RaxML tree for reduced dataset (34 terminals)

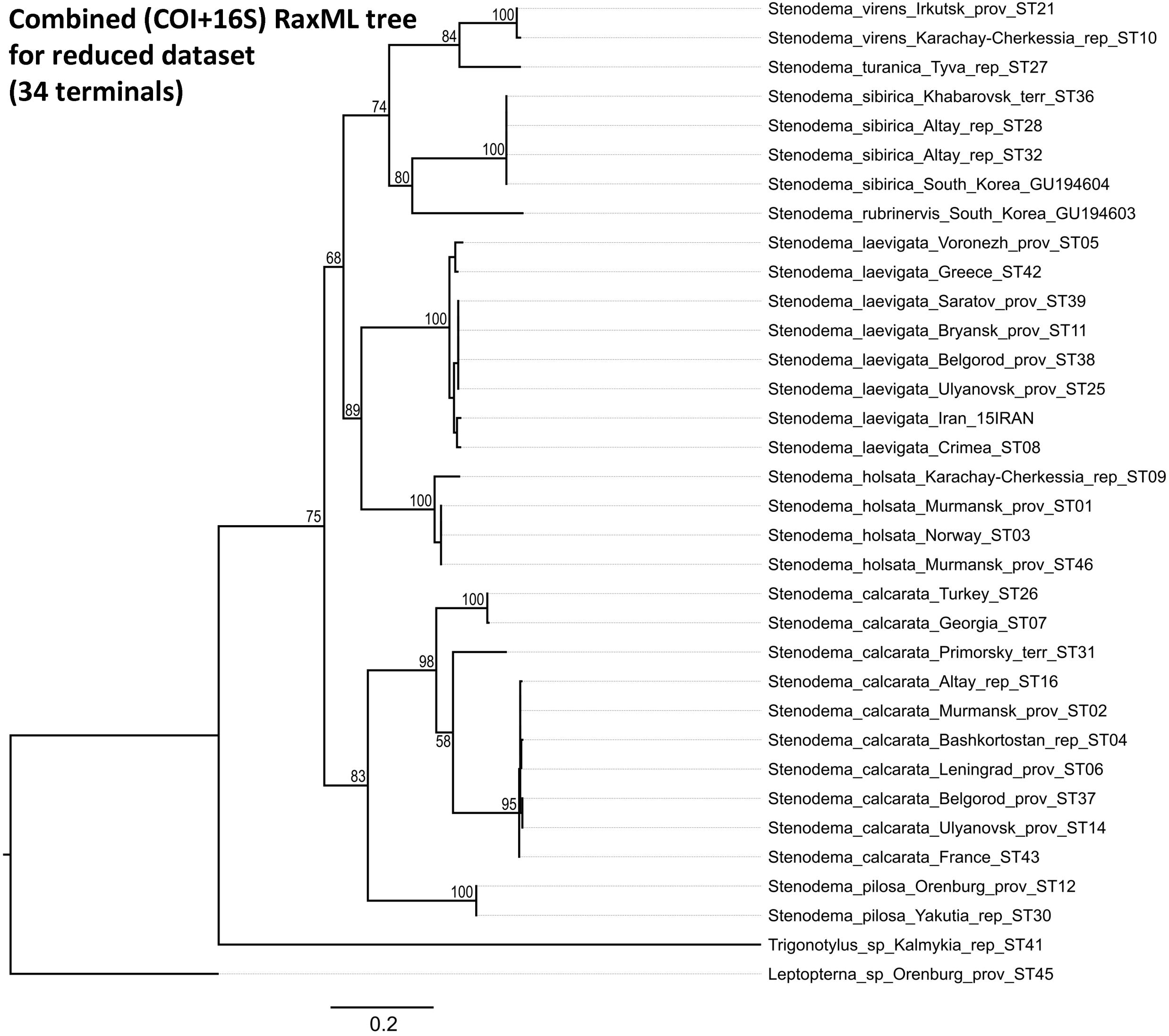

Supplement: Supplementary material 3 — RaxML trees for individual and combined datasets [file zookeys-1209-245_article-124766__-s003.pdf]
