# Supplementary material for: ﻿Delimitation of the widely distributed Palearctic Stenodema species (Hemiptera, Heteroptera, Miridae): insights from molecular and morphological data
Source: Zookeys. 2024 Aug 13;1209:245–94. doi: 10.3897/zookeys.1209.124766 (PMC11336388; doi:10.3897/zookeys.1209.124766)
Supplement: Supplementary material 4 — Genetic distances [file zookeys-1209-245_article-124766__-s004.pdf]

Supplementary material 4.

Interspecific and intraspecific P-distances for COI

	within species distances	between species differences									
		calcarata	*holsata*	*laevigata*	*pilosipes*	*rubrinervis*	*sibirica*	*pilosa*	*turanica*	*sericans*	*vicina*
calcarata	0.0398										
holsata	0.0117	0.1193									
laevigata	0.0085	0.1287	0.0901								
pilosipes	0.0033	0.1111	0.0974	0.0963							
rubrinervis	0.0039	0.1473	0.1178	0.1314	0.1160						
sibirica	0.0006	0.1273	0.1107	0.1227	0.0957	0.1137					
pilosa	0.0365	0.1013	0.1211	0.1146	0.1132	0.1512	0.1283				
turanica	0	0.1193	0.1046	0.1008	0.0729	0.1135	0.1058	0.1147			
sericans	0	0.1361	0.0933	0.0943	0.0961	0.1167	0.1114	0.1175	0.1054		
vicina	0.004	0.1134	0.0959	0.0939	0.0119	0.1114	0.1004	0.1108	0.0704	0.1033	
virens	0.0011	0.1137	0.1080	0.1166	0.0731	0.0991	0.1002	0.1221	0.0622	0.1146	0.0688

Interspecific and intrespecific Kimura-2-parameter distances for COI

	within species distances	between species differences									
		calcarata	*holsata*	*laevigata*	*pilosipes*	*rubrinervis*	*sibirica*	*pilosa*	*turanica*	*sericans*	*vicina*
calcarata	0.0424										
holsata	0.0119	0.1312									
laevigata	0.0086	0.1425	0.0974								
pilosipes	0.0033	0.1210	0.1055	0.1045							
rubrinervis	0.004	0.1667	0.1296	0.1472	0.1280						
sibirica	0.0006	0.1411	0.1211	0.1359	0.1036	0.1251					
pilosa	0.0381	0.1105	0.1333	0.1256	0.1239	0.1720	0.1424				
turanica	0	0.1310	0.1139	0.1097	0.0775	0.1246	0.1153	0.1256			
sericans	0	0.1517	0.1012	0.1022	0.1037	0.1284	0.1219	0.1288	0.1143		
vicina	0.004	0.1239	0.1037	0.1014	0.0120	0.1224	0.1093	0.1208	0.0746	0.1122	
virens	0.0011	0.1240	0.1181	0.1288	0.0780	0.1079	0.1089	0.1348	0.0656	0.1261	0.0731

Interspecific and intraspecific P-distances for 16S rRNA

	within species distances	between species differences						
		calcarata	*holsata*	*laevigata*	*rubrinervis*	*sibirica*	*pilosa*	*turanica*
calcarata	0.0214							
holsata	0.004	0.0695						
laevigata	0.0039	0.0763	0.0679					
rubrinervis	0.0026	0.0828	0.0761	0.0698				
sibirica	0.0011	0.0874	0.0737	0.0712	0.0509			
pilosa	0.001	0.0620	0.0683	0.0733	0.0869	0.0895		
turanica	N/A	0.0928	0.0822	0.0823	0.0710	0.0792	0.0976	
virens	0.0083	0.1008	0.0886	0.0954	0.0736	0.0896	0.1133	0.0460

Interspecific and intraspecific Kimura-2-parameter distances for 16S rRNA

	within species distances	between species differences						
		calcarata	*holsata*	*laevigata*	*rubrinervis*	*sibirica*	*pilosa*	*turanica*
calcarata	0.022							
holsata	0.004	0.0732						
laevigata	0.0039	0.0806	0.0712					
rubrinervis	0.0026	0.0878	0.0802	0.0733				
sibirica	0.0011	0.0930	0.0776	0.0749	0.0527			
pilosa	0.001	0.0648	0.0717	0.0771	0.0924	0.0953		
turanica	N/A	0.0993	0.0872	0.0872	0.0747	0.0838	0.1046	
virens	0.0083	0.1086	0.0944	0.1021	0.0775	0.0955	0.1229	0.0478

P-distances for COI between *Stenodema calcarata* groups

	West Asian group	East Asian group	German group
West Asian group			
East Asian group	0.0675		
German group	0.0801	0.0791	
Euro-Asian group	0.0772	0.0750	0.0145

Kimura-2-parameter distances for COI between *Stenodema calcarata* groups

	West Asian group	East Asian group	German group
West Asian group			
East Asian group	0.0715		
German group	0.0860	0.0851	
Euro-Asian group	0.0826	0.0803	0.0147

P-distances for 16S rRNA between *Stenodema calcarata* groups

	West Asian group	East Asian group	Stavropol Province specimen
West Asian group			
East Asian group	0.03		
Stavropol Province specimen	0.0348	0.0389	
Euro-Asian group	0.0361	0.0377	0.0286

Kimura-2-parameter distances for 16S rRNA between *Stenodema calcarata* groups

	West Asian group	East Asian group	Stavropol Province specimen
West Asian group			
East Asian group	0.0306		
Stavropol Province specimen	0.0359	0.0401	
Euro-Asian group	0.0373	0.0388	0.0292

P-distances for COI between *Stenodema holsata* groups

	Northern and Central European group	France group
Northern and Central European group		
France group	0.0176	
Karachay-Cherkessia specimen	0.0328	0.0396

Kimura-2-parameter distances for COI between *Stenodema holsata* groups

	Northern and Central European group	France group
Northern and Central European group		
France group	0.0179	
Karachay-Cherkessia specimen	0.0339	0.0412

P-distances for16S rRNA between *Stenodema holsata* groups

	Northern and Central European group
Northern and Central European group	
Karachay-Cherkessia specimen	0.00538

Kimura-2-parameter distances for 16S rRNA between *Stenodema holsata* groups

	Northern and Central European group
Northern and Central European group	
Karachay-Cherkessia specimen	0.00541

P-distances for COI between *Stenodema laevigata* groups

	Main European clade	Crimea specimen	Voronezh Province and Germany clade	Greece specimen
Main European clade				
Crimea specimen	0.01225			
Voronezh Province and Germany clade	0.02174	0.02562		
Greece specimen	0.01713	0.02065	0.00953	
Iran specimen	0.01378	0.00889	0.02113	0.01548

Kimura-2-parameter distances for COI between *Stenodema laevigata* groups

	Main European clade	Crimea specimen	Voronezh Province and Germany clade	Greece specimen
Main European group				
Crimea specimen	0.01239			
Voronezh Province and Germany group	0.0222	0.2623		
Greece specimen	0.01741	0.02099	0.00961	
Iran specimen	0.01398	0.00896	0.02156	0.01568

P-distance for COI between *Stenodema laevigata* groups

	Palearctic group	Nearctic group 1	Nearctic group 2
Palearctic group			
Nearctic group 1	0.0456		
Nearctic group 2	0.0455	0.0628	
Nearctic group 3	0.0381	0.0553	0.0162

Kimura-2-parameter distances for COI between *Stenodema laevigata* groups

	Palearctic group	Nearctic group 1	Nearctic group 2
Palearctic group			
Nearctic group 1	0.0475		
Nearctic group 2	0.0475	0.0664	
Nearctic group 3	0.0396	0.0581	0.0165
